# Nanochanneling and Local Crystallization Engineering Accelerate Multiphase Single‐Atom Catalysis for Rapid Water Decontamination

**DOI:** 10.1002/anie.202504571

**Published:** 2025-05-02

**Authors:** Ya Liu, Yuxian Wang, Yunpeng Wang, Jie Miao, Jiajia Yang, Kunsheng Hu, Hongqi Sun, Jiadong Xiao, Chunmao Chen, Xiaoguang Duan, Shaobin Wang

**Affiliations:** ^1^ State Key Laboratory of Heavy Oil Processing China University of Petroleum‐Beijing Beijing 102249 China; ^2^ School of Chemical Engineering The University of Adelaide Adelaide SA 5005 Australia; ^3^ School of Environmental Science and Engineering Nanjing Tech University Nanjing 211816 China; ^4^ School of Molecular Sciences The University of Western Australia Perth WA 6009 Australia; ^5^ School of Chemical Engineering University of Chinese Academy of Sciences Beijing 101408 China

**Keywords:** Carbon crystallinity regulation, Heterogeneous catalytic ozonation, Nanoenvironment engineering, Nonradical reaction, Tri‐phase reaction

## Abstract

Precise engineering of single‐atom catalysts (SACs) with optimal hierarchical structures and favorable local chemical environments remains a significant challenge to cater for multiphase heterogeneous processes. Here, we develop a universal strategy for synthesizing channel‐digging microspherical SACs that markedly enhance gas–liquid–solid mass transfer and fine‐tune the thermodynamics of catalytic ozonation. By catalytically graphitizing carbon microspheres and selectively etching amorphous carbon domains via mild combustion, we fabricate cross‐linked hierarchical graphitic nanochannels confining transition metal (e.g., Co, Cr, Mn, Fe, Ni) single atoms (TMCSs‐Air). This nanoenvironment engineering increases interfacial ozone (O_3_) mass transfer by 3.2‐fold and directs O_3_ adsorption from a conventional “end‐on” to a bidental “side‐on” configuration. The enhanced inter‐orbital electronic interactions lower the O_3_ activation barrier and form highly oxidizing surface‐confined O_3_ (*O_3_). Consequently, the CoCSs‐Air catalyst achieves a 3.6‐fold higher ozone utilization efficiency and a 4.2‐fold greater turnover frequency (TOF = 1580 min^−1^) compared with pristine Co‐doped carbon microspheres (CoCSs). Technical and economic evaluations further confirm the feasibility of TMCSs‐Air nanoreactors in treating real‐world petrochemical wastewater, highlighting its broader potential in overcoming gas diffusion barriers and tuning reaction pathways for multiphase heterogeneous catalysis.

## Graphical abstract

Crystallinity engineering is applied to carbon microspheres for fabricating transition metal single atoms confined in hydrophilic graphitic nanochannels by precisely etching the amorphous carbon realm. This fine‐tuned nanoenvironment enhances tri‐phase mass transfer and optimizes O_3_ nonradical activation thermodynamics by inducing the bidental “side‐on” O_3_ adsorption, which reduces O_3_ activation barrier and accelerates reaction kinetics.

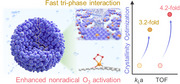

## Introduction

Gas diffusion across the gas–liquid–solid interface, along with subsequent adsorption, activation, and desorption on the solid catalyst surface, is critical in the reaction kinetics and conversion efficiency in tri‐phase catalytic reactions for green energy conversion,^[^ ^1^, ^2^, ^3^ ^]^ sustainable chemical production,^[^ ^4^, ^5^, ^6^ ^]^ and environmental remediation.^[^ ^7^, ^8^, ^9^ ^]^ Developing catalysts with mass transfer‐favorable architectures for enhancing the intrinsic activity of active sites presents a promising and sustainable approach to regulating both kinetics and thermodynamics in tri‐phase reactions.^[^ ^10^ ^]^ Internal mass diffusion within a bulk catalyst, which is strongly influenced by the microstructure surrounding the active sites, can be more pivotal in governing catalytic activity than inter‐phase mass diffusion under practical operating conditions.^[^ ^10^ ^]^ For carbon‐based single‐atom catalysts (SACs), which exhibit maximized atom‐utilization efficiency and strong metal‐support interactions, the nanoenvironment surrounding single‐atom (SA) sites such as local diffusion channels, wettability, and SA density and coordination programs mass transfer, regional kinetics/thermodynamics, and stability of the heterogenous reactions.^[^ ^11^, ^12^, ^13^, ^14^ ^]^ However, the embedded isolated metal centers within the carbon matrix and the micropore‐dominated carbon architectures limit the access of reactants to the catalytic sites, resulting in low utilization efficiency of the reactants and high energy consumption.^[^ ^15^, ^16^, ^17^ ^]^ Engineering the nanoenvironment around the SA sites by constructing porous structures and regulating surface functional properties for optimal metal‐support interaction prominently enhances the accessibility and activity of single atom sites and facilitates the transport of reactants/products in tri‐phase reactions.^[^ ^18^, ^19^ ^]^

Ordered porous particles with short transfer paths and a confined space can remarkably accelerate internal mass diffusion.^[^ ^10^, ^20^, ^21^ ^]^ Nevertheless, uneven mass and heat transfer during the traditional process of carbon pyrolysis often leads to random orientations and rigid cross‐linking of turbostratic nanodomains, resulting in SACs with high heterogeneity containing both amorphous and graphitic domains.^[^ ^22^, ^23^ ^]^ Amorphous domains not only prohibit reactant mass transfer but also impair the intrinsic activity and electron transfer process of the catalysts.^[^ ^24^, ^25^ ^]^ Furthermore, active sites located in graphitic regions typically exhibit more uniform catalytic behavior due to their ordered nature, whereas those embedded in amorphous carbon experience uneven electronic environments, leading to inconsistent activity.^[^ ^26^, ^27^ ^]^ This variability complicates the elucidation of the intrinsic activity of SACs, as local structural differences may obscure their performance. Meanwhile, the wettability of the microenvironment surrounding the active sites governs the kinetics of tri‐phase mass transport.^[^ ^18^ ^]^ Improved hydrophilicity enhances liquid–solid phase interactions and ensures a rapid delivery of dissolved gaseous reactants by facilitating reactant permeation, thereby minimizing interfacial mass transport resistance in multi‐phase catalysis.

Herein, we present a universal nanoenvironment engineering strategy for synthesizing nanoconfined transition metal single atoms (i.e., Co, Cr, Mn, Fe, and Ni) in graphitic carbon microspheres with engineered hierarchical diffusion nanochannels and enhanced metal‐support interaction. Selective etching of amorphous carbon domains, coupled with enhanced surface hydrophilicity, changes the gas–solid reaction mode and ensures significant accessibility of single atom sites on graphitic domains during tri‐phase heterogeneous catalytic ozonation (HCO) reactions for excellent water decontamination. This nanoenvironment engineering switches ozone (O_3_) adsorption from conventional mono‐dental “end‐on” mode to the bidental “side‐on” mode with an enhanced orbital interaction between O_3_ molecules and single atom sites. The high thermodynamic selectivity lowers the O_3_ nonradical activation barrier and increases the oxidation ability of nonradical *O_3_ species, resulting in significantly improved ozone utilization efficiency and excellent catalytic performance. In addition, this nonradical oxidation process was also tested in a large‐scale operating unit, demonstrating high treatment efficiency and economic feasibility for real petrochemical wastewaters from a variety of sources.

## Results and Discussion

### Construction and Characterizations of Hierarchical Graphitic Nanochannels

**Figure 1:**
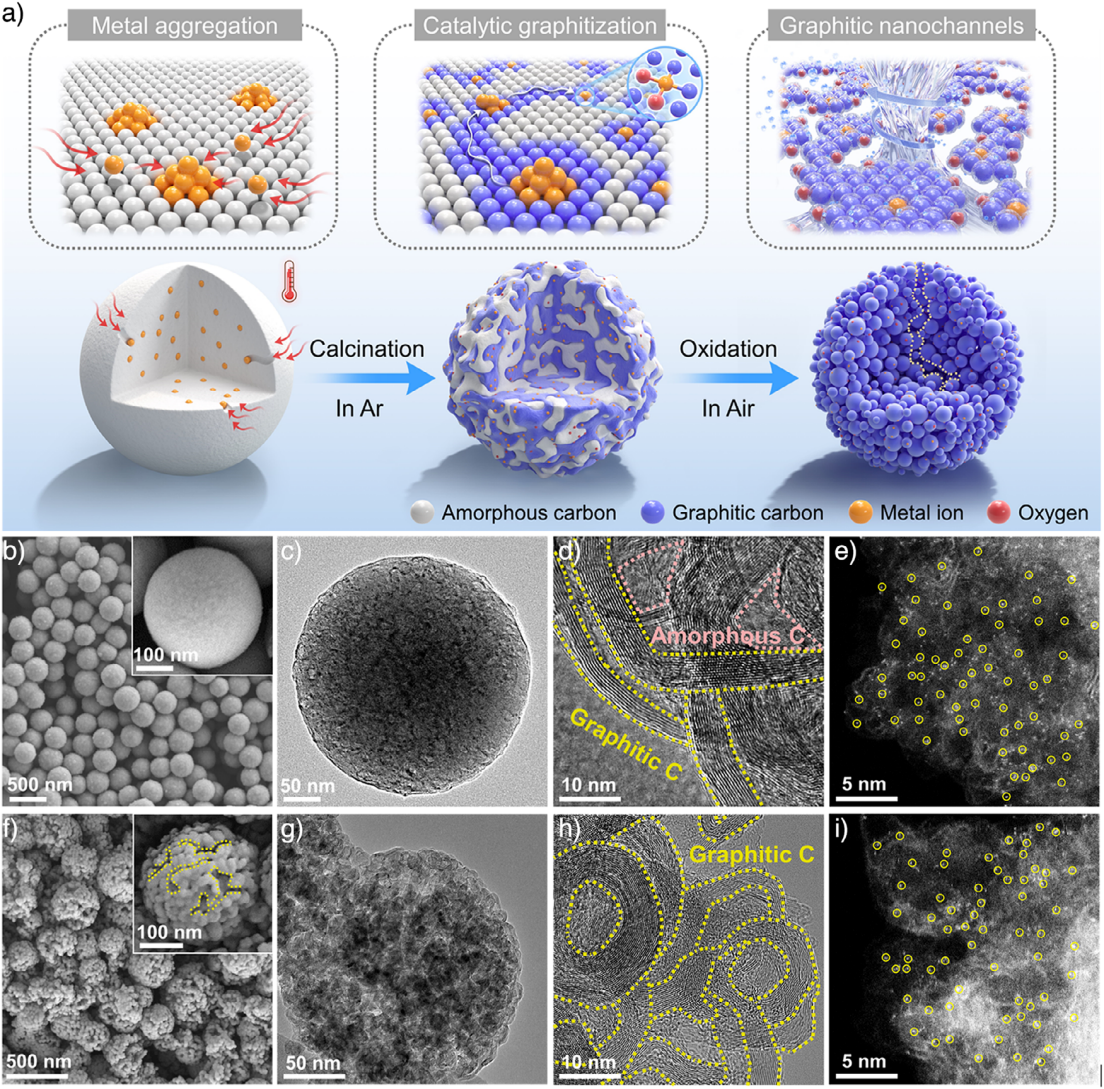
Synthesis and morphology of the catalysts. a) Schematic illustration of the formation process of the CoCSs‐Air material by carbon crystallinity modulation. b) Scanning electron microscopy (SEM) image, c) transmission electron microscopy (TEM) image, d) high‐resolution TEM (HRTEM) image, and e) high‐angle annular dark‐field scanning transmission electron microscopy (HAADF‐STEM) image of CoCSs. f) SEM image, g) TEM image, h) HRTEM image, and i) HAADF‐STEM image of CoCSs‐Air.

The carbon crystallinity modulation strategy for synthesis of transition metal (Co, Cr, Mn, Fe, and Ni)‐doped carbon spheres (TMCSs) is outlined in Figure 1a. Monodisperse amorphous carbon spheres (CSs) with an average diameter of around 400 nm were first synthesized by a hydrothermal method (Figure S1). The rich surface oxygen functional groups (OFGs) and the negatively charged surface of CSs (zeta potential of −28 mV) provide anchoring sites to bind with transition metal (TM) cations via electrostatic‐induced complexations. A subsequent hydrothermal treatment followed by the anaerobic annealing allows the pressure‐driven diffusion of TM ions into the CSs bulk, thus improving the graphitization degree of TMCSs in the pyrolysis step. As a result, a Co‐doped hybrid carbon matrix (CoCSs) was obtained with amorphous domains surrounded by highly graphitized layers (Figure 1b–d).

In contrast, CoCSs‐NH synthesized without the secondary hydrothermal process shows only amorphous‐centric domains, as the trace amounts of incorporated Co^2+^ ions (0.023 wt%, Table S1) on the surface of CSs are insufficient to graphitize amorphous carbon structures at elevated temperatures (Figure S2). The identification of both the low oxidation‐resistant (LOR) exothermal peak at 600 °C and the high oxidation‐resistant (HOR) exothermal peak at 670 °C in differential thermal analyses (DTA) further elucidates the formation of the hybrid carbon matrix in CoCSs (Figure S3),^[^ ^28^ ^]^ which is different from the complete amorphous phase of CoCSs‐NH with only the LOR exothermal peak. The penetrated Co species during the hydrothermal process tend to aggregate into larger Co nanoparticles (NPs) as the annealing temperature increases and can be easily removed by acid etching.^[^ ^29^ ^]^ This is witnessed by the formation of nanocavities in CoCSs calcined at 800 °C (CoCSs‐800, Figure S4), with a negligible Co content (0.002 wt%, Table S1). At annealing temperatures above 900 °C, these Co NPs can migrate within the carbon matrix, driven by the gas produced from carbothermal reactions, and graphitize the surrounding carbon domains (Figures S5–S8), which will be ultimately downsized to the highly dispersed Co single atoms within the graphitic carbon (Figure 1e).^[^ ^29^, ^30^ ^]^

**Figure 2:**
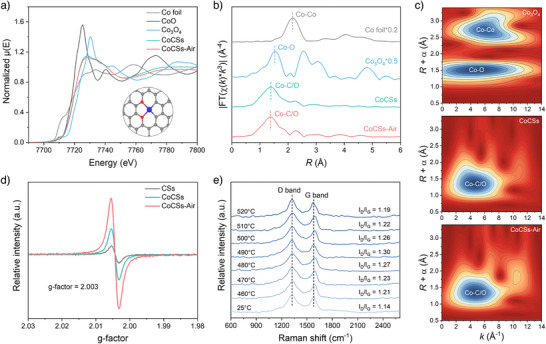
Advanced characterizations of the catalysts. a) XANES of CoCSs, CoCSs‐Air, and reference samples at the Co K‐edge. Inset: schematic model of CoCSs‐Air. The grey, red, and blue spheres represent carbon, oxygen, and cobalt atoms, respectively. b) The corresponding FT‐EXAFS curves at R space. c) EXAFS wavelet transforms of Co K‐edge for Co_3_O_4_, CoCSs, and CoCSs‐Air. d) Cryo‐EPR spectra for CSs, CoCSs, and CoCSs‐Air. e) Temperature‐dependent Raman spectra as the function of the temperature during calcination of CoCSs under static air.

Further post‐combustion in air under lower temperature can selectively remove the less‐stable amorphous domains to create hierarchical graphitic diffusion nanochannels across the carbon spheres. Increasing the annealing temperature from 450 to 520 °C leads to a progressive removal of amorphous carbons from the exterior to the interior regions between graphitic layers in CoCSs (Figure S9), which is evidenced by the gradual release of CO_2_ and H_2_O molecules as the measuring temperature increases in thermogravimetric Fourier‐transform infrared (TG‐FTIR) analysis (Figure S10) and the reduced LOR exothermal peak in DTA curves (Figure S3). A complete removal of amorphous carbon domains in CoCSs at a calcination temperature of 520 °C (CoCSs‐Air) results in self‐assembled hierarchical microspheres with internal crosslinked diffusion nanochannels with dented surface structures (Figure 1f–h and Figures S11 and S12). The remaining graphitized domains reorganized into onion‐like shells with voids between the layers. Such a deliberate removal of amorphous carbon enlarges specific surface area (S_BET_) and mesopore volumes (Figure S13 and Table S2) while retaining the atomic dispersion of Co (Figure 1i and Figure S14). Correspondingly, a two‐fold increase in Co loading is observed in CoCSs‐Air compared to CoCSs (0.022 vs. 0.011 wt%, Table S1). The local coordination environment of Co atoms within the CoCSs‐Air is negligibly affected by carbon domain regulation. CoCSs and CoCSs‐Air adopt quite identical fingerprints in Co K‐edge X‐ray absorption near‐edge structure (XANES) spectra (Figure 2a). The absence of a Co─Co scattering path at ∼2.1 Å for both the catalysts in Fourier‐transform extended X‐ray absorption fine structure (FT‐EXAFS) (Figure 2b) and wavelet transform (WT) analyses (Figure 2c) verify the atomic dispersion of Co sites, while the discerned scattering paths at 1.4 Å can be attributed to the Co─C/O coordinations. Quantitative EXAFS curve‐fitting analyses (Figures S15 and S16 and Table S5) and density functional theory (DFT)‐based modeling results further deciphered that the atomically dispersed Co atoms in both CoCSs and CoCSs‐Air are simultaneously coordinated with two oxygen atoms and two carbon atoms (Co─C_2_O_2_).

Removing amorphous carbon domains also facilitates the exposure of the edging defects of the graphitized carbons. Cryo‐electron paramagnetic resonance (EPR) spectra (Figure 2d) evidence the increased defect level of CoCSs‐Air by post‐aerobic annealing. Moreover, the increase of the *I*_D_/*I*_G_ intensity ratio in temperature‐dependent Raman spectra reveals that more edging sites at the boundaries of graphitic domains are exposed as the annealing temperature increases, leading to a higher defect density (Figure 2e). This was also witnessed by the broad asymmetric peak profiles in X‐ray diffraction (XRD) patterns and the disordered graphitic phase in HRTEM observations (Figures S17 and S18). Noted that the graphitized CoCSs exhibit a higher *I*_D_/*I*_G_ ratio than that of the amorphous CSs. This can be ascribed to the atomically dispersed Co in the graphitic basal plane acting as an extrinsic defect,^[^ ^31^ ^]^ as evidenced by the DFT‐simulated Raman spectra (Figure S19). Rich edge defects are conducive to forming OFGs during mild combustion, resulting in a higher oxygen content in CoCSs‐Air compared to CoCSs (11.3 vs. 3.9 wt%, Table S6). The increased OFGs can enhance surface hydrophilicity, ensuring the accessibility of the single atomic active sites to aqueous reactants. Therefore, the removal of amorphous carbon domains in CoCSs advances the formation of the open‐framework structure of CoCSs‐Air with accessible hierarchical diffusion nanochannels, which facilitates exposure of the catalytic sites and mass transfer of reactants.

This nanoenvironment engineering strategy was also assessed using other TMs (i.e., Cr, Mn, Fe, Ni, Cu). Hierarchical microsphere structures featuring crosslinked graphitic nanochannels are successfully developed for CrCSs‐Air, MnCSs‐Air, FeCSs‐Air, and NiCSs‐Air (Figures S20–S23). However, such a synthesis protocol was not suitable for Cu‐incorporated CSs (Figure S24). This is because the low solubility of carbon in Cu makes it difficult to form metastable copper carbides, which are critical for catalyzing the graphitization process.^[^ ^32^ ^]^

### Effect of Nanochannels on Tri‐Phase Mass Transfer

**Figure 3:**
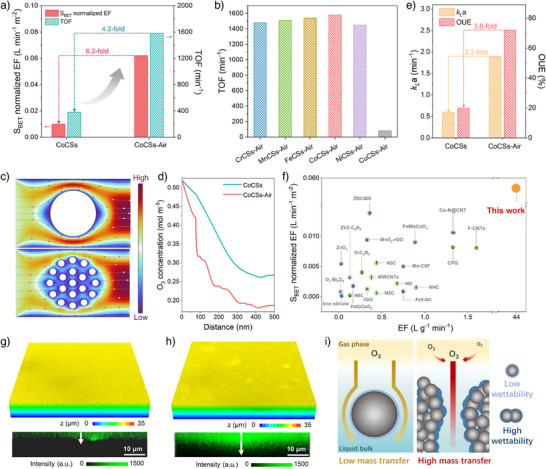
Tri‐phase mass transfer properties of catalysts in HCO. a) S_BET_ normalized EF values and TOF values of CoCSs and CoCSs‐Air. b) TOF values of various TMCSs‐Air samples. c) The simulated cross‐sectional velocity profiles and d) concentration distribution of O_3_ along the streamline in the constructed models with and without diffusion nanochannels. e) *k_L_* a and OUE of CoCSs and CoCSs‐Air. Reaction conditions: catalyst loading: 0.01 g L^−1^; [OA]_0_: 150 mg L^−1^; ozone flow rate: 100 mL min^−1^; ozone concentration: 15 mg L^−1^; temperature: 25 °C; initial pH: 3.0. f) Comparison of the catalytic ozonation performance of our CoCSs‐Air catalyst with previous reports. Confocal 3D reconstruction images (up) and cross‐sectional fluorescence images (below) of g) CoCSs and h) CoCSs‐Air. i) The schematic illustration of the O_3_ mass transfer behavior in CoCSs (left) and CoCSs‐Air (right).

The activity of CoCSs‐based catalysts was evaluated in the heterogeneous catalytic ozonation (HCO) of oxalic acid (OA), a probe molecule inert to direct ozonation (k<0.04 M^−1^ s^−1^) but mineralizable by reactive oxygen species (ROS) generated from O_3_ activation.^[^ ^33^ ^]^ Although CoCSs and CoCSs‐Air obtained high S_BET_, poor OA adsorption was observed (Figure S25), which can be ascribed to the electrostatic expelling force between the negatively charged surface of catalysts (Figure S26) and the deprotonated OA (pK_a1_ = 1.23, pK_a2_ = 4.19) at pH 3. The benchmark catalysts (amorphous CSs, sp^3^‐hybridized carbon‐dominated nanodiamond, and sp^2^‐hybridized carbon dominated graphite) displayed poor HCO activities for OA oxidation at a low catalyst dosage (0.01 g L^−1^), revealing the marginal contribution of the carbonaceous supports to HCO (Figure S27). In contrast, CoCSs significantly enhanced OA oxidation via HCO, resulting in a S_BET_ normalized Efficacy Factor (**EF**, a descriptor for activity, see detailed calculations in Text S10) of 0.01 L min^−1^ m^−2^ (Figure 3a), which positively correlated with the cobalt content (Figure S28). The negligible activity of Co‐doped nanodiamond (Co─ND) excludes the role of Co atoms in amorphous carbon (Figure S27). This suggests the strong metal‐support interactions between the dispersed Co single atoms and the graphitic carbon support. The hierarchical diffusion nanochannels constructed in CoCSs‐Air further led to a 6.2‐fold increase in EF (0.062 vs. 0.01 L min^−1^ m^−2^), and a 4.2‐fold increase in turnover frequency (TOF, 1580 vs. 380 min^−1^) compared to CoCSs (Figure 3a), demonstrating the improved intrinsic activity of the single‐atomic Co sites via spontaneous surface and structural engineering.^[^ ^34^, ^35^ ^]^ Among various TMCSs‐Air samples, Co, Cr, Mn, Fe, and Ni‐based CSs‐Air exhibited comparable catalytic activities, while CuCSs‐Air, without graphitic nanochannels, exhibited inferior activity (Figure 3b and Figure S29).

The effect of solution pH on catalytic performance was also evaluated. Increasing the initial pH significantly reduced catalytic efficiency. The enhanced electrostatic repulsion between the deprotonated catalyst surfaces and the negatively charged OA at elevated solution pHs would greatly hinder their interactions and subsequently decrease the degradation efficiency (Figure S30). Given the high self‐decomposition rate of O_3_ molecules in neutral or alkaline environments, we selected an initial solution pH of 3 to ensure the high catalytic performance of the catalysts and a high ozone utilization efficiency (OUE).^[^ ^36^ ^]^ Additionally, for both CoCSs and CoCSs‐Air, the concentrations of dissolved cobalt ions in the aqueous phase after the reaction were found to be negligible (∼5 µg L^−1^), as confirmed by inductively coupled plasma mass spectrometry (ICP‐MS) measurements. This trivial amount of the leached cobalt ions induced marginal homogeneous ozone activation, contributing to the overall catalytic efficiency (Figure S31).

Finite element analysis based on reaction‐involved COMSOL Multiphysics simulations reveals the enhanced triple‐phase mass transfer in CoCSs‐Air.^[^ ^37^, ^38^ ^]^ A model with open mass diffusion nanochannels obtained a higher internal O_3_ conversion rate than the bulk spherical structure (Figure 3c–d), because the enhanced fluidity of aqueous solution through the diffusion nanochannels expedited O_3_ diffusion in the solution and intensified their interactions with the cobalt single sites in the channels (Figures S32–S34). The greatly higher overall volumetric mass transfer coefficient (*k_L_* a) for CoCSs‐Air compared to CoCSs (1.9 vs. 0.6 min^−1^, Figure 3e) accounted for the enhanced internal mass transfer kinetics within these diffusion nanochannels even at low catalyst dosages. We further measured the variations in liquid‐phase O_3_ concentrations to decode the tri‐phase mass transfer efficiency and O_3_ decomposition capability of the CoCSs and CoCSs‐Air. The liquid phase O_3_ concentration was kept at a low level (< 0.1 mg L^−1^) for CoCSs‐Air (Figure S35) because of its increased diffusion capacity and the reduced activation energy (12.7 vs. 20.1 kJ mol^−1^, Figure S36).^[^ ^7^, ^39^ ^]^ The significant O_3_ concentration gradient between gas and liquid phases stimulates external mass transfer and promotes continuous gaseous O_3_ dissolution in solution. As a result, CoCSs‐Air exhibited a 3.6‐fold increase in OUE compared to CoCSs (Figure 3e), and EF of CoCSs‐Air also outperformed other reported carbon‐based catalysts, SACs, metal oxides, and supported metal oxides (Figure 3f and Table S7). The superior performance of CoCSs‐Air highlights the critical role of hierarchical diffusion nanochannels in improving interfacial mass transfer and catalytic oxidation efficiency.

Hydrophilicity of catalyst interfaces controls the three‐phase mass transfer kinetics of O_3_ toward active sites (from gas to liquid and then to solid surface).^[^ ^9^, ^19^ ^]^ Distinguishing the wettability behaviors of the catalysts helps unveil the enhancement in kinetics by constructing hierarchical diffusion nanochannels. Contact angle (CA) analysis reveals that CoCSs‐Air exhibited a significantly lower water CA than CoCSs (26 vs. 64°, Figure S37). The improvement in hydrophilicity can be attributed to the abundant hydrophilic OFGs (e.g., ─OH and ─COOH) distributed within the diffusion nanochannels of CoCSs‐Air (Table S4). Additionally, the oxygen contents of CoCSs‐Air samples increased with the elevation of the second annealing temperature from 480 to 520 °C (Table S6), while the CAs demonstrated the opposite trend (Figure S38). To investigate the relationship between the oxygen contents of CoCSs‐Air samples and their CAs, a linear plot was constructed. The strong correlation between oxygen contents of the catalysts and CAs signifies that the increase in surface oxygen functional groups markedly enhanced the wettability of catalysts (Figure S39). Furthermore, the CAs demonstrated high regression linearity with the EFs of CoCSs‐Air samples, suggesting the crucial role of hydrophilicity in the greatly enhanced activity of CoCSs‐Air.

To directly observe the hydrophilicity variations at the reaction interfaces and delineate the water phase boundary morphology, we performed confocal laser scanning microscopy (CLSM, Figure S40).^[^ ^19^, ^40^ ^]^ The 3D reconstructions and cross‐sectional fluorescence images (Figure 3g–h) showcase an extended fluorescence decay distance in CoCSs‐Air than CoCSs (11.81 vs. 2.97 µm, Figure S41), indicating the improved liquid phase infiltration within the internal cross‐linked nanochannels to accelerate the mass transfer. Hence, the enhanced hydrophilicity facilitates the accessibility of dissolved O_3_ to the atomic Co sites and their fast reaction. The O_3_ concentration gradient further accelerates the dissolution of O_3_ through the gas–liquid interface, resulting in the improvement of OUE and reaction kinetics (Figure 3i). A radar diagram was also established to elucidate the influences of the physiochemical properties of the catalysts on their catalytic efficiencies (Figure S42). The enhanced graphitic level by selectively removing the amorphous carbon increased S_BET_ and oxygen content, which improved the mass transfer rate and the surface hydrophilicity. The fine‐tuning of these physiochemical properties in CoCSs‐Air collectively contributed to the enhancement of OUE, EF, and TOF.

### Effect of Nanochannels on Tri‐Phase Reaction Thermodynamics

**Figure 4:**
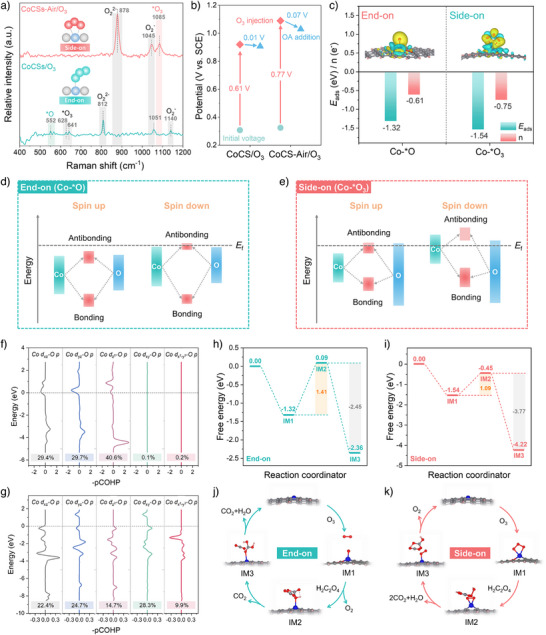
Mechanism investigation. a) In situ Raman spectra of CoCSs and CoCSs‐Air. b) Variations in open‐circuit potentials by in situ electrochemical analysis for CoCSs‐ and CoCSs‐Air‐induced nonradical HCO processes. c) Differential charge densities and the corresponding *E*_ads_ and n in Co─*O and Co─*O_3_. Illustration of orbital interactions between Co 3d and O 2p orbitals in d) Co─*O and e) Co─*O_3_. pCOHP analysis (spin‐down channel) of Co─O bond for f) Co─*O and g) Co─*O_3_. Energy profiles of O_3_ activation and subsequent OA oxidation for h) “end‐on” configuration and i) “side‐on” configuration. Proposed catalytic cycles for O_3_ activation and subsequent OA oxidation for j) “end‐on” configuration and k) “side‐on” configuration.

Building hierarchical graphitic diffusion nanochannels in CoCSs‐Air by selectively removing the blocked amorphous carbon not only altered the diffusion kinetics but also tuned the O_3_ activation pathway. In situ spin‐trapping EPR spectra, radical quenching tests, and probe‐based fluorescence microscopy observations exclude the generation of free radical species and singlet oxygen (^1^O_2_) by ozone activation on CoCSs or CoCSs‐Air (Figures S43 and S44). Therefore, nonradical O_3_ activations probably occurred for both the catalysts and surface‐confined nonradical species accounted for OA oxidation. The in situ Raman spectra revealed a characteristic peak at 552 cm^−1^ in the CoCSs/O_3_ system, attributed to the surface‐adsorbed atomic oxygen (^*^O) from O_3_ catalytic dissociation (Figure 4a).^[^ ^41^ ^]^ Contrarily, no such peak but a surface‐adsorbed O_3_ (^*^O_3_) signal at 1085 cm^−1^ is detected in the CoCSs‐Air/O_3_ system,^[^ ^42^ ^]^ implying the O_3_ molecule remained structurally intact without dissociation. Moreover, the interplays between O_3_ and active sites in the nanochannels of CoCSs‐Air result in a greater oxidation potential than that on the bare surface of CoCSs, invoking a more significant potential drop upon OA addition (Figure 4b and Figure S45). The corresponding in situ chronoamperometry (i‐t) tests further witnessed the enhanced mass transfer efficiency of gaseous O_3_ to the surface of CoCSs‐Air and promoted surface interaction (Figure S46). Additionally, the significant current elevation observed upon the OA addition reveals the intense electron transfer from OA to the surface of CoCSs‐Air, resulting in the rapid oxidation of OA. This surface‐mediated electron transfer process for OA oxidation is also facilitated by the decreased charge‐migration resistance (R_ct_) of CoCSs‐Air (Figure S47 and Table S8).^[^ ^35^ ^]^ Noteworthy, these surface reactive species (^*^O_3_ and ^*^O) can further react with O_3_ to form less oxidative ROS, such as peroxide (O_2_^2−^) and superoxide (O_2_^•−^) species in the absence/insufficiency of pollutants, which can be discerned in in situ Raman spectra.^[^ ^43^, ^44^ ^]^

To decipher the intrinsic origins of different O_3_ activation pathways and thermodynamic selectivity, we built curvature carbon models with atomically dispersed Co in the Co─C_2_O_2_ according to the decoded results from EXAFS analysis and used in DFT simulation. For CoCSs with hybrid carbon domains, the O_3_ molecule interacts with amorphous carbon primarily through weak van der Waals forces rather than forming covalent bonds (Figure S48). As a result, the amorphous carbon within CoCSs acts as a coking agent, impeding O_3_ adsorption and weakening the electronic interaction between O_3_ and the active sites on the graphitic carbon (Figure S49). Combined with in situ Raman spectra and transition state analysis, O_3_ preferentially adsorbs onto CoCSs via a mono‐dental coordinated “end‐on” configuration, bonding with its terminal oxygen atom to the Co site and subsequently dissociates into surface‐confined ^*^O (forming a Co─^*^O complex) and a ground state O_2_ (Figures S50 and S51). In comparison, removing the coking amorphous domains in CoCSs‐Air improves the electronic communications between the graphitic carbon and O_3_ and enhances the binding strength. In CoCSs‐Air, the O_3_ molecule tends to selectively adsorb via a bidental coordinated “side‐on” configuration at the Co─C_2_O_2_ site with two terminal oxygen atoms in O_3_ spontaneously bonding to a Co atom (Co─^*^O_3_ complex),^[^ ^45^, ^46^ ^]^ promoting the orbital interactions and electronic communications between the Co─C_2_O_2_ and the adsorbed O_3_ molecule. The stronger adsorption energy (*E*_ads_) and greater electron transfer (n) of the “side‐on” configuration compared to the “end‐on” configuration enable Co─*O_3_ complex to be more activated than Co─*O complex (Figure 4c).

The formation of Co─*O_3_ complex greatly stretches the in‐plane Co─O/Co─C bonds compared to Co─*O complex (Table S9), promoting their orbital overlapping and interactions, thus elevating the oxidation capacity of the nonradical species. Projected density of state (PDOS) profiles suggest the highly geometrical distortion of the Co center in Co─*O_3_ complex invokes an enlarged magnetic moment of the Co atom and thus increases the spin polarization of the 3d orbitals of Co (Figures S52–S55).^[^ ^47^, ^48^ ^]^ This enables the spin‐down channel of Co 3d orbital as the predominant factor in forming an out‐plane Co─O bond with the adsorbed O_3_, as revealed by the crystal orbital Hamilton population (COHP) analysis (Figure S56).^[^ ^49^ ^]^ Accordingly, the strong spin polarization upshifts the antibonding orbitals of the spin‐down channel in Co─*O_3_ complex above the Fermi level (*E*_f_), realigning the filled electrons in antibonding at the *E*_f_ and raising the Co d‐band center (Figure 4d–e and Figure S57). As a result, Co 3d sub‐orbitals in Co─*O_3_ complexation are more localized around *E*_f_ with a higher electron density than those in Co─*O configuration (Figures S58–S60). The enhanced orbital hybridization between Co and O atoms near the *E*_f_ facilitates electron transfer in Co─*O_3_ complex, thus greatly elevates the oxidation potential and thus intensifying the reactivity of *O_3_ species. Quantitative analysis of the projected COHP (pCOHP) then detailed the bonding behaviors of the Co 3d sub‐orbitals with O 2p orbitals in O_3_ (Figures S61 and S62 and Table S14), with spatial distributions modeled by maximally localized Wannier functions (MLWF) (Figures S63–S66).^[^ ^47^, ^50^ ^]^ Unlike the Co─*O complex, where Co $d_{z^{2}}$, d_xz_, and d_yz_ orbitals primarily contribute to Co─O bonding, and the Co─*O_3_ complex also engaged Co d_xy_ and $d_{x^{2}-y^{2}}$ orbitals with high energy levels because of the bridged adsorption configuration of O_3_ (Figure 4f–g). The elevated levels of antibonding orbitals in Co─*O_3_ expedite electron transfer in the redox process.

O_3_ adsorption in “side on” configuration also regulates the OA oxidation pathway because of its higher oxidation capacity. Transition state analysis revealed that “end‐on” model exhibited a lower reaction energy barrier (Δ*E*_a_) and more negative Gibbs free energy (Δ*G*) during OA oxidation (Figure 4h–i), indicating that OA oxidation is more thermodynamically favorable with the Co─*O_3_ complex.^[^ ^51^, ^52^ ^]^ Furthermore, a more intense orbital overlap is observed from PDOS profiles between the Co, O_3_, and OA orbitals in “side‐on” model than “end‐on” model, solidifying the strong electron transfer from OA to the Co─*O_3_ complex (Figure S67). For final state products, the “end‐on” model achieves a free CO_2_ and a Co─H_2_CO_3_ complex, which ultimately evolve into CO_2_ and H_2_O, regenerating the active sites (Figure 4j). In contrast, the adsorbed O_2_ in the Co─*O_2_ configuration is released as a free O_2_ molecule in the “side‐on” model (Figure 4k). Also, the tailored O_3_ activation pathway in reducing side‐product formation contributed to an elevated OUE for OA oxidation (Figures S68 and S69).

### Practical Applications

To assess the applicability of HCO triggered by CoCSs‐Air in real‐world wastewater treatment, we evaluated total organic carbon (TOC) removal efficiency for a diverse range of organic pollutants, including phenolics, antibiotics, dyes, pesticides, phthalate acid esters, and pharmaceutical and personal care products (PPCPs). Results demonstrate that CoCSs‐Air significantly enhanced the TOC removal efficiency across all the tested pollutants compared to the single ozonation process (Figure S70). Additionally, the catalytic performance of CoCSs‐Air/O_3_ system was marginally affected by the presence of different inorganic anions (Cl^−^, HCO_3_^−^, NO_3_^−^, and H_2_PO_4_^−^) and natural organic matter (NOM, Figure S71), revealing the high selectivity and robust anti‐interference capability of the nonradical *O_3_ species. Unlike CoCSs, which was completely passivated after 20 min (Figures S72–S74), the highly graphitic nature of CoCSs‐Air preserves its structure integrity and surface properties against oxidative etching (Figures S75 and S76), securing the stability in HCO.

**Figure 5:**
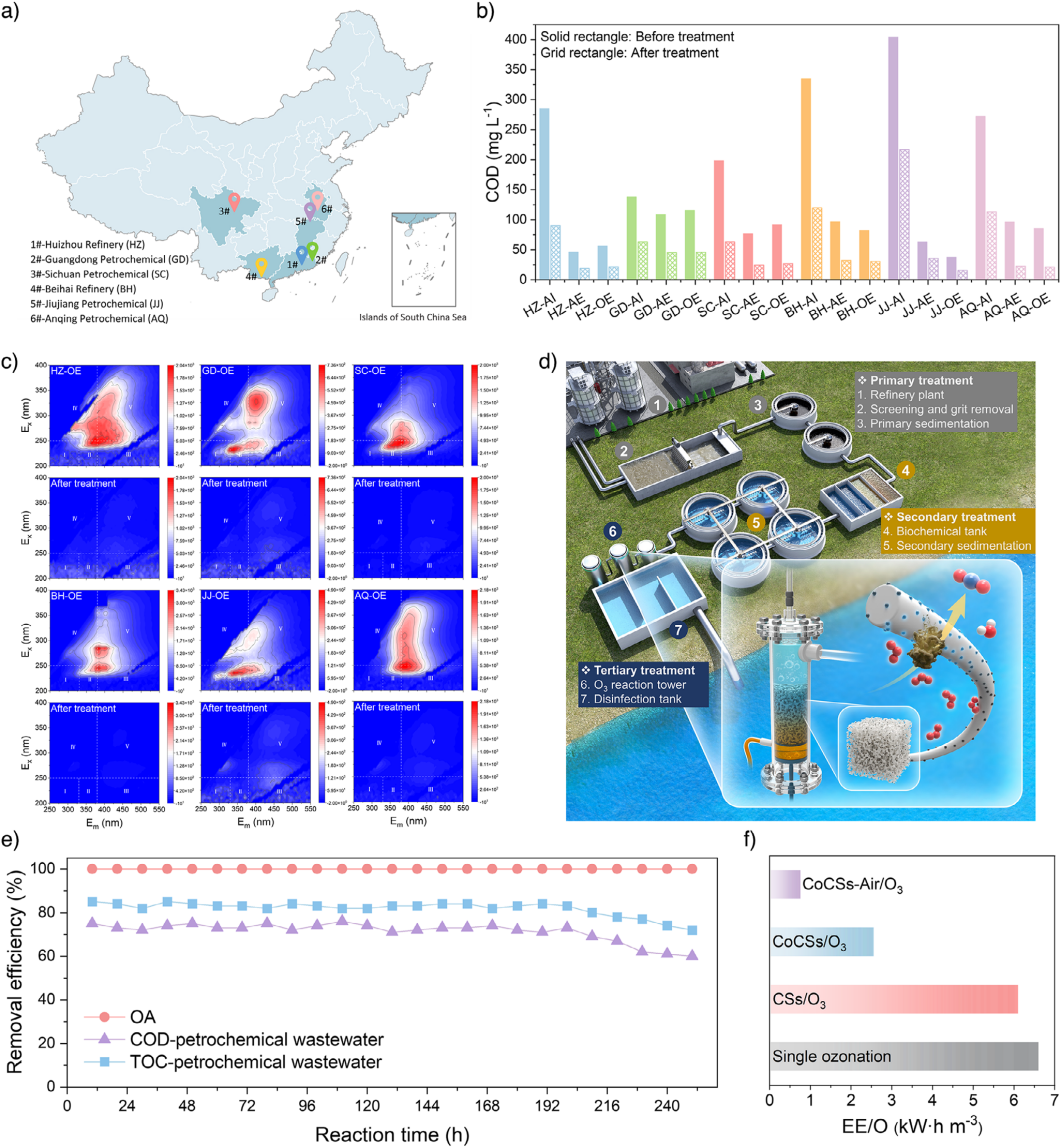
Integrated water purification device and continuous operation. a) Sources of 18 PCW samples and the distribution of six petrochemical enterprises in China. b) COD removals of eighteen PCW samples after catalytic ozonation treatment. (AI, AE, and OE are defined as anaerobic unit influent, anaerobic unit effluent, and aerobic unit effluent in each refinery station, respectively.) c) 3D‐EEM spectra of six PCW samples before and after CoCSs‐Air/O_3_ treatment in 30 min. d) The schematic illustration of the proposed wastewater treatment system. e) OA, COD, and TOC removal efficiency of PCW in the continuous flow system. Reaction conditions: wastewater flow rate: 1.2 L h^−1^; ozone flow rate: 500 mL min^−1^; ozone concentration: 25 mg L^−1^; [OA]_0_: 150 mg L^−1^; temperature: 25 °C. f) Comparison of EE/O for different reaction systems.

The efficacy of CoCSs‐Air/O_3_ system in the treatment of real petrochemical wastewater (PCW) was then evaluated using 18 wastewater samples from six petrochemical enterprises located along the Yangtze River and the southern coast of China (Figure 5a and Text S16). Due to the complex components in PCW, the chemical oxygen demand (COD) levels after biochemical treatment cannot meet the standards required for wastewater discharge (Table S15). Moreover, the pH of each point source of real petrochemical wastewater was adjusted to 3 to achieve high treatment efficiency. The CoCSs‐Air/O_3_ system demonstrates high performance in purifying real wastewater. Especially, for all aerobic unit effluents (OE), the COD values were well reduced to below 60 mg L^−1^ after 30‐min treatment (Figure 5b and Table S16), meeting the discharge standard of pollutants for petroleum refining industry in China (GB 31570–2015, COD < 60 mg L^−1^). According to time‐depedent UV_254_ variation profiles, the CoCSs‐Air/O_3_ system effectively removed aromatic contaminants from PCW (Figure S77). Additionally, three‐dimensional excitation‐emission matrix (3D‐EEM) fluorescence spectroscopy shows that nearly all 3D‐EEM peaks for the treated effluents disappeared (Figure 5c), indicating the excellent capacity to remove refractory fluorescence dissolved organic matters (DOMs) from the real wastewater. Furthermore, its practical utility was evaluated by a device‐level demonstration with a total treatment volume of 35 L (Figure 5d and Figure S78). At a flow rate of 1.2 L h^−1^, complete OA oxidation was achieved in 250‐h operation (Figure 5e). Furthermore, CoCSs‐Air exhibited remarkable efficacy in treating PCW, with TOC and COD removal efficiencies exceeding 70% over 200 h of continuous operation.

Additionally, the EE/O (electrical energy per order) concept, provided by the International Union of Pure and Applied Chemistry (IUPAC), was utilized to assess electrical energy consumption and further investigate the economic applicability of the proposed systems (Figure 5f and Text S17). Assuming an electricity cost of 0.07 USD kW·h^−1^, as published by the State Grid Corporation of China, the EE/O cost for CoCSs‐Air/O_3_ system was 0.0532 USD m^−3^, which is significantly lower than that of single ozonation (0.463 USD m^−3^) and CoCSs/O_3_ system (0.179 USD m^−3^, Table S17). To fully estimate the cost of CoCSs‐Air/O_3_ system, we also evaluated the expenditure for catalyst fabrication (0.02 USD m^−3^, Table S18). These results strongly manifest the technical and economic feasibility of integrating CoCSs‐Air/O_3_ system into upscaled devices after the secondary biochemical treatment for practical wastewater treatment.

## Conclusion

In summary, we presented a generic nanoenvironment engineering strategy to synthesize microspherical SACs with enhanced tri‐phase mass transfer ability and thermodynamic selectivity for nonradical catalytic ozonation of water pollutants. Hierarchical graphitic diffusion nanochannels within the SACs are created through precise etching of the amorphous domains, which significantly enhance the exposure of SA sites and facilitate internal diffusion. The abundant oxygen functional groups on defect‐rich graphitic carbon further improve surface wettability and trans‐phase mass transfer, making the active sites more accessible for superior catalysis. This nanoenvironment engineering remarkably enhances O_3_ mass transfer by 3.2‐fold and fine‐tunes the O_3_ activation pathway, making O_3_ preferentially adsorb in a bidental “side‐on” configuration on the active Co─C_2_O_2_ SA sites rather than the conventional mono‐dental “end‐on” configuration. The high thermodynamic selectivity in CoCSs‐Air with promoted inter‐orbital electronic communications between Co─C_2_O_2_ SA sites and the “side‐on” adsorbed O_3_ decreases the O_3_ nonradical activation barrier, achieving a 3.6‐fold increase in OUE and a 4.2‐fold increase in TOF (1580 min^−1^) compared to the pristine CoCSs. We further validate its practical application by constructing an upscaled device, demonstrating both technical and economic feasibility in real PCW treatment. This work highlights an innovative strategy for nanoenvironment engineering and elaborates on mechanistic insights into carbon crystallinity‐regulated catalytic behaviors to mitigate gas diffusion barriers and modulate reaction thermodynamics in multi‐phase catalysis, yielding potential applications in large‐scale environmental cleanup.

## Conflict of Interests

The authors declare no conflict of interest.

## Supporting information



Supporting Information

## Data Availability

The data that support the findings of this study are available from the corresponding author upon reasonable request.
