# Supplementary material for: Nanochanneling and Local Crystallization Engineering Accelerate Multiphase Single‐Atom Catalysis for Rapid Water Decontamination
Source: Angew Chem Int Ed Engl. 2025 May 2;64(27):e202504571. doi: 10.1002/anie.202504571 (PMC12207383; doi:10.1002/anie.202504571)
Supplement: Supplementary file 1 — Supporting Information [file ANIE-64-e202504571-s001.docx]

Supporting Information

for

**Nanochanneling and Local Crystallization Engineering Accelerate Multiphase Single-Atom Catalysis for Rapid Water Decontamination**

Ya Liu^a,b^, Yuxian Wang^a*^, Yunpeng Wang^b^, Jie Miao^c^, Jiajia Yang^a^, Kunsheng Hu^b^, Hongqi Sun^d*^, Jiadong Xiao^e^, Chunmao Chen^a^, Xiaoguang Duan^b*^ and Shaobin Wang^b*^

*^a^ State Key Laboratory of Heavy Oil Processing, China University of Petroleum-Beijing, Beijing 102249, China.*

*^b^ School of Chemical Engineering, The University of Adelaide, Adelaide, SA 5005, Australia.*

*^c^ School of Environmental Science and Engineering, Nanjing Tech University, Nanjing 211816, China.*

*^d^ School of Molecular Sciences, The University of Western Australia, Perth, WA 6009, Australia.*

*^e^ School of Chemical Engineering, University of Chinese Academy of Sciences, Beijing 101408, China.*

^*^ To whom correspondence may be addressed. E-mail: *yuxian.wang@cup.edu.cn,* [*hongqi.sun@uwa.edu.au*](mailto:hongqi.sun@uwa.edu.au)*,* [*xiaoguang.duan@adelaide.edu.au*](mailto:xiaoguang.duan@adelaide.edu.au)*, and shaobin.wang@adelaide.edu.au.*

**This file includes:**

Pages **112**

Supporting Text **S1**-**S17**

Figures **S1**-**S78**

Tables **S1**-**S18**

SI References **41**

**Experimental Procedures**

**Text S1. Chemicals and Reagents.** Glucose (99.9%), cobalt chloride hexahydrate (CoCl_2_**^.^**6H_2_O, 99.99%), nickel chloride hexahydrate (NiCl_2_**^.^**6H_2_O, 99.99%), ferric chloride hexahydrate (FeCl_3_**^.^**6H_2_O, 99.99%), manganese chloride tetrahydrate (MnCl_2_**^.^**4H_2_O, 99.99%), copper chloride dihydrate (CuCl_2_**^.^**2H_2_O, 99.99%), oxalic acid (OA, 99.9%), 4-nitrophenol (4-NP, 99.0%), 4-chlorophenol (4-CP, 99.0%), 4-chlorobenzoic acid (pCBA, 99.0%), p-hydroxybenzoic acid (pHBA, 99.0%), sulfamethoxazole (SMX, 99.0%), humic acid (HA, 98%), methanol (99.0%), sodium chloride (99.0%), sodium bicarbonate (99.0%), sodium sulfate (99.0%), sodium phosphate (99.0%), sodium nitrate (99.0%), sodium hydroxide (99.0%), 5,5-dimethyl-1-pyrroline (DMPO, 99.0%), 2,2,6,6-tetramethyl-4-piperidone (TEMP, 99.0%), dimethyl sulfoxide (DMSO, 99.0%), coumarin (99.0%), phosphoric acid (85.0%) and sodium dihydrogen phosphate (99.99%) were purchased from Aladdin Biochemical Reagent Co., Ltd. Sulfuric acid (98.0%) and hydrochloric acid (37.0%) were obtained from Beijing Chemical Plant. Potassium indigotrisulfonate (99.0%), N, N-diethyl-p-phenylenediamine (98.5%), horseradish peroxidase (95.0%) and acetonitrile (99.99%) were purchased from Sigma-Aldrich. All the chemicals were utilized without further purification.

**Text S2. Catalyst Preparation.** CSs was were synthesized by a hydrothermal method.^[1]^ Typically, 7.24 g D-glucose was dissolved in 70 mL of ultrapure water by ultrasonicating for 30 min. Then the solution was transferred into a 100 mL Teflon-lined stainless steel autoclave and hydrothermally treated at 180 °C for 18 h. The as-obtained brown precipitate was washed by deionized water for 3 times. After dried at 80 °C overnight, the CSs were obtained for further uses.

CoCSs were fabricated by the following procedures. CSs (0.3 g) were first dispersed in 150 mL ultrapure water by ultrasonication for 1 h. Subsequently, 0.6 mL of CoCl_2_**^.^**6H_2_O aqueous solution (3 mg mL^-1^) was dropwise added to the CSs suspension under magnetic stirring at room temperature, followed by sonicating for 30 min. The precursor suspension was then hydrothermally treated at 180 °C for 6 h in a 200 mL Teflon-lined stainless-steel autoclave. After filtering and drying, the derived Co^2+^-CSs product was further transferred into the tube furnace and heated at 1000 °C for 2 h in Ar with a ramping rate of 5 °C min^-1^. The obtained product was washed with 1 M HCl solution at 60 °C overnight to leach out any aggregated metal clusters and particles. The CoCSs thus obtained were subsequently washed with ultrapure water and dried at 80 °C overnight. In accordance with the annealing temperature in the tube furnace (800, 900 and 1000 °C), the as-synthesized products were denoted as CoCSs-800, CoCSs-900 and CoCSs-1000, respectively. Additionally, according to the adding amount of CoCl_2_**^.^**6H_2_O solution (0.3, 0.6, 1, and 2 mL), the as-obtained samples were denoted as CoCSs-0.3, CoCSs-0.6, CoCSs-1 and CoCSs-2, respectively.

CoCSs-Air was prepared by annealing the CoCSs in air at 520 °C for 2 h with a ramping rate of 5 °C min^-1^. The synthesis procedure of various TMCSs-Air (TMs = Cr, Mn, Fe, Ni, Cu) is the same as that of CoCSs-Air, except that CoCl_2_**^.^**6H_2_O was substituted by CrCl_2_**^.^**6H_2_O, MnCl_2_**^.^**4H_2_O, FeCl_2_**^.^**4H_2_O, NiCl_2_**^.^**6H_2_O, and CuCl_2_**^.^**2H_2_O for preparing CrCSs-Air, MnCSs-Air, FeCSs-Air, NiCSs-Air, and CuCSs-Air, respectively.

**Text S3. Characterization Methods.** Scanning electron microscopy (SEM) was performed with a field emission ZEISS GeminiSEM 300 at an accelerating voltage of 5 kV. Transmission electron microscopy (TEM) and corresponding energy-dispersive X-ray spectroscopy (EDX) elemental mappings were acquired on Talos F200S. The images of single atoms were obtained by a high-angle annular dark-field scanning transmission electron microscopy (HAADF-STEM) (JEM ARM-200F, JEOL). X-ray diffraction (XRD) (PAN analytical B.V., X’Pert-PROMPD) was operated at an accelerated voltage of 40 kV and an emission current of 40 mA with Cu Kα radiation (λ = 1.5418 Å). The Raman spectra were taken by LabRAM HR Evolution (HORIBA). The temperature-dependent Raman spectroscopy was conducted in air with a heat rate of 5 °C min^-1^. N_2_ adsorption/desorption isotherms were recorded via a Micrometrics Tristar 300. A ThermoFisher Scientific ESCALAB 250Xi spectrometer was used to record the X-ray photoelectron spectroscopy (XPS) spectra. The metal contents were conducted on the Agilent 7500ce inductively coupled plasma mass spectrometry (ICP-MS). Thermogravimetry differential thermal analysis (TG/DTA) (TGA/DSC1 STAR^e^ system, METTLER-TOLEDO) and thermogravimetric Fourier transform infrared spectroscopy (TG-FTIR) (NETZSCH STA-2500, Thermo Scientific IS50) were conducted in air with a heat rate of 10 °C min^-1^. Zeta potentials of the samples were recorded via a Delsa Nano C spectrometer (Beckman Coulter). X-ray absorption structure (XAS) including X-ray absorption near-edge structure (XANES) and extended X-ray absorption fine structure (EXAFS) of the samples were collected at the Australian Synchrotron. Zero field cooled (ZFC) measurement was performed on a superconducting quantum interference device (SQUID) magnetometer (MPMS-XL) under H=1 kOe for temperature-dependent (2-400 K) magnetizations measurement. Electron paramagnetic resonance (EPR, Bruker, EMX) was employed to investigate the types of reactive oxygen species (ROS) during the catalytic oxidation using 5,5-dimethyl-1-pyrroline (DMPO) and 2,2,6,6-tetramethyl-4-piperidone hydrochloride (TEMP) as the spin-trapping agents. In catalytic ozonation process, saturated O_3_ solution at pH 3 was used (~ 4 mg L^-1^ of dissolved O_3_) instead of continuously bubbling O_3_ to the reaction solution. EPR operating conditions: centerfield: 3510 G; sweep width: 100 G; microwave frequency: 9.057 GHz; modulation frequency: 100 GHz; power: 18.32 mW. The element compositions of the materials were characterized with an elemental analyzer (Elementar, vario EL cube). The fluorescence excitation-emission matrix (EEM) spectra were characterized using a spectrofluorometer (F-7000FL, Hitachi) by scanning at excitation wavelengths in the range of 200-400 nm (5 nm steps) and emission wavelengths in the range of 250-550 nm (2 nm steps).

**Text S4. Evaluation of Catalytic Performance.** Catalytic ozone oxidation of aqueous organics was conducted in a 550 mL semi-batch glass reactor at 25 °C. Ozone was generated from ultrapure oxygen (99.99%) by an ozone generator (Anseros COM-AD-02, Germany) and monitored by an on-line ozone detector (Anseros Ozomat GM, Germany). The reaction system contained 500 mL of solution with 100 ppm OA and 0.1 g L^-1^ of catalyst. Unless specified, ozone concentration at the inlet was set as 15 mg L^-1^ and initial pH of the reaction solution was adjusted to 3 by 0.01 M H_2_SO_4_/NaOH. In a typical test, the generated gaseous mixture of O_3_ and O_2_ was continuously bubbled through a plate diffuser into the bottom of the reactor with a flow rate of 100 mL min^-1^, which was regulated by a mass flowmeter (DO7-7, Sevenstar, China). The temperature was kept within 25 ± 1 °C. Samples were withdrawn from the reactor at certain time intervals and filtered through a 0.22 μm PTFE filter. The filtrate (1 mL) was then quenched by 0.5 mL sodium nitrite solution (0.1 M) to consume the unreacted ozone and ROS. The OA concentration was analyzed by an ultrahigh-performance liquid chromatograph (UHPLC, Thermofisher U3000 series) equipped with an Acclaim Organic Acid (OA) column. The mobile phase was 0.1% H_3_PO_4_ and the flow rate was set as 0.8 mL min^-1^. TOC was analyzed by an organic carbon analyzer (TOC-L, Shimadzu, Japan). The dissolved O_3_ in the reaction solution was determined by the Indigo blue method.^[2]^ H_2_O_2_ in solution was measured by the horseradish peroxidase method.^[3]^ Key experiments were performed in triplicates and the results are presented in mean values with standard derivations.

**Text S5. *In Situ* Raman Measurement.** In situ Raman spectra were carried out on a confocal microscopic Raman spectrometer (LabRAM HR Evolution) with a 532 nm solid-state laser. In a typical process, 0.1 g catalyst powder was added to saturated O_3_ solution and was mixed thoroughly. Then, 0.5 mL aliquots were immediately collected, placed onto the microscope slide, and scanned from 300 to 1200 cm^-1^ at a resolution of 1 cm^-1^ and a duration time of 100 s.

**Text S6. Contact Angle (CA) Measurement.** The water CA was measured on a Dataphysics OCA20 system in ambient air at room temperature. In the experiments, 2 μL of water was dropped on the surface of samples and the CA images were taken after 5 s. The bubble CA was measured by the captive bubble method (Dataphysics OCA20) and the volume of the gas bubble was about 3 μL for each testing. The average CA was obtained by measuring more than five different positions of the same sample.

**Text S7. Confocal Laser Scanning Microscopy (CLSM) Measurement.** CLSM measurements were carried out on an N-C2-SIM (Nikon, Japan). A fluorescein-labeled aqueous solution (100 μL, 0.1 mM) was deposited onto a confocal dish, followed by placing a 1 × 1 cm^2^ C-GDL onto the aqueous surface (as schematically shown in Fig. S40). The C-GDL was formed by immobilizing catalysts on the carbon paper (H14C9, Freudenberg, Germany). A 405 nm laser was used as the excitation light source, with the confocal microscope being equipped with a 10× objective lens.

**Text S8. Fluorescence Microscopy Image (FMI) Measurement.** An inverted fluorescence microscope (Nikon N-C2-SIM, Japan) was utilized to obtain the FMI images (UV excitation, 365 nm). In a typical step, the sample was withdrawn from the catalytic ozonation reactor and immediately mixed with prepared 1 mM coumarin solution at a volume ratio of 1:1. Approximately 1 mL of the mixture was then extracted by a glue-head dropper and transferred to a glass slide for image capturing/analysis.

**Text S9. Electrochemical Measurement.** Electrochemical measurements were undertaken using an electrochemical workstation (CHI 760D, CH Instrument) in a conventional three-electrode configuration, including a working electrode (Φ3 mm glassy carbon electrode), a counter electrode (platinum electrode), and a reference electrode (saturated silver chloride electrode). A Na_2_SO_4_ aqueous solution (0.5 M) was used as the electrolyte. Typically, the working electrodes were prepared as follows: 5 mg catalyst was mixed with 4 mL isopropanol and 100 μL Nafion by ultrasonication for 2 h. The resulted ink (5 μL) was dropped on the disk electrode and dried in an oven at 60 °C for 30 min. The EIS spectra were measured with a frequency range of 10^-2^ to 10^5^ Hz with a 10 mV amplitude.

**Text S10. Calculation of Efficacy Factor (EF) and Turnover Frequency (TOF).**

The EF value was calculated as follows:

$$\text{EF = }\frac{\text{concentration of contaminant }\left( \text{mg }\text{L}^{\text{-1}} \right)\text{ }\text{× \% of its elimination}}{\text{O}_{\text{3}}\text{ concentration }\left( \text{mg }\text{L}^{\text{-1}} \right)\text{ }\text{× catalyst loading }\left( \text{g} \right)\text{ × time (min)}}$$

$$\text{S}_{\text{BET}}\text{ normalized EF = }\frac{\text{EF}}{\text{S}_{\text{BET}}\text{ (}\text{m}^{\text{2}}\text{ }\text{g}^{\text{-1}}\text{)}}$$

Where, degradation (%) indicates the degradation efficiency of OA, which can be calculated by $\frac{\text{C}_{\text{0}}\text{-C}}{\text{C}_{\text{0}}}\text{×100}$, where, C_0_ and C are the initial and final concentrations of OA (mg L^-1^), respectively.

The TOF value was calculated as follows:

$$\text{TOF = }\frac{\text{number of pollutants moles }\text{× \% of its degradation}}{\text{number of active sites }\text{× time (min)}} \text{ }$$

Where, the number of active sites is defined as the content of metal ions in a specific amount of the catalyst in the reaction. Time means the reaction time for the initial to the final of the reaction (min).

**Text S11. Calculation of Ozone Utilization Efficiency (OUE) and the Molar Ratio of Consumed O_3_ to Degraded OA (*R*).**

The OUE was calculated as follows:

$$\text{OUE = }\frac{{\text{[}\text{O}_{\text{3}}\text{]}}_{\text{consumed}}}{{\text{[}\text{O}_{\text{3}}\text{]}}_{\text{input}}}\text{=}\frac{\int_{\text{0}}^{\text{t}} \left[ \text{O}_{\text{3}} \right]_{\text{in}}\text{dt}\text{-}\int_{\text{0}}^{\text{t}} \left[ \text{O}_{\text{3}} \right]_{\text{offgas}}\text{dt}\text{-}\int_{\text{0}}^{\text{t}} \left[ \text{O}_{\text{3}} \right]_{\text{solution}}\text{dt}}{\int_{\text{0}}^{\text{t}} \left[ \text{O}_{\text{3}} \right]_{\text{in}}\text{dt}}$$

Where, ${\text{[}\text{O}_{\text{3}}\text{]}}_{\text{consumed}}$ represents the amount of consumed O_3_ during reaction time, ${\text{[}\text{O}_{\text{3}}\text{]}}_{\text{input}}$ is the amount of input O_3_ during reaction time, $\left[ \text{O}_{\text{3}} \right]_{\text{in}}$ is the amount of inlet O_3_, $\left[ \text{O}_{\text{3}} \right]_{\text{offgas}}$ is the amount of O_3_ in the offgas and $\left[ \text{O}_{\text{3}} \right]_{\text{solution}}$ is the amount of O_3_ in the reaction solution. All of the items in “[ ]” represent the mass of O_3_.

The *R* was calculated as follows:

$$\text{R}\text{ = }\frac{\text{n}{\text{[}\text{O}_{\text{3}}\text{]}}_{\text{consumed}}}{\text{n}\text{[OA]}_{\text{degraded}}}$$

Where, $\text{n}{\text{[}\text{O}_{\text{3}}\text{]}}_{\text{consumed}}$ represents the molar amount of consumed O_3_ and $\text{n}\text{[OA]}_{\text{degraded}}$ is the molar amount of degraded OA.

**Text S12. Calculation of Volumetric Mass Transfer Coefficient (*k_L_*a).**

The *k_L_*a was calculated as follows:

$$\text{ln (C}\text{*}-\text{C) = }-\text{K}_{\text{L}}\text{a × t +}\ln\text{(C*)}$$

Where, C* is the saturated dissolved ozone concentration (mg L^-1^), C is the actual dissolved ozone concentration (mg L^-1^) and t is the aeration time (min)

**Text S13. Calculation of Activation Energy (E_a_).**

The E_a_ was calculated by the Arrhenius equation:

$$\text{ln }\text{k}\text{ = }-\frac{\text{E}_{\text{a}}}{\text{RT}}\text{ +}\ln\text{A}$$

Where, *k*, A, E_a_ and T represent the reaction rate constant (min^-1^), pre-exponential factor, activation energy (J mol^-1^) and temperature (K), respectively.

**Text S14. Finite Element Method (FEM) Simulations.** The FEM simulations were performed with the COMSOL Multiphysics, and the corresponding 3D mode was collected according to the experiment data. The water was transport from the left to right boundary in the 3D simulation domain. The inflow velocity was set to 0.25 m s^-1^ at the entrance. The “Chemistry” model was used to define the HCO reactions. Both the adsorption and catalytic reaction were surface reactions. The inlet concentration of O_3_ was set as 25 mg L^-1^. The diffusion constants of O_3_, O_2_ and CO_2_ were set to be 1.9 × 10^-9^ m^2^ s^-1^, 1.0 × 10^-9^ m^2^ s^-1^ and 1.0 × 10^-9^ m^2^ s^-1^, respectively. The concentration for the distribution of reactant species as a dependent variable was done on the “Transport of Diluted Species” module.

The mass diffusion was calculated by the Fick’s second law equation:

$$\text{∇}\text{ ∙}\text{ }\left( \text{-D}\text{∇}\text{c} \right)\text{ + }\text{u }\text{∙ }\text{∇}\text{c = R}$$

Where, c represents the concentration of species (mol m^-3^), D is the diffusion coefficient (m^2^ s^-1^), $\mathbf{u}$ is the convective velocity (m s^-1^), and R is the expression for the rate of matter (mol m^-3^·s^-1^).

**Text S15. Computational Details.** Density function theory (DFT) calculations were conducted using the projector augmented plane-wave (PAW) basis, which was implemented in a Vienna *ab-initio* simulation package (VASP).^[4]^ The plane-waves were cut-off at 550 eV. The exchange-correlations of electrons were described by the generalized gradient approximations with the form proposed by Perdew, Burke, and Ernzerhof.^[5]^ DFT-D3 approach was utilized to capture the long-range van der Waals interactions.^[6]^ Vacuum layers of 15 Å were set in all directions, which are supposed to be nonperiodic to eliminate the interactions between images. The energy converge criterion for solving self-consistent Kohn-Sham equations was 10^-5^ eV. Bader charge analysis was employed to count the localized atomic charges.^[7]^ The projected crystal orbital Hamilton populations (pCOHP) ^[8]^ were calculated by the computer program Local Orbital Basis Suite Toward Electronic-Structure Reconstruction (LOBSTER).^[9]^ The calculation of Maximally localized Wannier functions (MLWFs) are done with the basis of linear combination of atomic orbitals (LCAO), which is implemented in OPENMX package.^[10]^ The numerical pseudo-atomic orbitals (PAO) are with cutoff 5.0 Bohr and include radial functions s2p2d1 for C, s2p1 for H, s2p2d1 for O and s3p2d1 for Co. The exchange-correlations of electrons are described by the generalized gradient approximations with the form proposed by Perdew, Burke, and Ernzerhof.^[5]^ The energy converge criterion for solving self-consistent Kohn-Sham equations is 10^-7^ Ha.

The adsorption energy of O_3_ was calculated as follows:

E_ads_ = E_tot_ – E_sub_ – $\text{E}_{\text{O}_{\text{3}}}$

Where, E_tot_ is the total energy of adsorbed system, and E_sub_ and $\text{E}_{\text{O}_{\text{3}}}$terms are the energies of substrate and isolated O_3_ molecule.

**Large-Scale Application and Economic Evaluation**

**Text S16. Details on Petrochemical Wastewater and Continuous Low Tests**

1. Petrochemical wastewater

The petrochemical wastewater (PCW) samples are sourced from six petrochemical enterprises: Huizhou Refinery (HZ), Guangdong Petrochemical (GD), Sichuan Petrochemical (SC), Beihai Refinery (BH), Jiujiang Petrochemical (JJ), and Anqing Petrochemical (AQ). The wastewater samples were collected from secondary treatment units employing the anaerobic-oxic (A/O) process. Specifically, the wastewater samples used in the experiment come from the anaerobic unit influent (AI), anaerobic unit effluent (AE), and aerobic unit effluent (OE) in each refinery. The main characteristics of the PCW are summarized in Table S15.

(2) Continuous flow tests

A continuous flow device consists of three units, including a gas generating unit, a wastewater treatment unit, and a control unit (Fig. S78). The gas generating unit utilized air as a source. Ozone was prepared by an ozone generator (COM-AD-02, Anseros, Germany) using oxygen from an oxygen generator. The ozone output was controlled by a mass flowmeter (DO7-7, Sevenstar, China) and an ozone detector (GM-6000-OEM, Anseros, Germany). The reactor in wastewater treatment unit was composed of a Pyrex tube with an internal diameter of 150 mm, a height of 600 mm, and a working volume of 5 L. A porous acrylic plate located at 100 mm from the bottom supports the catalyst filler (diameter 2 mm). Ozone was injected into the reactor through a plate diffuser from the bottom at a flow rate of 0.5 L min^-1^ and a concentration of 25 mg L^-1^. The tail gas was discharged from the outlet and emitted outdoors after treatment. Wastewater flowed into the reactor from the bottom to the top at a flow rate of 1.2 L h^-1^, driven by a peristaltic pump (Perista pump BT100-2J, Baoding Lange Constant Flow Pump Co., LTD, China). The control unit includes online pH monitoring (pH160, Hangzhou Mei Control Automation Technology Co., LTD, China), gas flow display and regulation, and backwash control. Backwash water was pumped into the reactor through a magnetic pump (MP-F-204CCV5, Kunshan National Treasure Filter Co., LTD, China) to wash the catalyst filler.

To deposit catalysts on polyurethane sponges, we immersed the sponges in aqueous solution with dispersed catalyst powders. The solution was then ultrasonicated and dried at 80 °C overnight. To avoid the escape of the catalyst powder from the sponges during the continuous-flow operation, we immersed the sponges obtained above in pure water repeatedly for several times to remove loose bound materials. The catalyst-loaded sponges were then loaded into the reactor.

**Text S17. Details on Economic Evaluation**

(1) Calculation of expense of electrical energy

The electrical energy per order (EE/O, kW·h m^-3^) is defined as the electrical energy (kW·h) required to reduce the concentration of contaminants by one order of magnitude in one cubic meter of contaminated water.^[11]^ This figure-of-merit serves as a crucial reference for scaling up processes, conducting economic analyses, and comparing energy efficiency across different treatment technologies. The EE/O and expense of electrical energy (E_EE/O_) was calculated from the following equations.

$$\text{EE/O}\text{ }\text{= }\frac{\text{ P}\text{∙ }\text{t}\text{ ∙ 1000}}{\text{60 ∙ }\text{V }\text{∙}\log\text{(}\frac{\text{C}_{\text{i}}}{\text{C}_{\text{e}}}\text{)}}$$

$$\text{E}_{\text{EE/O}}\text{ = }\text{E}_{\text{Price}}\text{∙ }\text{EE}\text{/}\text{O}$$

Where, *EE/O* is the electrical energy per order (kW·h m^-3^), *P* is the rated power (kW) of the catalytic ozonation system, *t* is the reaction time (min), *V* is the volume (L) of the PCW solution in the reactor, and *C_i_* and *C_e_* are the influent and effluent COD, respectively. The values of 1000 and 60 are the conversion factors from liters to m^3^ of the reactor volume and hours to minutes of the reaction time, respectively. *E_EE/O_* is the expense of electrical energy (USD m^-3^), *E_Price_* is the cost of electricity published by State Grid Corporation of China (0.07 USD kW·h^-1^).

(2) Calculation of catalyst fabrication cost

The cost of catalyst fabrication consisted of reagent cost (*R_cost_*) and electricity expense (*E_Cost_*). Additionally, all cost of the recovery process originated from the utilization of electricity. Moreover, the details and results of calculation were listed in Table S18.

$$\text{R}_{\text{cost}}\text{ = }\text{R}_{\text{Price}}\text{∙ }\text{Dosage}\text{/}\text{Q}$$

$$\text{Electricity}\text{ = }\text{P}\text{ ∙ }\text{t}\text{ /}\text{Q}$$

$$\text{E}_{\text{cost}}\text{ = }\text{E}_{\text{Price}}\text{∙ }\text{Electricity}$$

Where, *R_cost_* is the reagent cost (USD m^-3^), *R_Price_* is the unit-price of different reagents (USD g^-1^), *Dosage* is the reagent dosage (g), *Electricity* is the power consumption of various instruments (kW·h), *P* is the input power of instruments (kW), *t* is the operating time (h), *n* is the recovery times, *Q* is the volume of PCW solution (m^3^), *E_Cost_* is the expense of electricity (USD m^-3^), and *E_Price_* is the cost of electricity published by State Grid Corporation of China (0.07 USD kW·h^-1^).


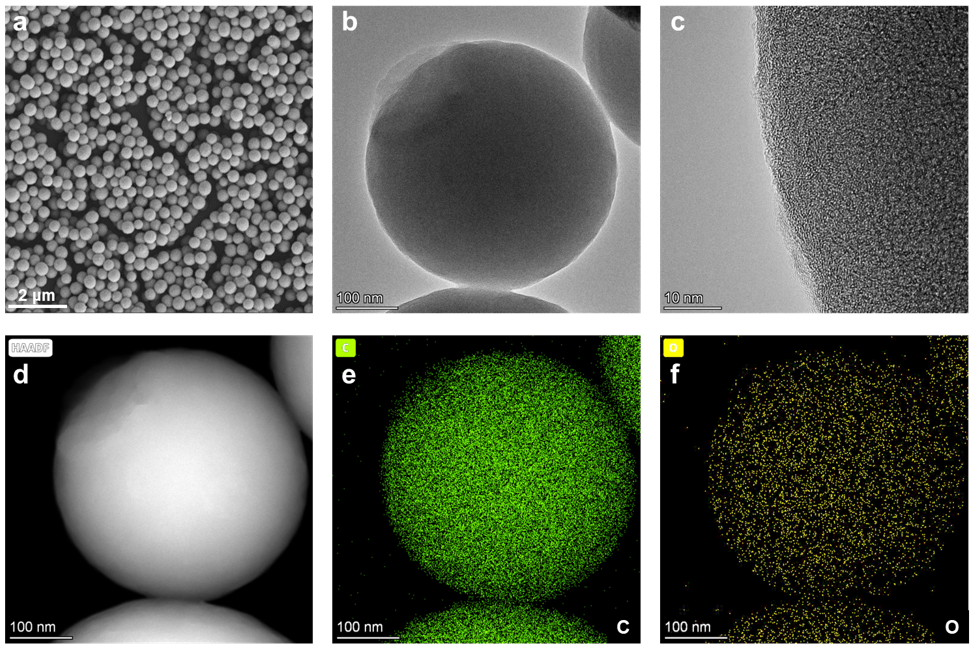


**Fig. S1.** (a) SEM, (b) TEM and (c) HRTEM images of CSs. (d-f) HAADF-STEM image and corresponding elemental mapping images of C and O.


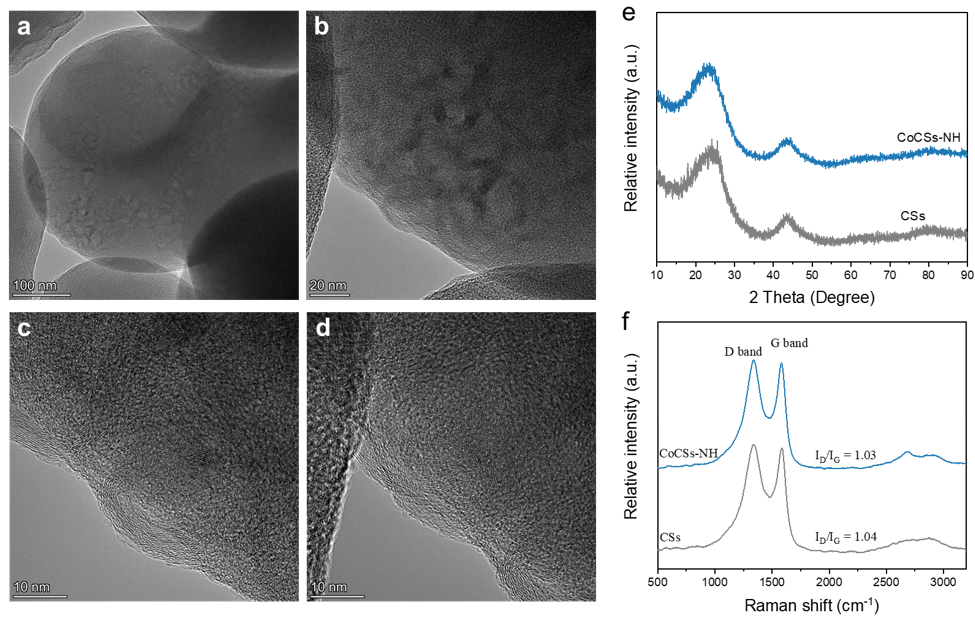


**Fig. S2.** (a, b) TEM and (c, d) HRTEM images of CoCSs-NH. (e) XRD patterns and (f) Raman spectra of CSs and CoCSs-NH.

**Note.** According to the ICP-MS analysis, the content of Co in Co^2+^-CSs was much higher than that without the hydrothermal treatment (Co^2+^-CSs-NH) (Table S1). Co^2+^-CSs-NH was also used instead of Co^2+^-CSs to elucidate the pivotal role of hydrothermal process for forming final hybrid structure. It can be observed that few graphitic domains were formed inside the CoCSs-NH, indicating that most Co ions can only be adsorbed on the surface of CSs and are difficult to travel into the CSs interior without the hydrothermal treatment. XRD, Raman and BET (Table S2) showed that no obvious change of structural properties was identified for CoCSs-NH when compared with CSs.

**
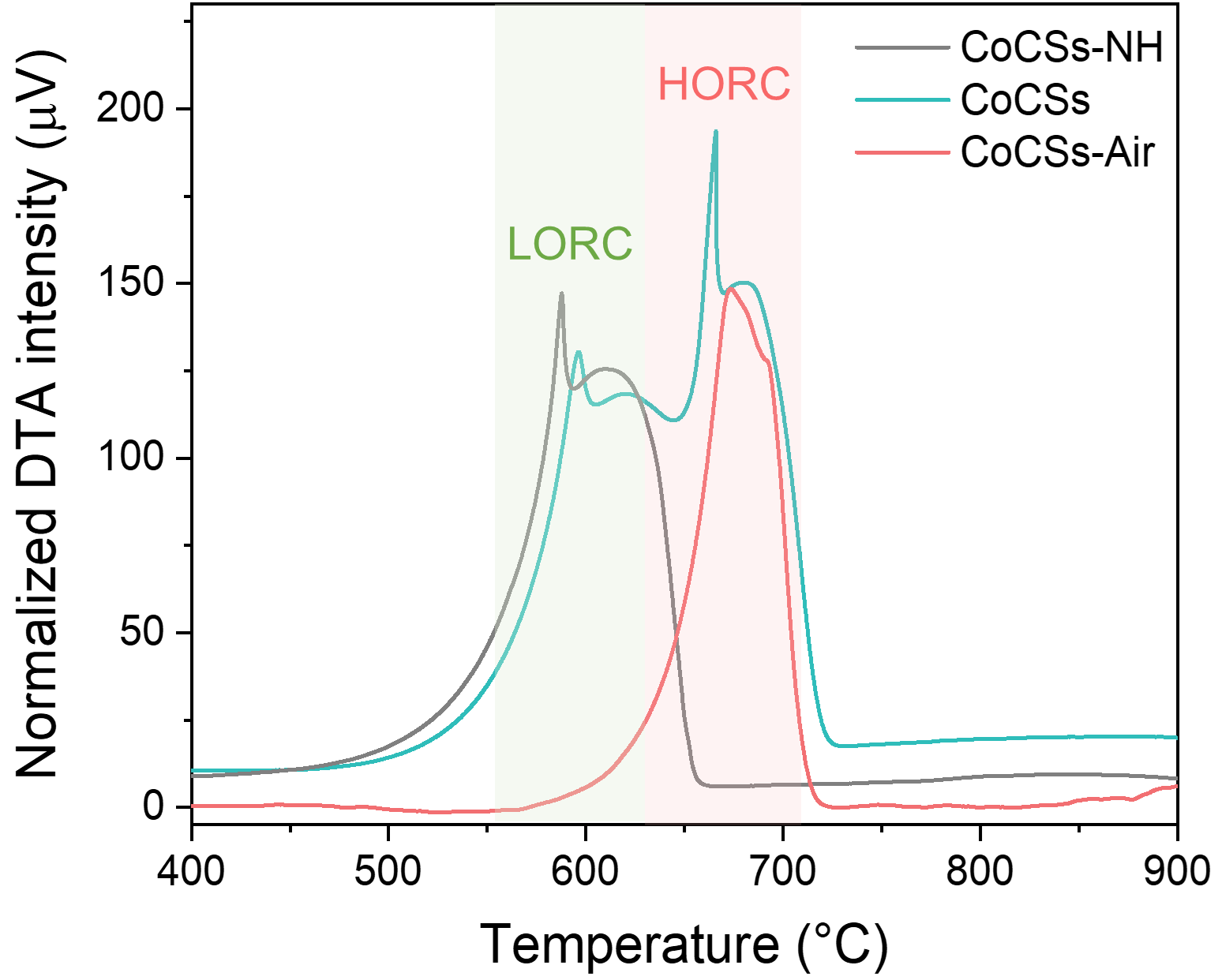
**

**Fig. S3.** Normalized DTA curves for CoCSs-NH, CoCSs and CoCSs-Air.


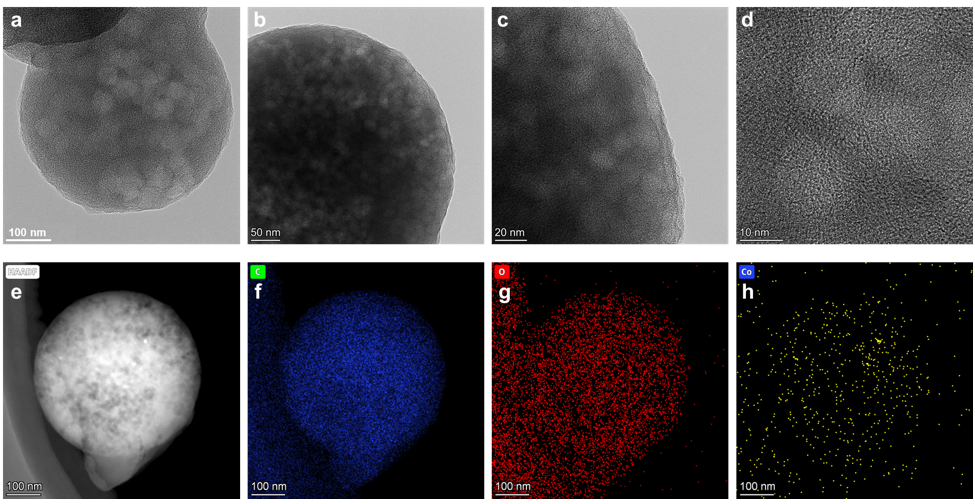


**Fig. S4.** (a-d) TEM images of CoCSs-800. (e-h) HAADF-STEM image and corresponding elemental mapping images of C, O and Co.


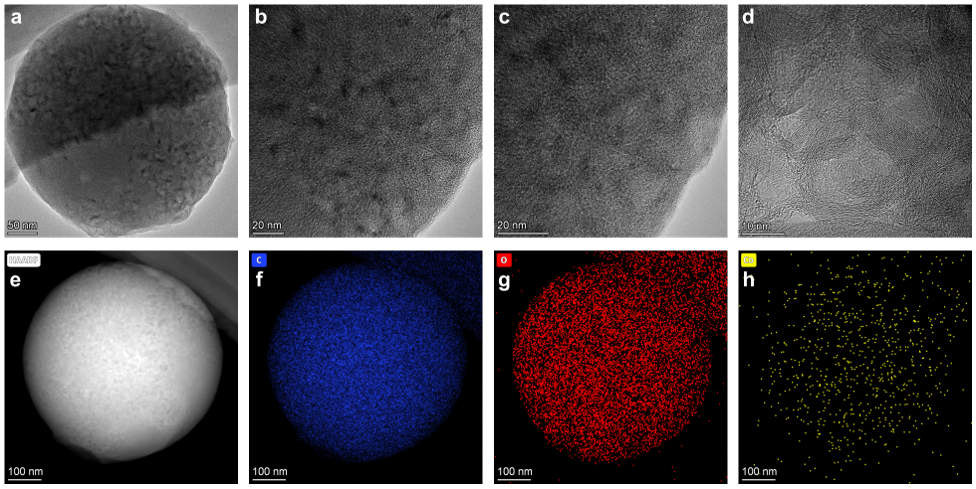


**Fig. S5.** (a-d) TEM images of CoCSs-900. (e-h) HAADF-STEM image and corresponding elemental mapping images of C, O and Co.


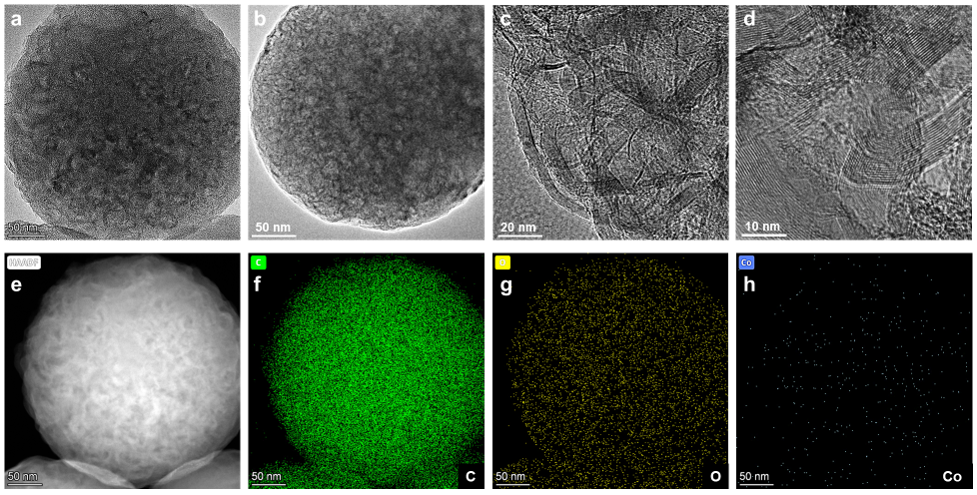


**Fig. S6.** (a, b) TEM and (c, d) HRTEM images of CoCSs-1000. (e-h) HAADF-STEM image and corresponding elemental mapping images of C, O and Co.

**
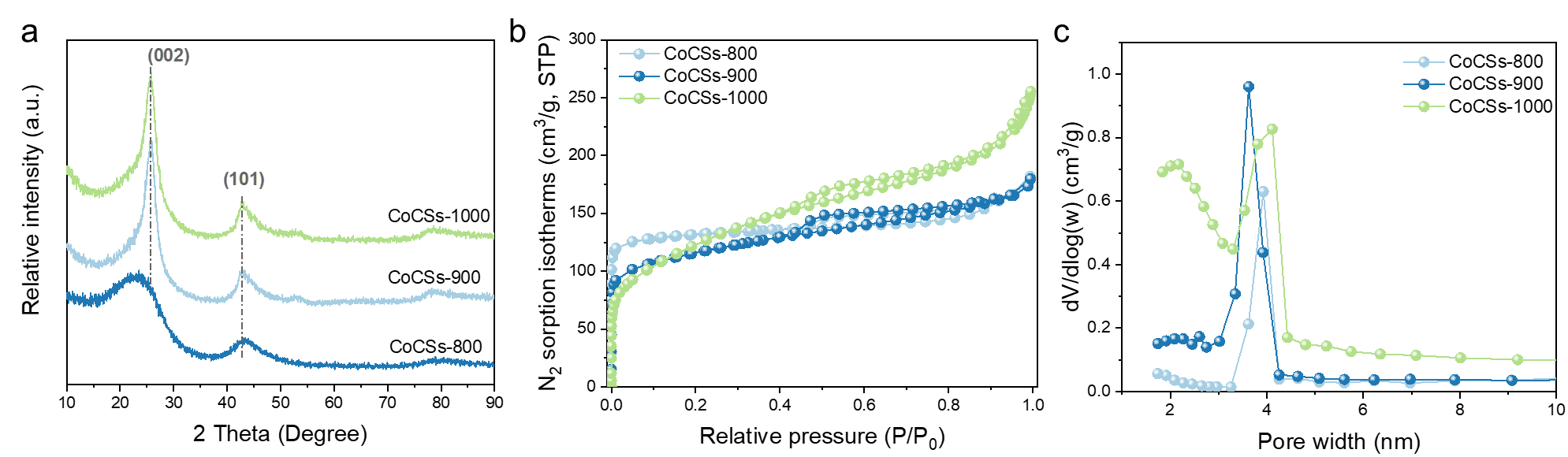
**

**Fig. S7.** (a) XRD patterns of CoCSs-800, CoCSs-900 and CoCSs-1000. (b) N_2_ sorption isotherms and (c) the corresponding Barrett-Joyner-Halenda (BJH) pore size distributions of CoCSs-800, CoCSs-900 and CoCSs-1000.

**Note.** The XRD pattern of CoCSs-800 displayed its amorphous carbon, while the typical (002) and (101) reflections corresponding to the graphite lattice planes were shown in CoCSs-900 and CoCSs-1000, demonstrating the well-developed graphitic carbon. The increment of mesopore volume in CoCSs samples as the temperature rises also indicated the formation of graphitized pore channels (Table S2).

**
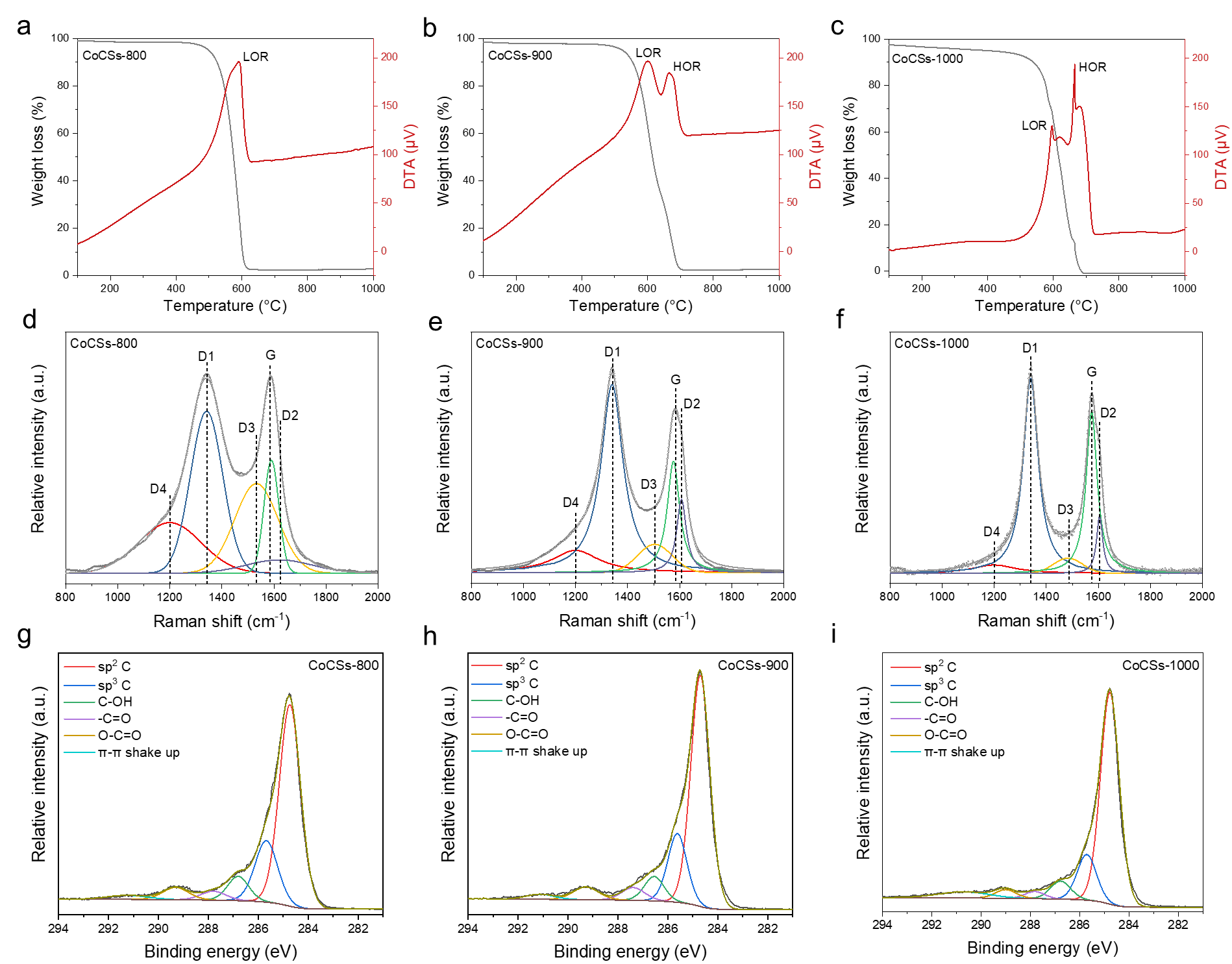
**

**Fig. S8.** TGA and the corresponding DTA curves for (a) CoCSs-800, (b) CoCSs-900 and (c) CoCSs-1000. Deconvoluted Raman spectra of (d) CoCSs-800, (e) CoCSs-900 and (f) CoCSs-1000. High-resolution C1s spectra of (g) CoCSs-800, (h) CoCSs-900 and (i) CoCSs-1000.

**Note.** According to the DTA results, CoCSs-800 exhibited a single exothermal peak centered at 600 °C due to the oxidation of amorphous carbon with low oxidation-resistance (LOR). An additional exothermal peak centered at 670 °C appeared in CoCSs-900 and CoCSs-1000, which could be ascribed to the combustion of graphitic carbon with high oxidation-resistance (HOR). The fitted results of Raman spectroscopy revealed that the content of amorphous carbon (D3 band) decreased with increasing temperature. As shown in C1s spectra, the CoCSs-1000 possessed the highest sp^2^/sp^3^ carbon ratio (4.5) when compared with CoCSs-800 (3.1) and CoCSs-900 (3.5) (Table S3).


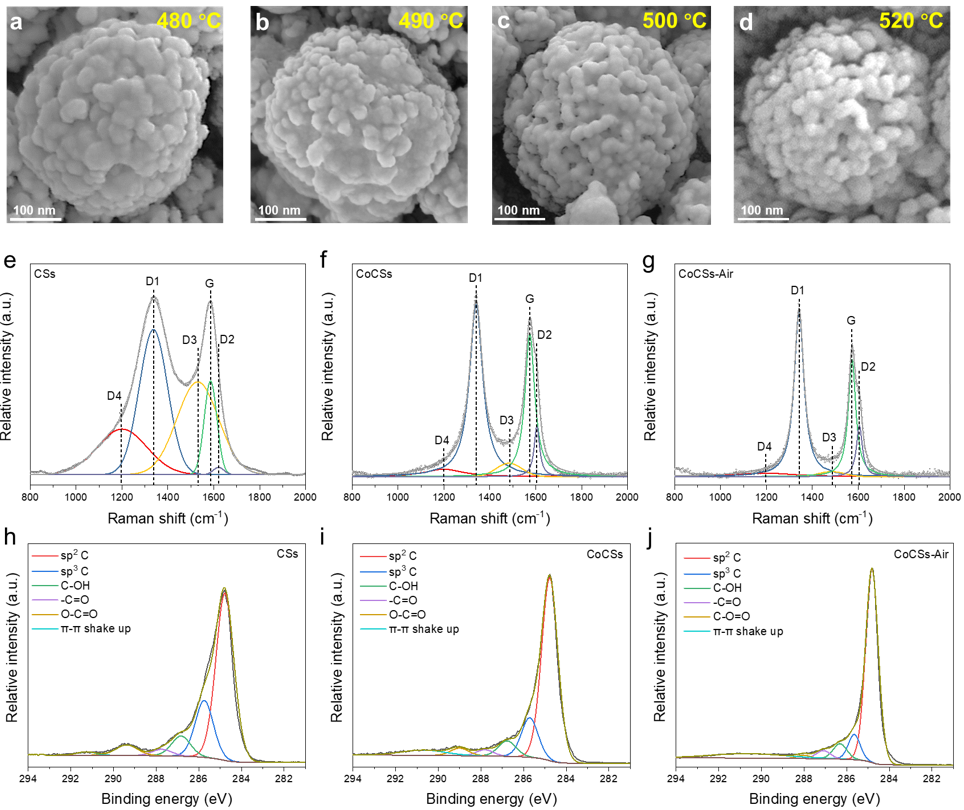


**Fig. S9.** SEM images of (a) CoCSs-Air-480, (b) CoCSs-Air-490, (c) CoCSs-Air-500 and (d) CoCSs-Air-520. Deconvoluted Raman spectra of (e) CSs, (f) CoCSs and (g) CoCSs-Air. High-resolution C1s spectra of (h) CSs, (i) CoCSs and (j) CoCSs-Air.

**Note.** The fitted results of Raman spectroscopy demonstrated that CoCSs-Air possessed the lowest ratio of D3 band (representing amorphous carbon) compared to CoCSs and CSs, suggesting the removal of most amorphous carbon. Moreover, CoCSs-Air possessed the highest sp^2^/sp^3^ carbon ratio among the three samples from the high-resolution C1s surveys (Table S3).

**
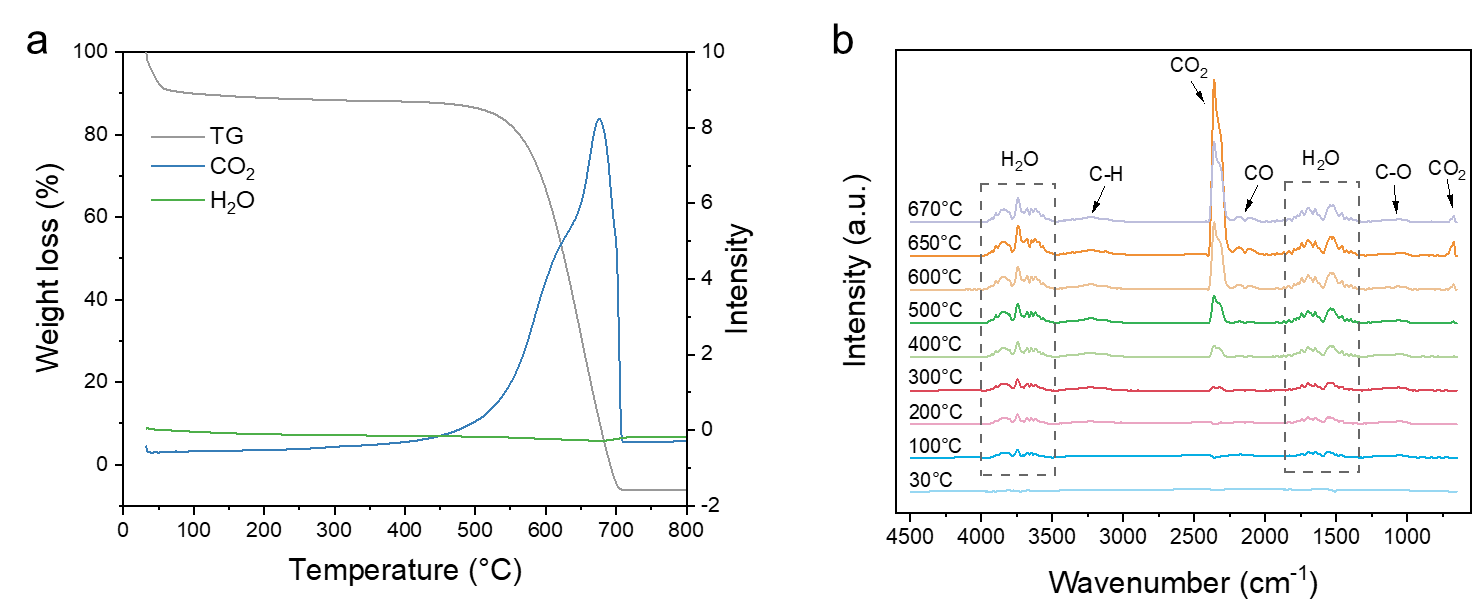
**

**Fig. S10.** (a, b) TG-FTIR spectra of pyrolysis products of CoCSs at different temperatures under an O_2_ gas flow.


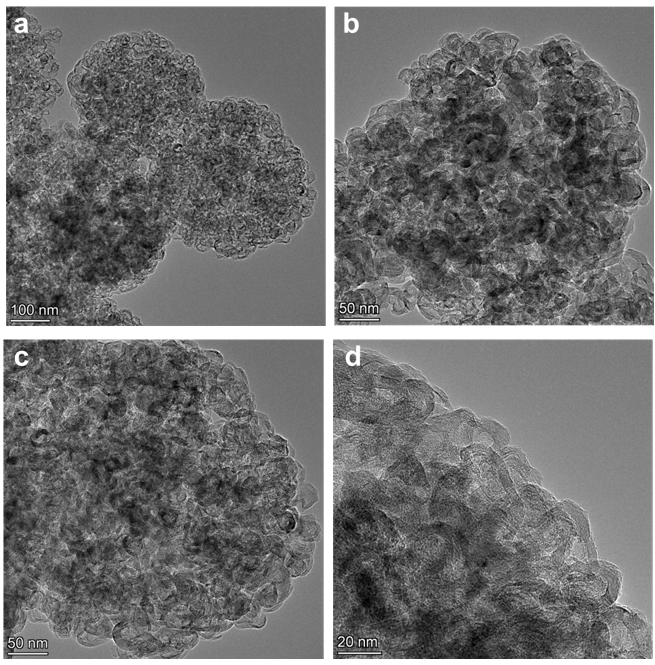


**Fig. S11.** (a-d) TEM images of CoCSs-Air.


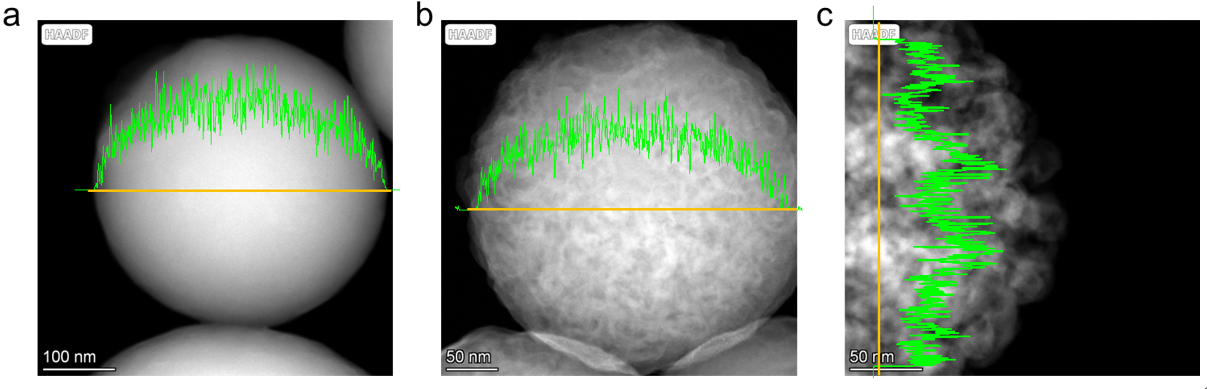


**Fig. S12.** TEM-EDX line scans of C element in (a) CSs, (b) CoCSs and (c) CoCSs-Air.

**
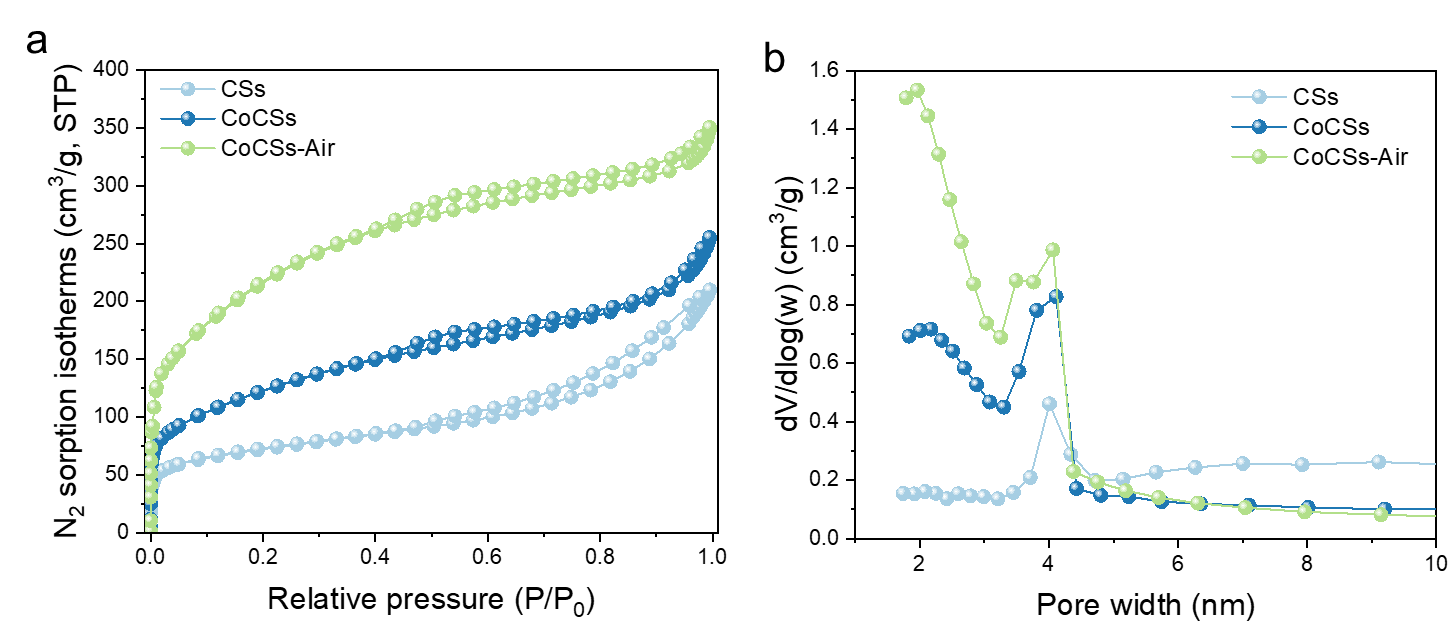
**

**Fig. S13.** (a) N_2_ sorption isotherms and (b) the corresponding Barrett-Joyner-Halenda (BJH) pore size distributions of CSs, CoCSs and CoCSs-Air.


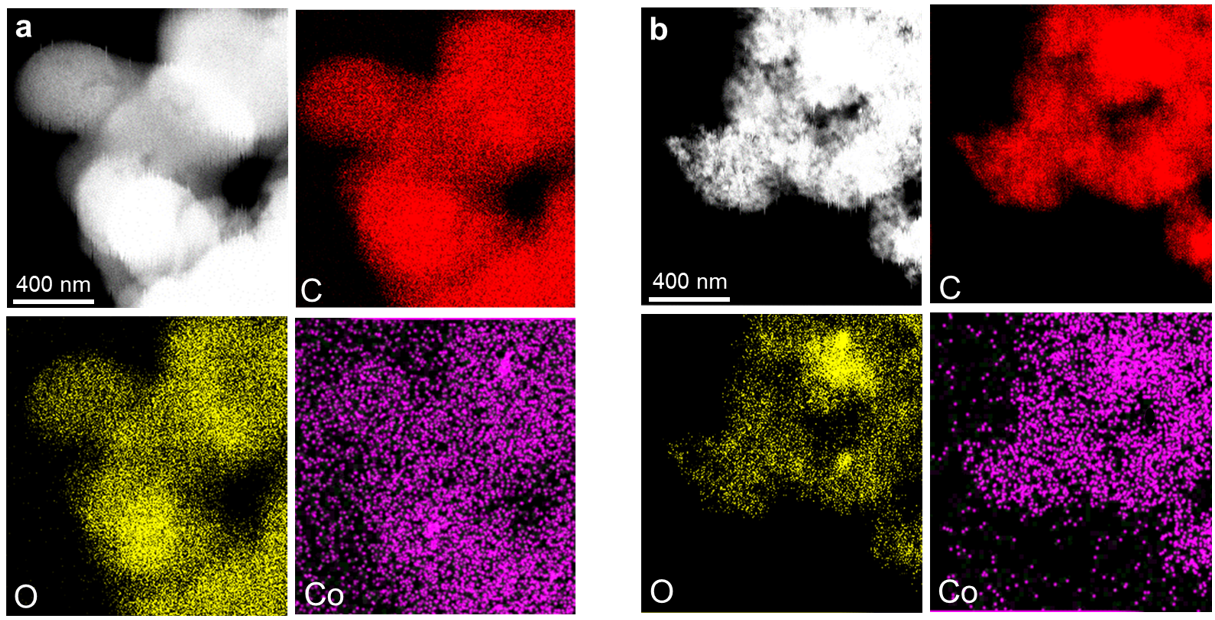


**Fig. S14.** HAADF-STEM and the corresponding elemental mapping images of (a) CoCSs and (b) CoCSs-Air.

**
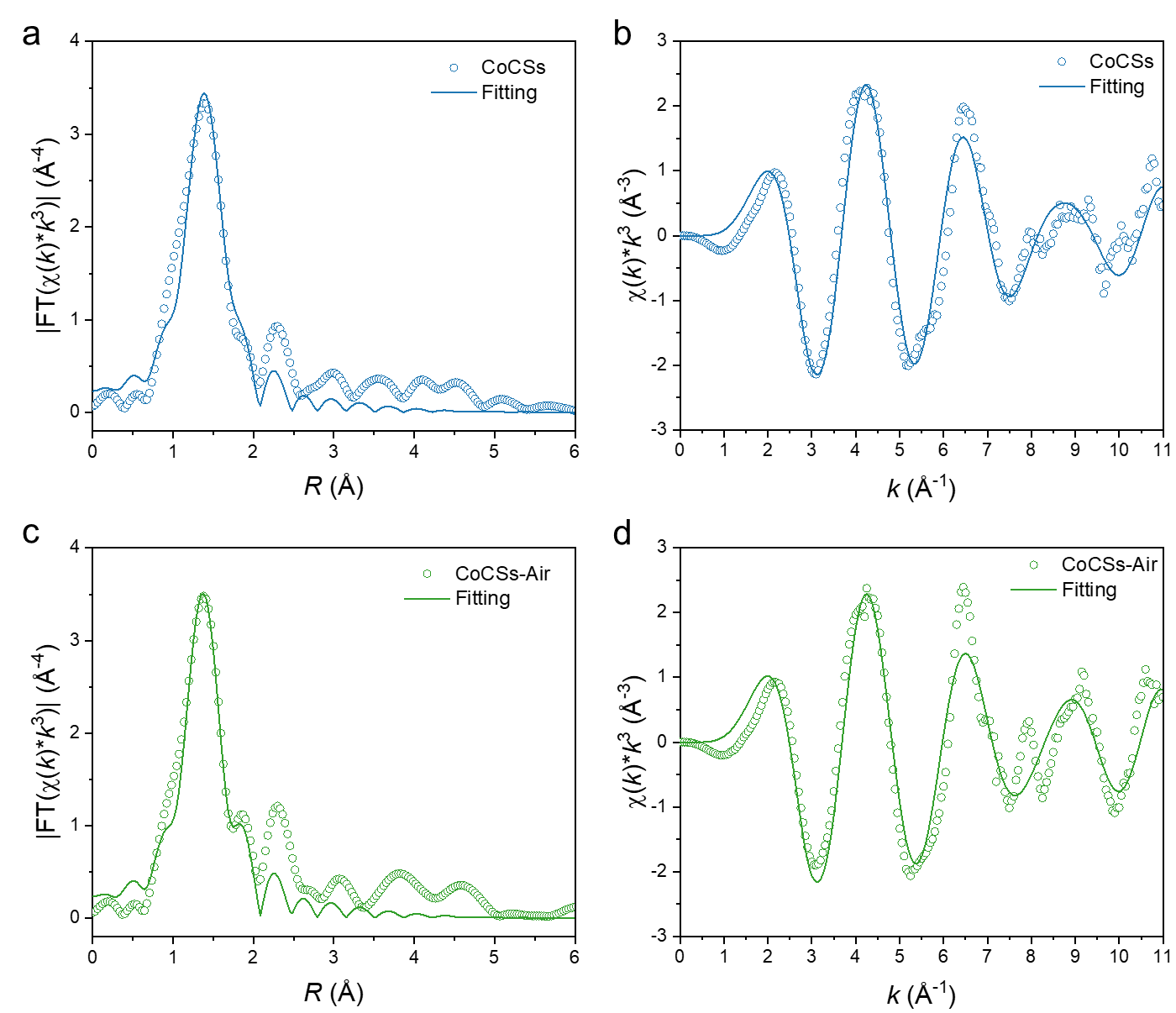
**

**Fig. S15.** The EXAFS fitted curves of CoCSs (a) at R space and (b) at k space. The EXAFS fitted curves of CoCSs-Air (c) at R space and (d) at k space.

**
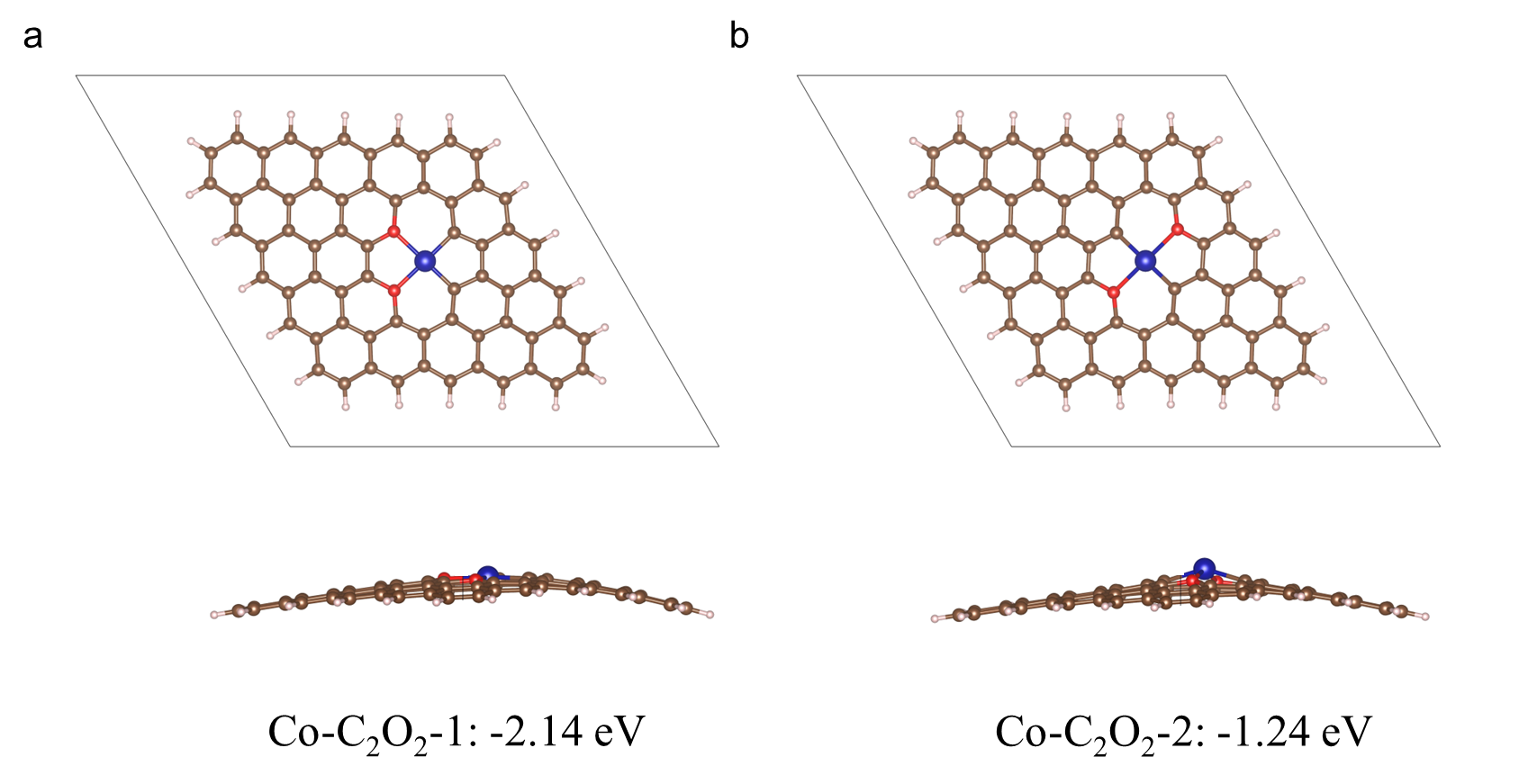
**

**Fig. S16.** The formation energies of (a) Co-C_2_O_2_-1 model and (b) Co-C_2_O_2_-2 model. The blue, red and brown spheres represent cobalt, oxygen and carbon atoms, respectively.

**Note.** The formation energy for Co-C_2_O_2_-1 was lower than that of Co-C_2_O_2_-2, indicating that the isolated Co atoms would adopt a favorable structure to form asymmetric Co-C_2_O_2_-1 configuration.

**
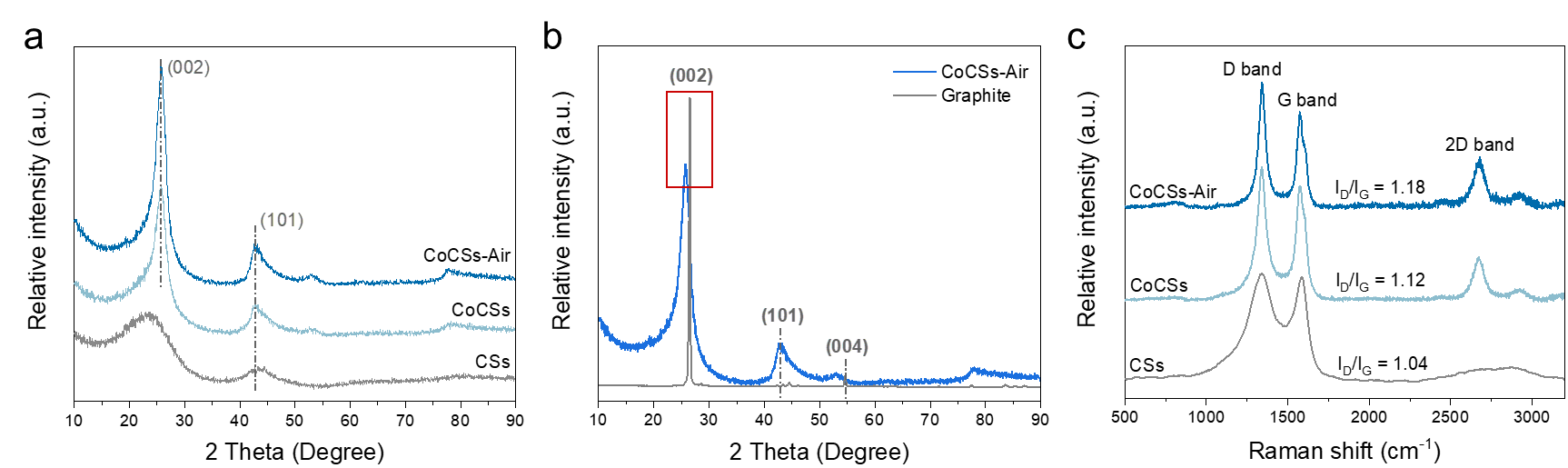
**

**Fig. S17.** (a) XRD patterns of CSs, CoCSs and CoCSs-Air. (b) XRD patterns of CoCSs-Air and graphite. (c) Raman spectra of CSs, CoCSs and CoCSs-Air.

**Note.** As revealed by the XRD patterns, the (002) reflections of CoCSs and CoCSs-Air exhibited broad asymmetric profiles, demonstrating that the graphitized parts in these materials possessed a high degree of disorder. Compared to graphite, the negative shift of (002) peak indicated the expansion of the lattice spacing, which agrees well with the HRTEM results (Fig. S18). Raman spectra also illustrated the defect-rich graphitic carbon structure, in which the I_D_/I_G_ value of CoCSs-Air (1.18) was higher than that of CoCSs (1.12) and CSs (1.04). Furthermore, the sharp 2D peak at around 2732 cm^-1^ revealed the formation of few-layered graphene.


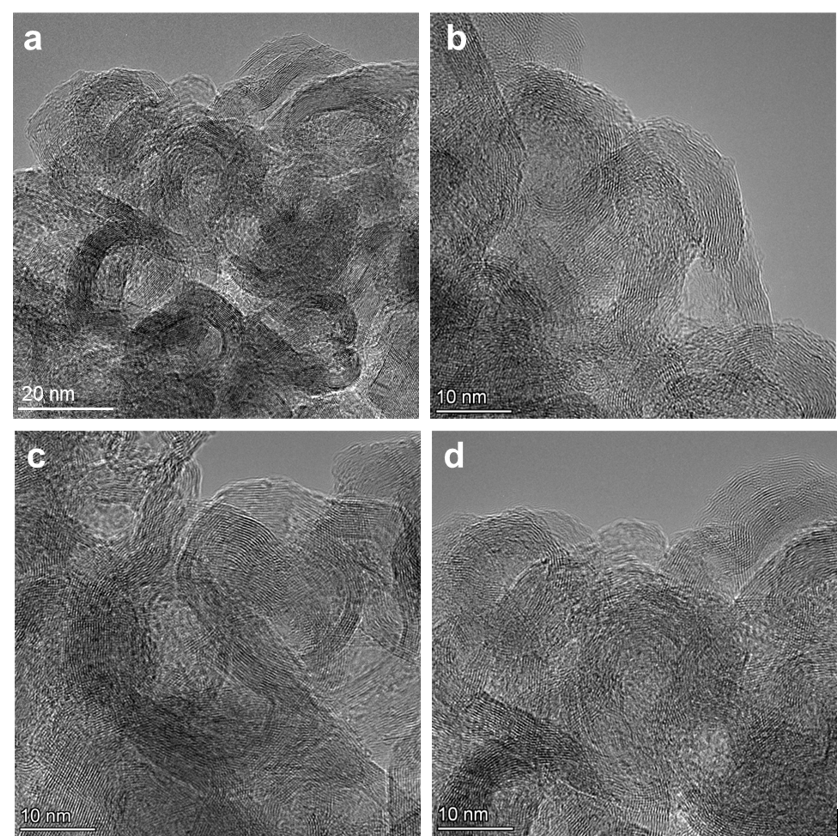


**Fig. S18.** (a-d) HRTEM images of CoCSs-Air.

**Note.** The interplanar spacing of graphene layers was calculated to be 0.36 nm, larger than that of graphite (0.34 nm). We can clearly observe the buckling, splitting and merging of graphene layers in HRTEM images, also evidencing the highly disordered graphitic phase.


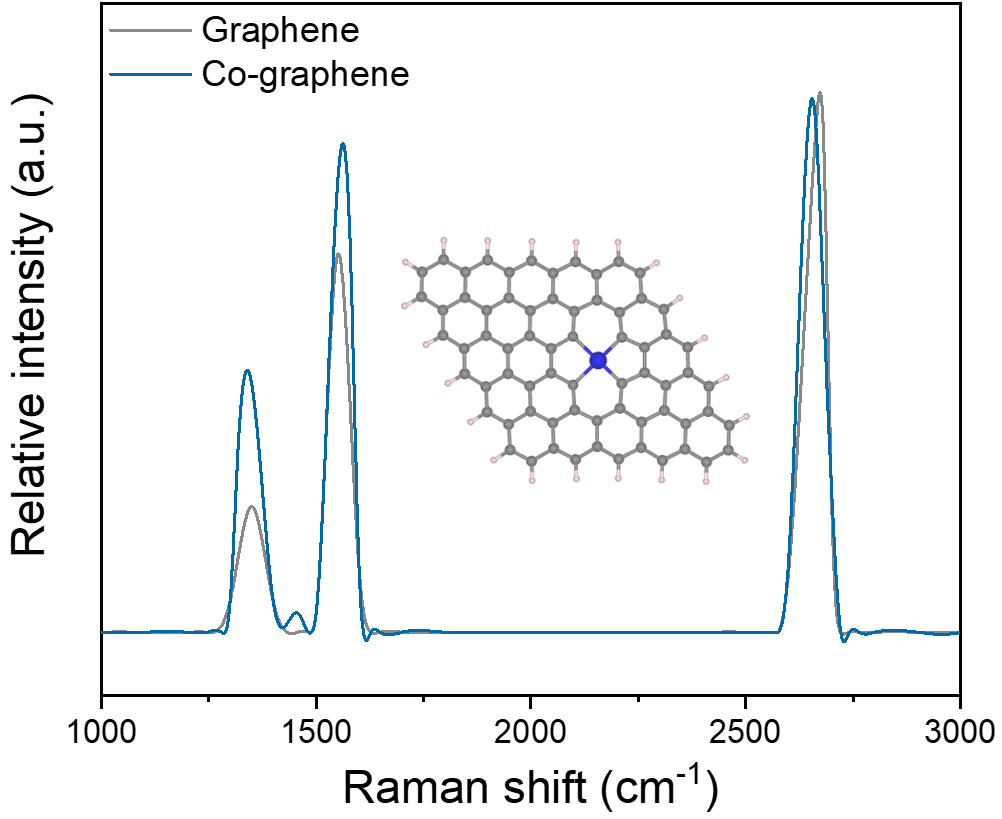


**Fig. S19.** Simulated Raman spectra of graphene and Co-doped graphene.


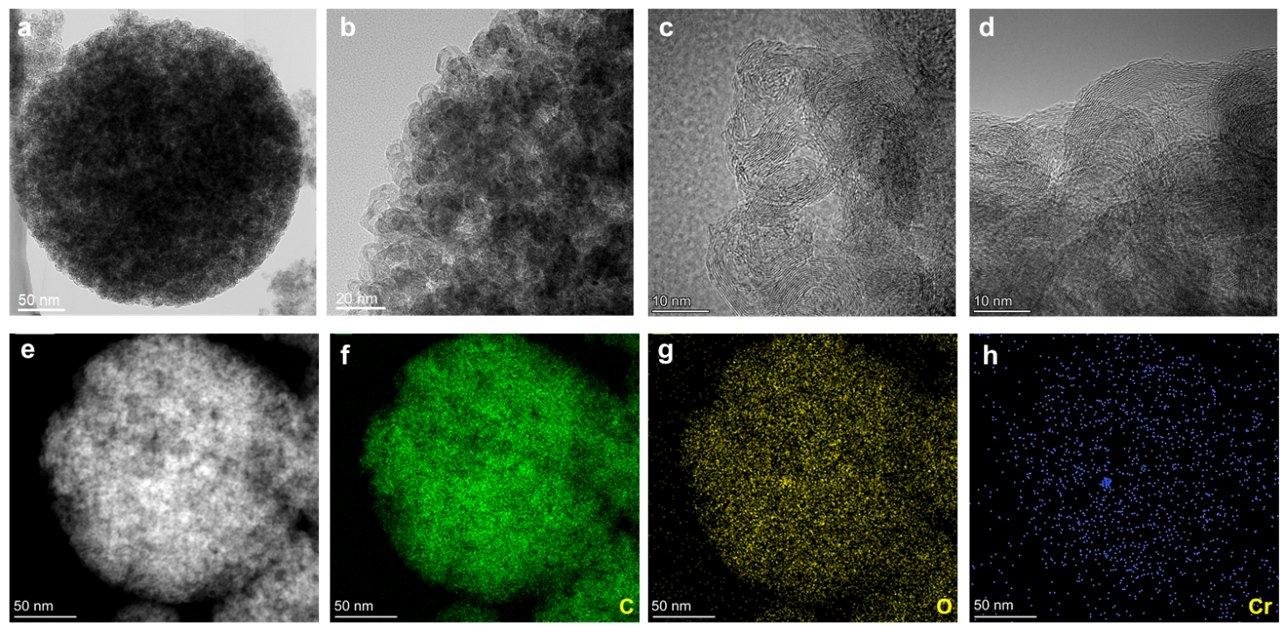


**Fig. S20.** (a-d) TEM images of CrCSs-Air. (e-h) HAADF-STEM image and corresponding elemental mapping images of C, O and Cr.


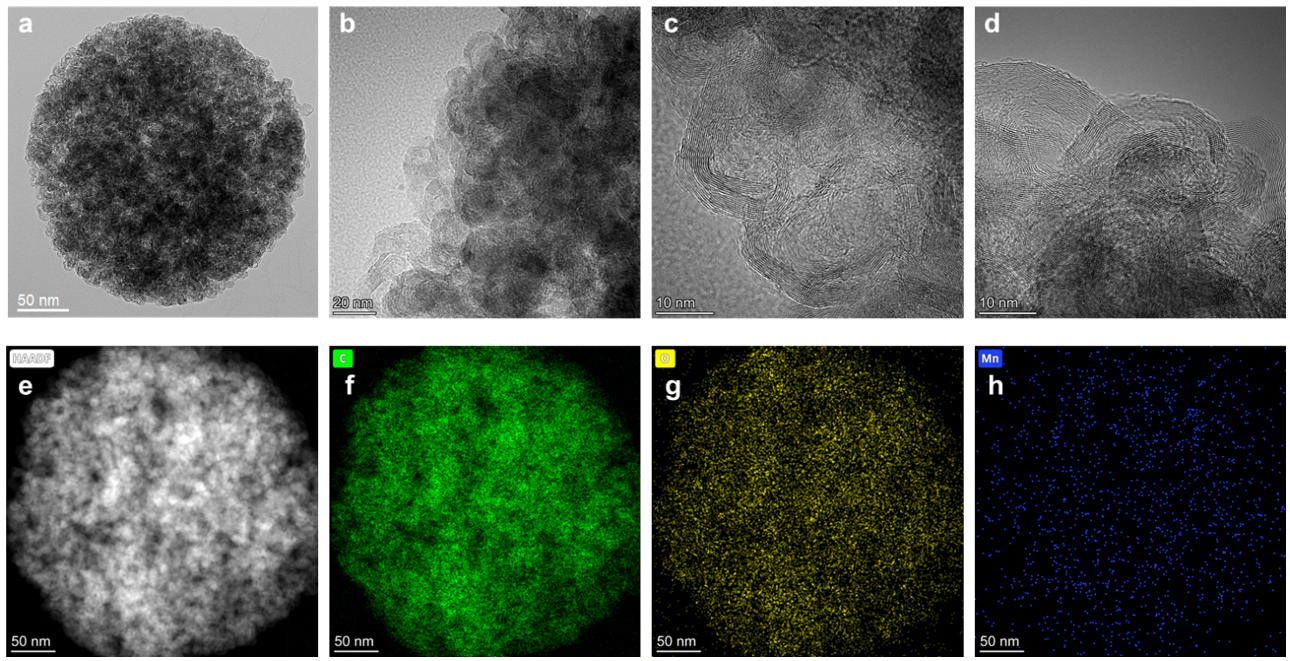


**Fig. S21.** (a-d) TEM images of MnCSs-Air. (e-h) HAADF-STEM image and corresponding elemental mapping images of C, O and Mn.


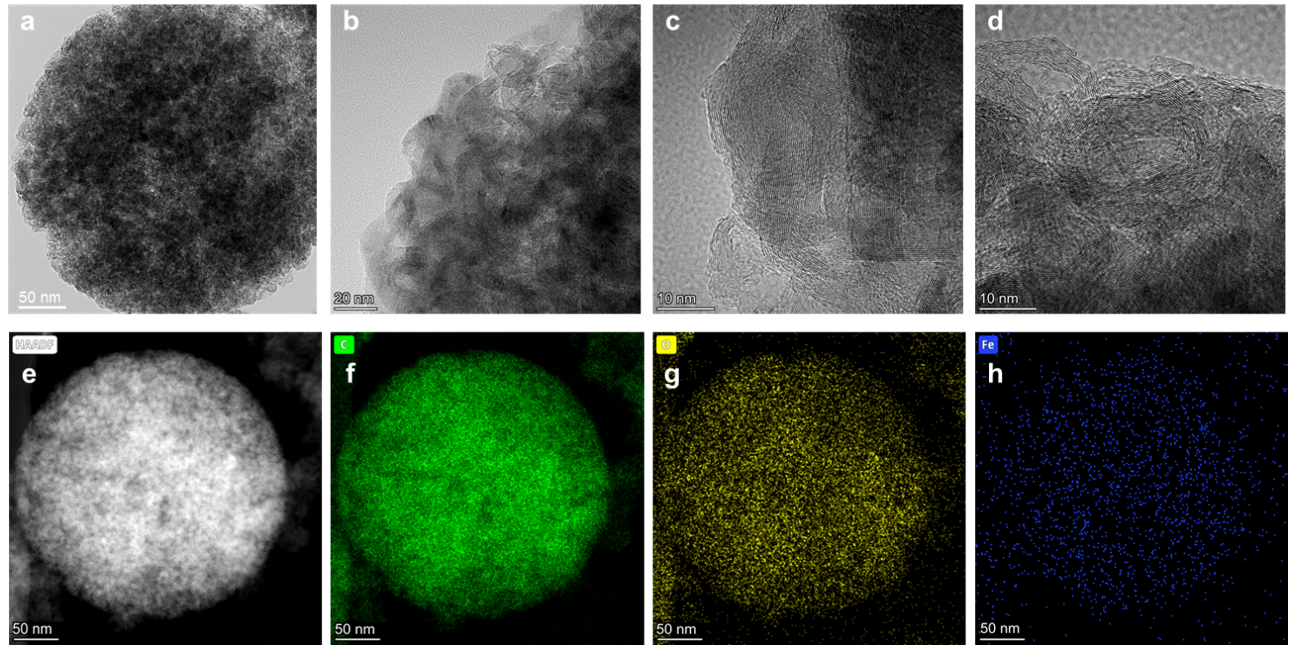


**Fig. S22.** (a-d) TEM images of FeCSs-Air. (e-h) HAADF-STEM image and corresponding elemental mapping images of C, O and Fe.


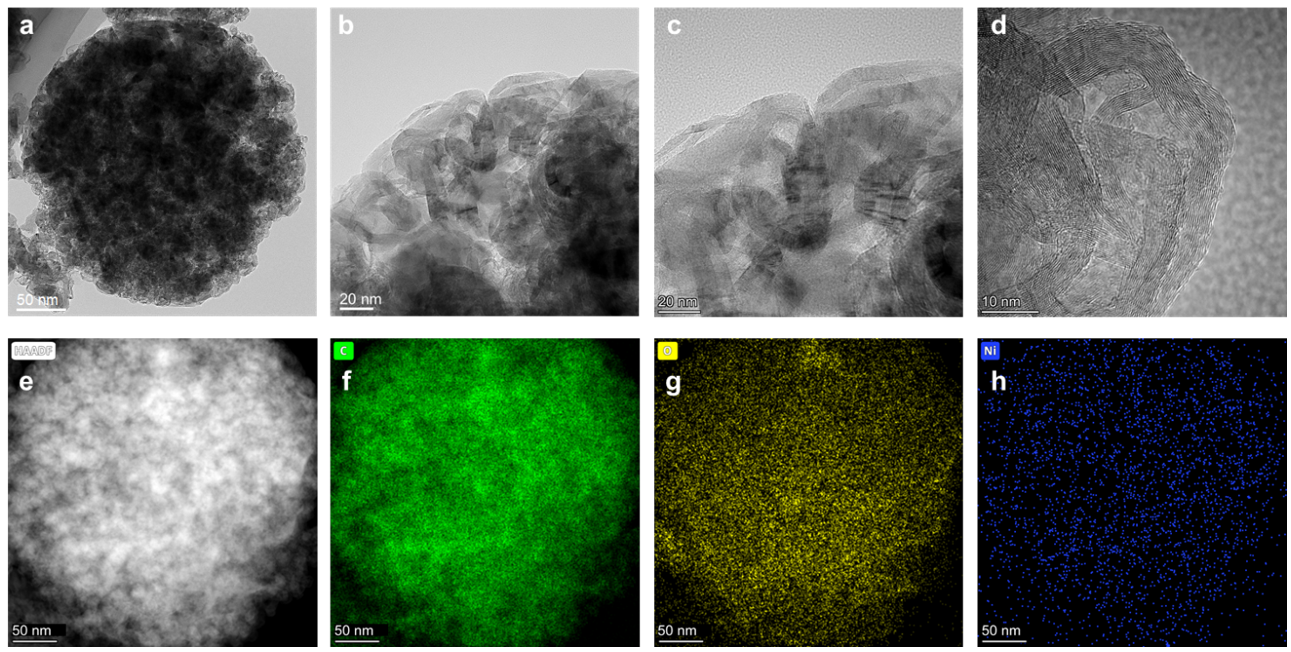


**Fig. S23.** (a-d) TEM images of NiCSs-Air. (e-h) HAADF-STEM image and corresponding elemental mapping images of C, O and Ni.


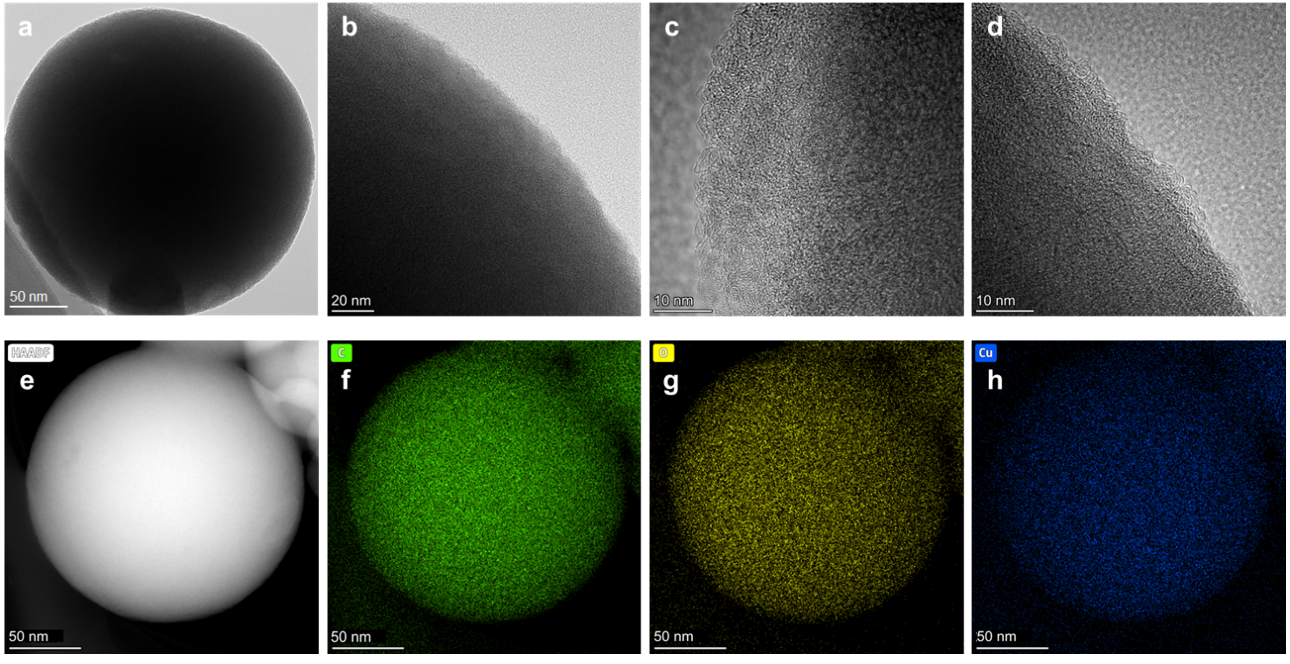


**Fig. S24.** (a-d) TEM images of CuCSs. (e-h) HAADF-STEM image and corresponding elemental mapping images of C, O and Cu.

**
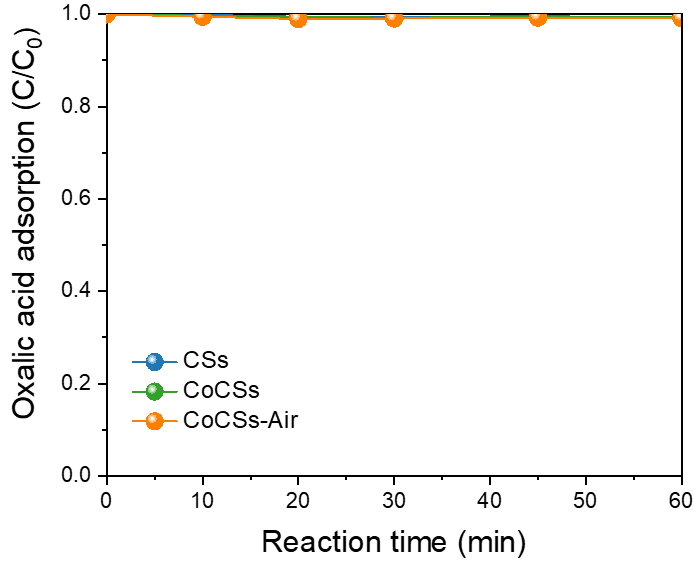
**

**Fig. S25.** Adsorption of oxalic acid on CSs, CoCSs and CoCSs-Air.


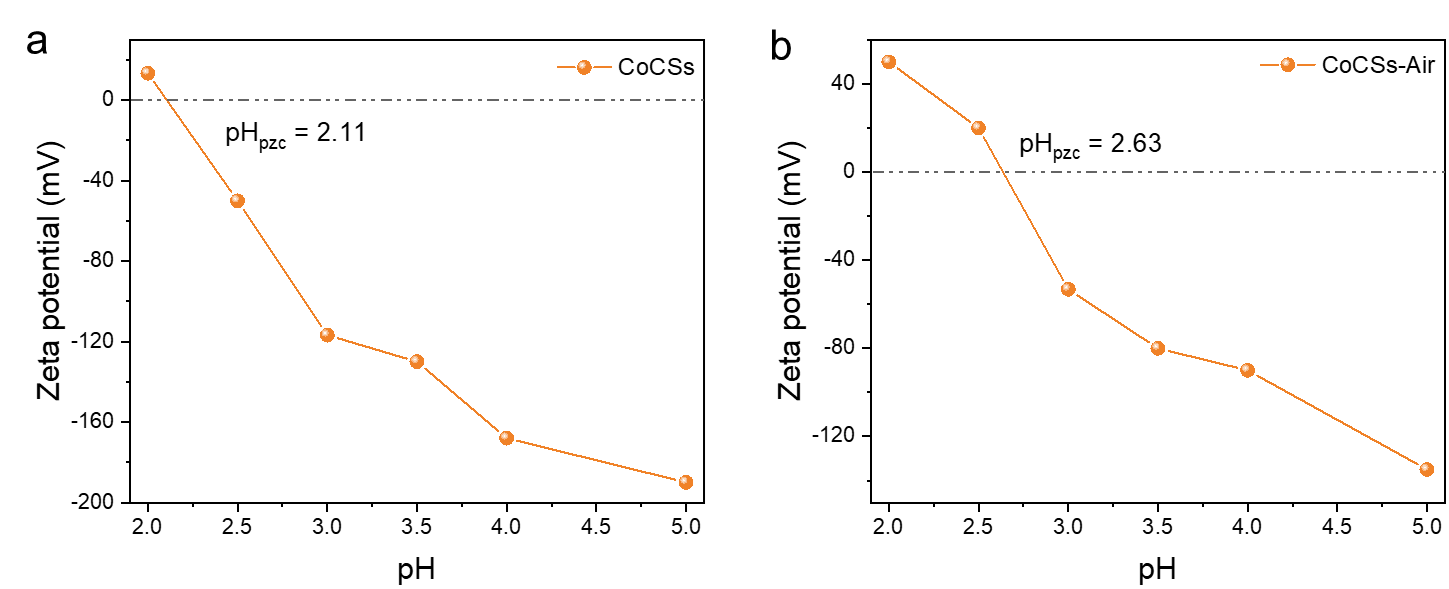


**Fig. S26.** Zeta potentials of (a) CoCSs and (b) CoCSs-Air under different pH.


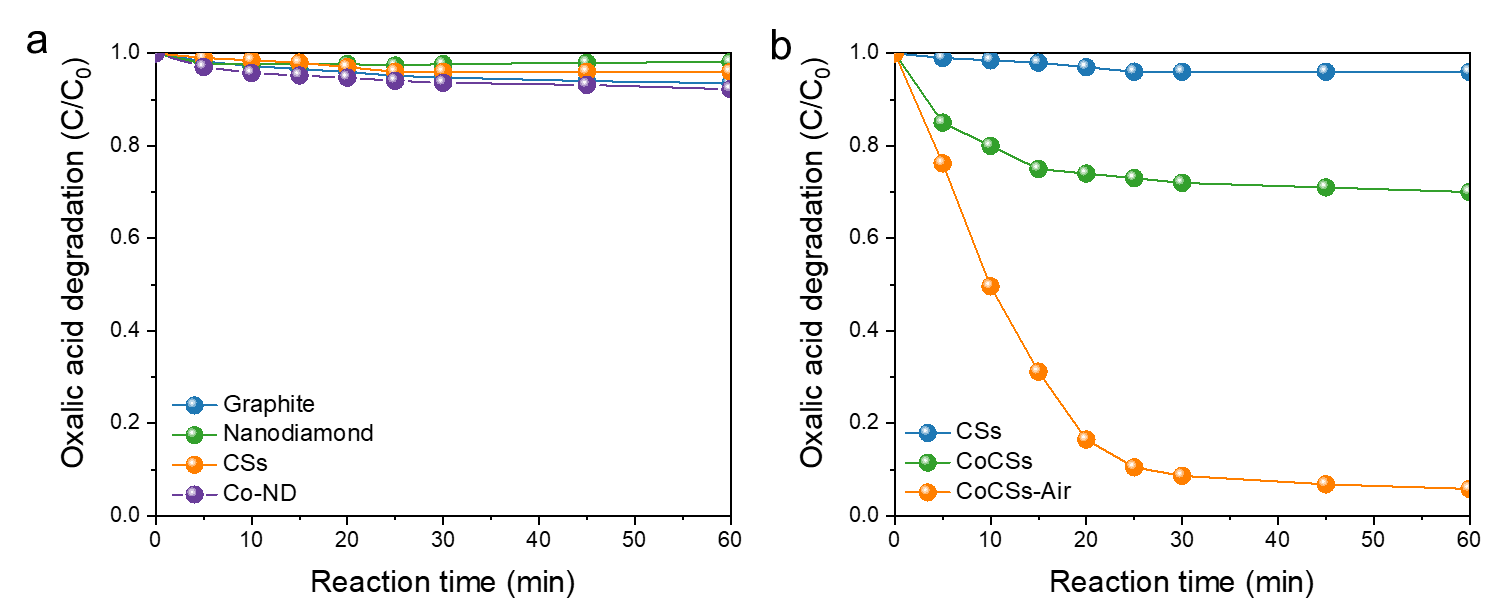


**Fig. S27.** (a) Degradation of OA over CSs, graphite, nanodiamond and Co-graphite. (b) Degradation of OA over CSs, CoCSs and CoCSs-Air. Reaction conditions: catalyst loading: 0.01 g L^-1^; [OA]_0_: 150 mg L^-1^; ozone flow rate: 100 mL min^-1^; ozone concentration: 15 mg L^-1^; temperature: 25 °C; initial pH: 3.0.

**
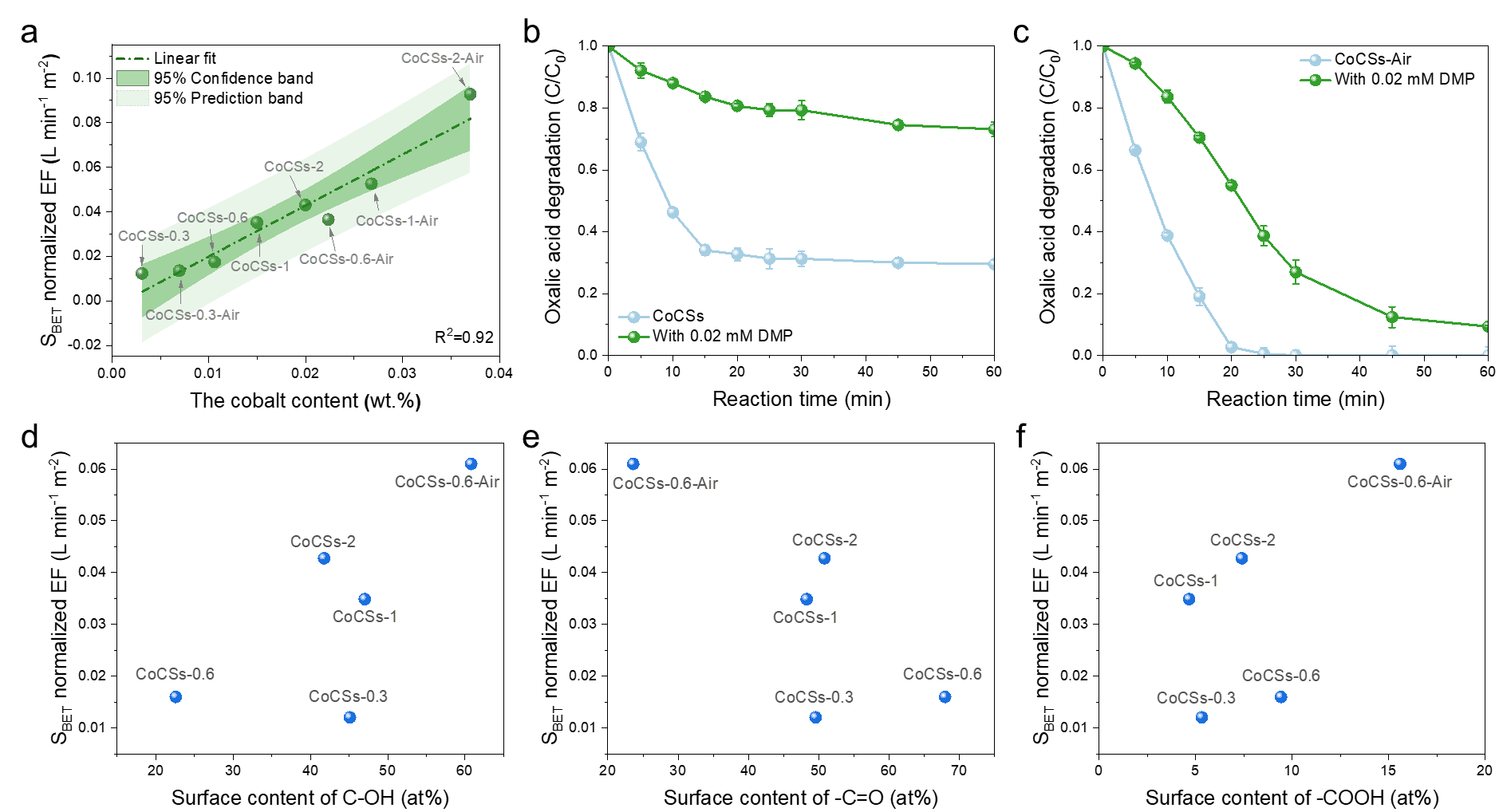
**

**Fig. S28.** (a) Correlation of the S_BET_ normalized EF values to Co contents in catalysts. Effect of DMP on the catalytic ozonation efficiency of (b) CoCSs and (c) CoCSs-Air. (d) Correlation of the S_BET_ normalized EF values to (d) surface C-OH contents, (e) surface -C=O contents and (f) surface -COOH contents in catalysts.

**Note.** CoCSs and CoCSs-Air catalysts with different Co amounts were designed to investigate the relationship between the catalytic activities and Co contents. The significant linear correlation between the S_BET_ normalized EF values and the corresponding cobalt contents in catalysts indicated that Co single atoms on the graphitic carbon were the active sites. We also used 2,3-dimercapto-1-propanol (DMP) as the metal chelating agent to poison the Co sites. The declined activity with DMP addition further confirmed the role of Co atoms as the active sites. Additionally, the influences of surface oxygen groups, including -OH, -C=O and -COOH, were excluded because the S_BET_ normalized EF values were not significantly correlated with the contents of oxygen groups (Table S4).

**
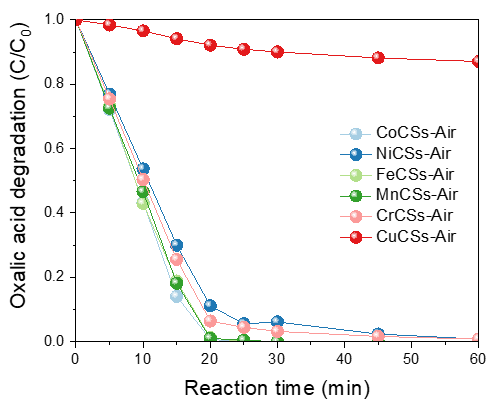
**

**Fig. S29.** Degradation of OA over TMCSs-Air (TMs = Co, Cr, Mn, Fe, Ni, Cu). Reaction conditions: catalyst loading: 0.05 g L^-1^; [OA]_0_: 150 mg L^-1^; ozone flow rate: 100 mL min^-1^; ozone concentration: 15 mg L^-1^; temperature: 25 °C; initial pH: 3.0.


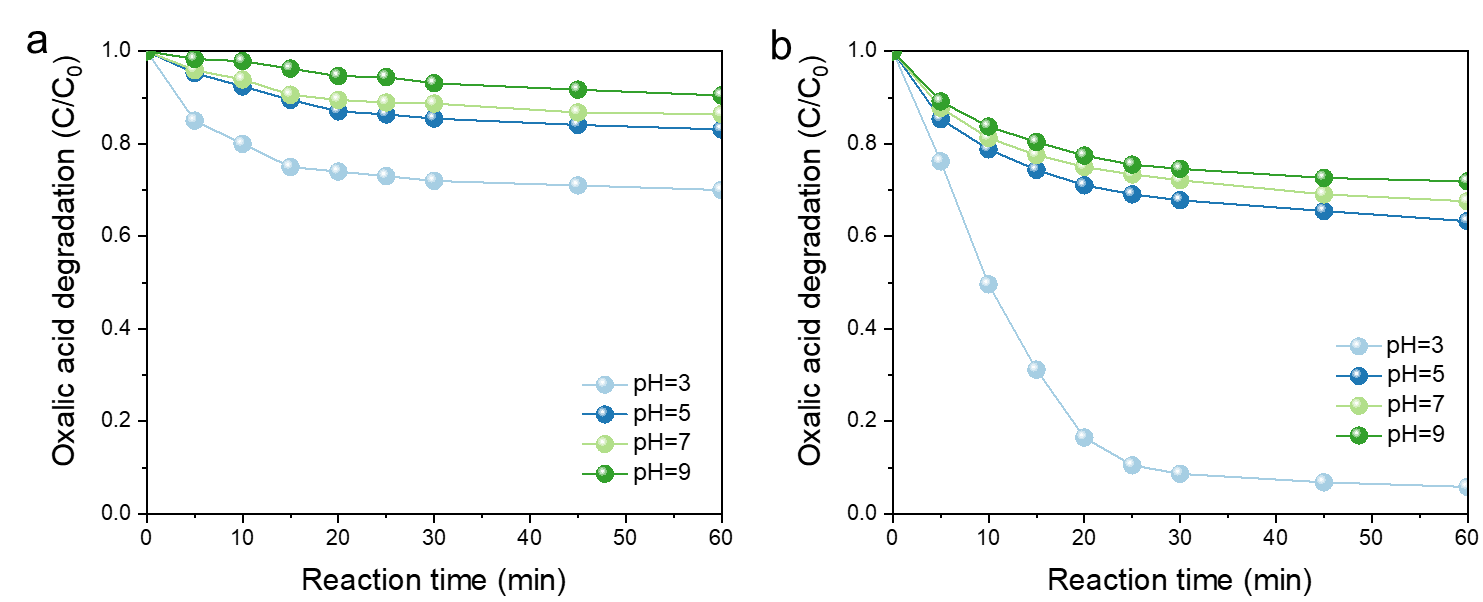


**Fig. S30.** Effect of pH on the catalytic activity of (a) CoCSs and (b) CoCSs-Air. Reaction conditions: catalyst loading: 0.01 g L^-1^; [OA]_0_: 150 mg L^-1^; ozone flow rate: 100 mL min^-1^; ozone concentration: 15 mg L^-1^; temperature: 25 °C.


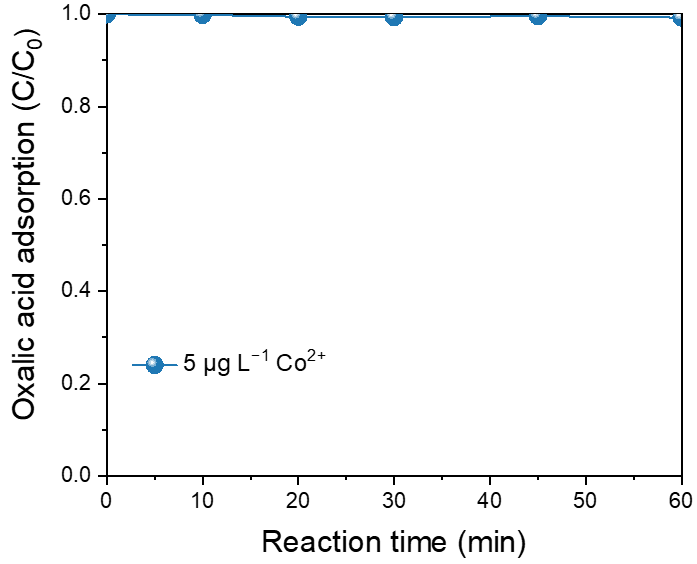


**Fig. S31.** Catalytic activity of homogeneous ozonation. Reaction conditions: [OA]_0_: 150 mg L^-1^; [Co^2+^]_0_: 5 μg L^-1^; ozone flow rate: 100 mL min^-1^; ozone concentration: 15 mg L^-1^; temperature: 25 °C.

**
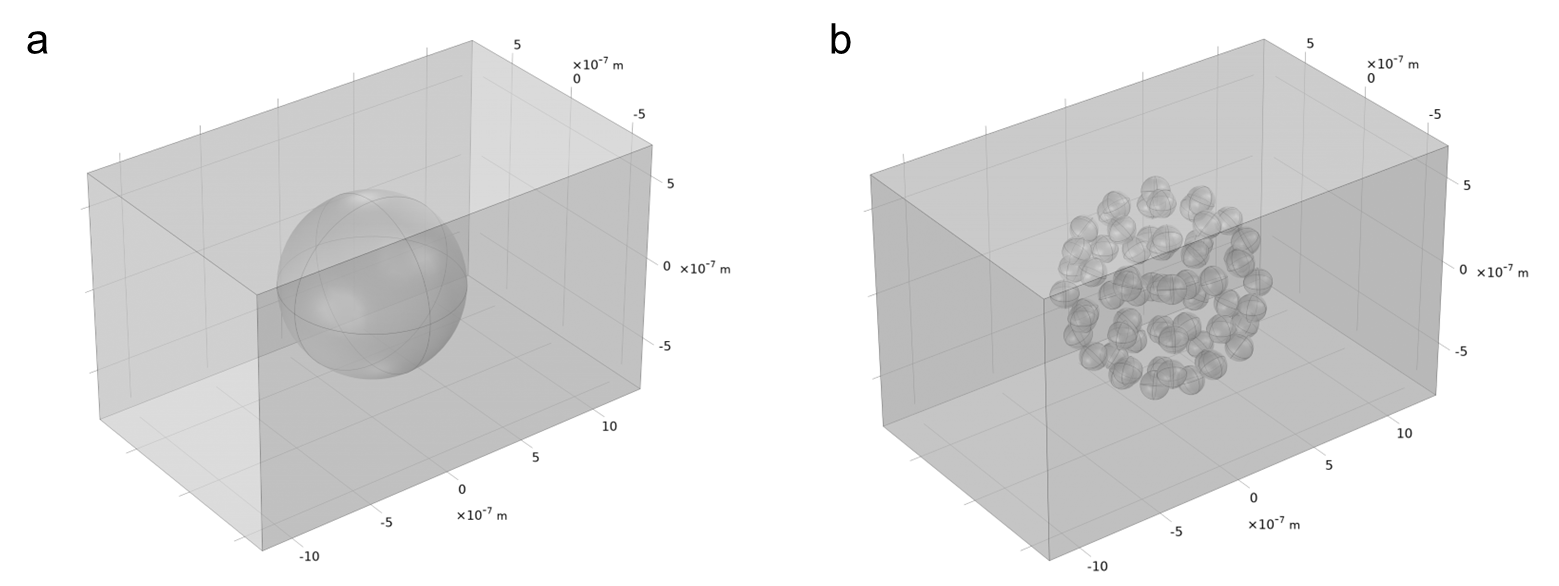
**

**Fig. S32.** The FEM models for the structures of individual particles in (a) CoCSs (Model 1) and (b) CoCSs-Air (Model 2).

**Note.** We implemented finite element method (FEM) simulations to investigate the impact of the catalysts’ geometric configurations on the mass transfer properties in the catalytic ozonation reactions. Based on the SEM and TEM characterizations, two models were correspondingly established for the individual particles with solid (Model 1) and open-framework (Model 2) nanostructure in the CoCSs and CoCSs-Air materials, respectively.


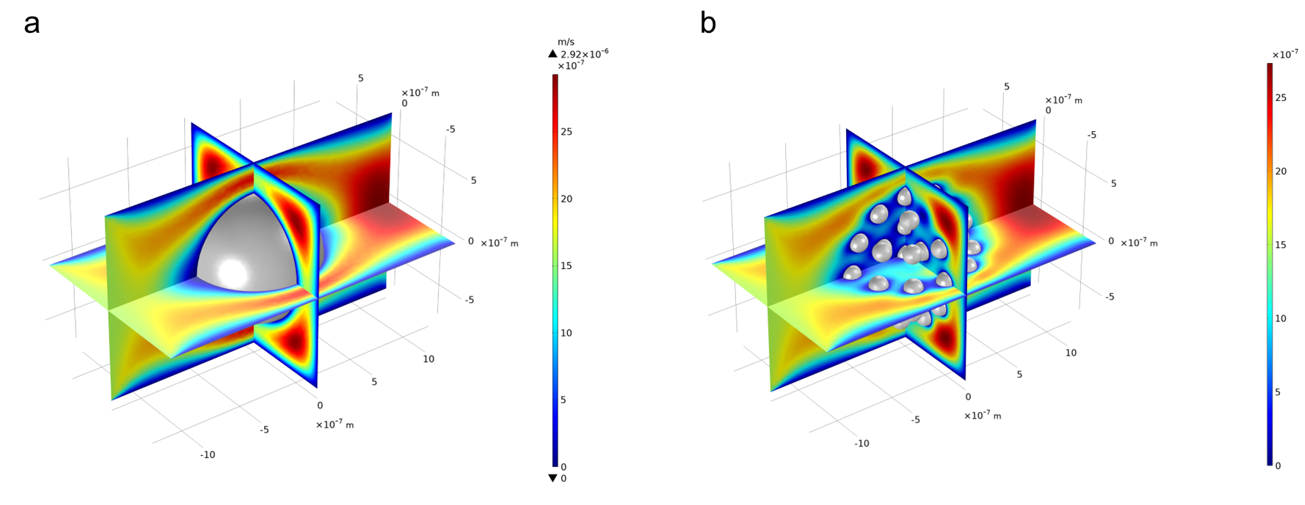


**Fig. S33.** The velocity field in (a) Model 1 and (b) Model 2.

**Note.** As shown from the simulated velocity fields, the fluidic flow velocity in the intraparticle space of Model 2 showed a remarkable enhancement as compared to Model 1. Therefore, we hypothesized that the opened mesoporous channels in CoCSs-Air were beneficial to facilitate the diffusion and conversion of O_3_.


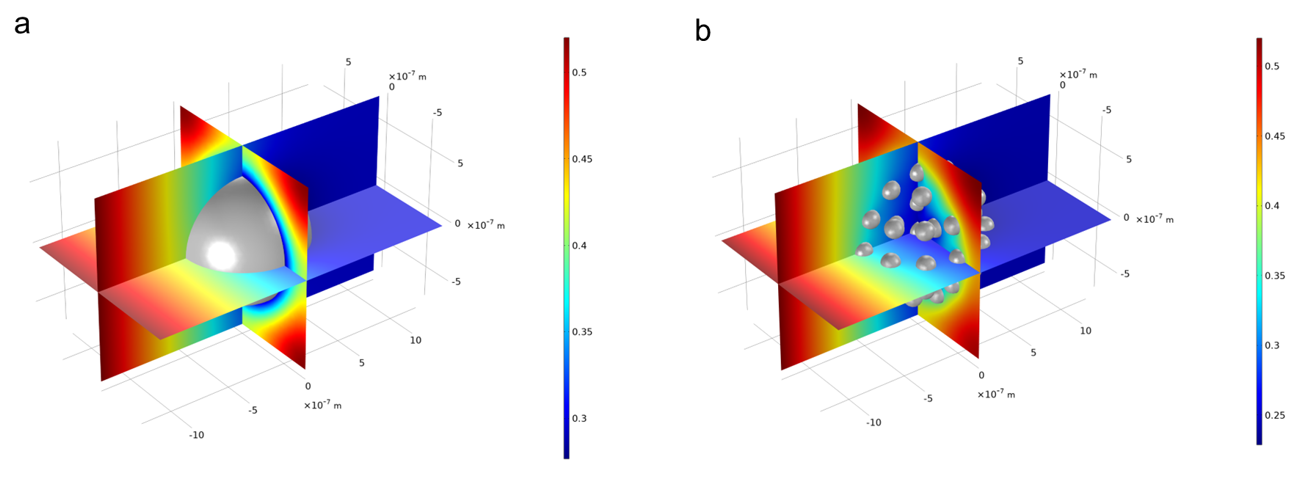


**Fig. S34.** The concentration distribution of O_3_ in (a) Model 1 and (b) Model 2.

**Note.** In these simulations, O_3_ molecules diffused to the surface of nanoparticles, undergo adsorption and activation, and then mineralize OA into CO_2_ and O_2_ products. The concentration distributions of reactants demonstrated that the penetrable channels in Model 2 resulted in the faster depletion of O_3_ in the void spaces between nanoparticles.


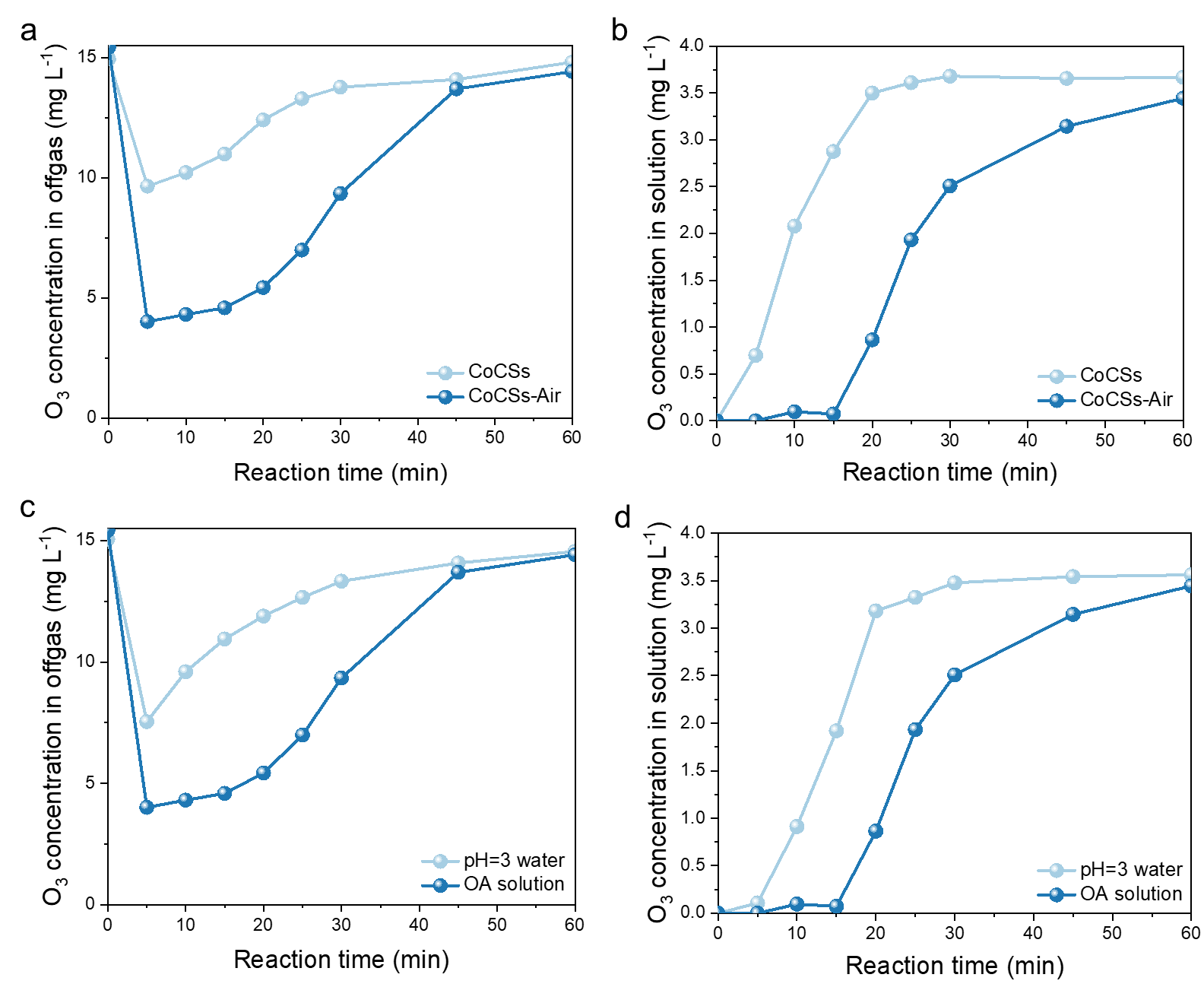


**Fig. S35.** Ozone concentrations in (a, c) offgas and (b, d) in reaction solution. Reaction conditions: catalyst loading: 0.01 g L^-1^; [OA]_0_: 150 mg L^-1^; ozone flow rate: 100 mL min^-1^; ozone concentration: 15 mg L^-1^; temperature: 25 °C; initial pH: 3.0.

**Note.** O_3_ utilization could be divided into two stages: rapid utilization and deceleration utilization. In the rapid utilization period, O_3_ was rapidly consumed and its concentration in the aqueous solution kept at a low level. However, with the reaction proceeding, O_3_ consumption gradually decreased. This is because the decreased OA concentration would reduce O_3_ consumption, resulting in the increase of O_3_ concentration in the liquid phase and consequently the lower mass transfer driving force. Without OA addition, the O_3_ concentration in the solution increased rapidly and the OUE was significantly lower than that in the presence of OA, further confirming the importance of concentration difference for facilitating the mass transfer of O_3_.

**Fig. S36.** Activation energies for CoCSs and CoCSs-Air.


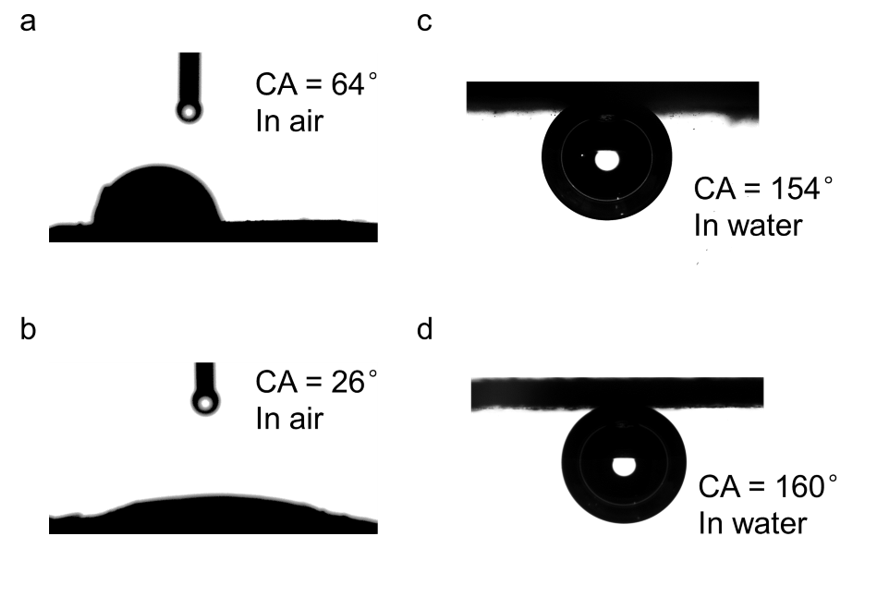


**Fig. S37.** Water CAs of (a) CoCSs and (b) CoCSs-Air. CAs of a gas bubble in aqueous solution of (c) CoCSs and (d) CoCSs-Air.

**Note.** The aerophobicity of the catalysts were also measured by examining the CAs of gas bubbles in the aqueous solution. The large CAs for both catalysts indicated their low surface affinity towards gaseous O_3_. The high hydrophilicity of CoCSs-Air and CoCSs allows them to directly leverage the dissolved ozone for activation.


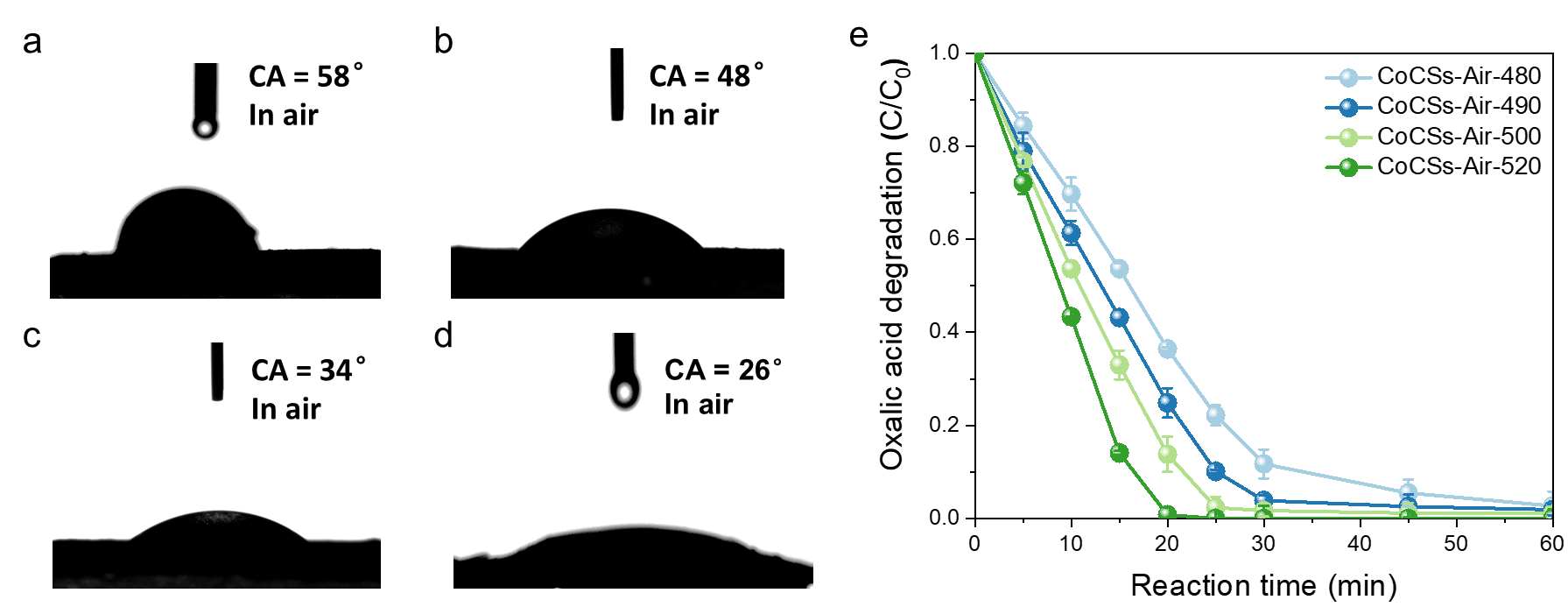


**Fig. S38.** The water CA of (a) CoCSs-Air-480, (b) CoCSs-Air-490, (c) CoCSs-Air-500 and (d) CoCSs-Air-520. (e) Degradation of OA over CoCSs-Air samples. Reaction conditions: catalyst loading: 0.02 g L^-1^; [OA]_0_: 150 mg L^-1^; ozone flow rate: 100 mL min^-1^; ozone concentration: 15 mg L^-1^; temperature: 25 °C; initial pH: 3.0.


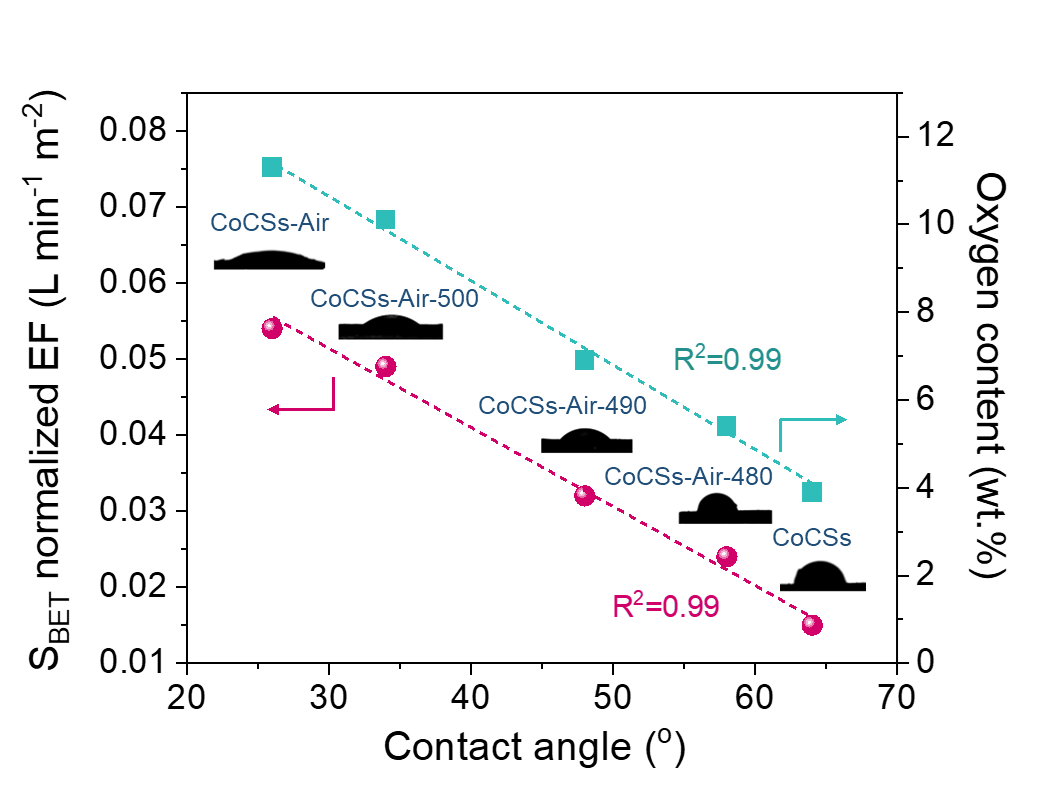


**Fig. S39.** Correlation of the S_BET_ normalized EF values and oxygen contents to contact angles of catalysts.

**
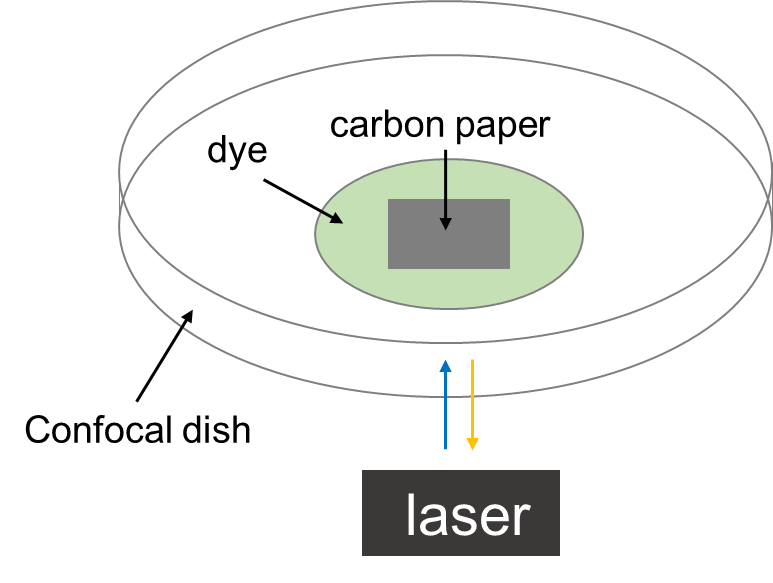
**

**Fig. S40.** Schematic illustration of the method used to probe the water penetration behavior on the catalyst layer by a confocal laser scanning microscopy.

**Fig. S41.** Z axis fluorescence intensity line scans from the cross-sectional images of CoCSs and CoCSs-Air.


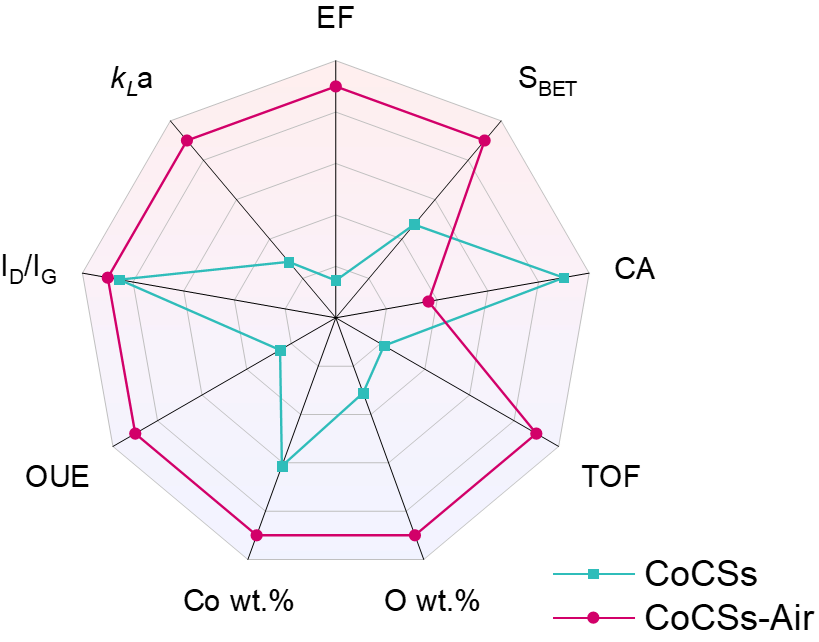


**Fig. S42.** Radar diagram of physiochemical properties (Co wt.%, O wt.%, S_BET_, CA, *k_L_*_a_ and I_D_/I_G_) and catalytic efficiencies (EF, TOF, and OUE) of CoCSs and CoCSs-Air.

**
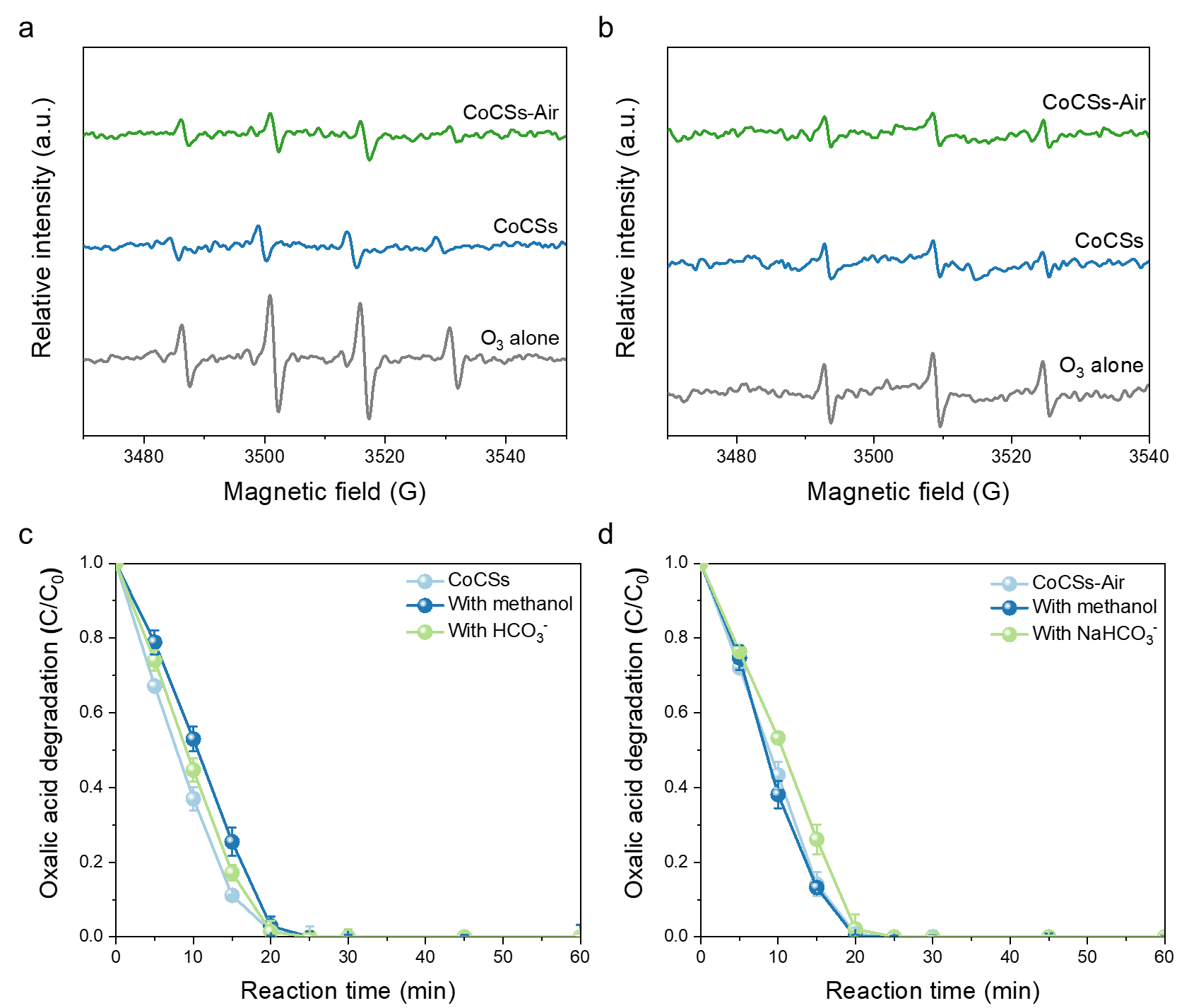
**

**Fig. S43.** EPR spectra with (a) DMPO and (b) TEMP as the spin trapping agents. Quenching tests in catalytic ozonation by (c) CoCSs and (d) CoCSs-Air. Reaction conditions: catalyst loading: 0.1 g L^-1^; [OA]_0_: 150 mg L^-1^; [Methanol]_0_ = [NaHCO_3_]_0_: 1 mmol; ozone flow rate: 100 mL min^-1^; ozone concentration: 15 mg L^-1^; temperature: 25 °C; initial pH: 3.0.

**Note.** The ROS were identified by EPR using DMPO and TEMP as the spin trapping agents to trap hydroxyl radicals (^•^OH)/superoxide radicals (O_2_^•–^) and singlet oxygen (^1^O_2_), respectively. The characteristic signals for DMPO-^•^OH adducts (hyperfine splitting couplings of A_N_ = A_H_ = 14.9 G) and triplet TEMPO signals (A_N_ = 16.9 G) were detected, while its signal intensity was weak and much lower than that in the solely ozonation system, suggesting that ^•^OH, O_2_^•–^ and ^1^O_2_ were not generated in the CoCSs/OA/O_3_ and CoCSs-Air/OA/O_3_ systems. Quenching tests were performed to investigate the reactive oxygen species (ROS) responsible for the organic destruction. Negligible inhibitory effects were observed in the presence of radical scavengers for ^•^OH, such as methanol and NaHCO_3_, indicating that ^•^OH in solution did not contribute to OA degradation. O_2_^•–^ and ^1^O_2_ with moderate oxidation ability can hardly mineralize OA.

**
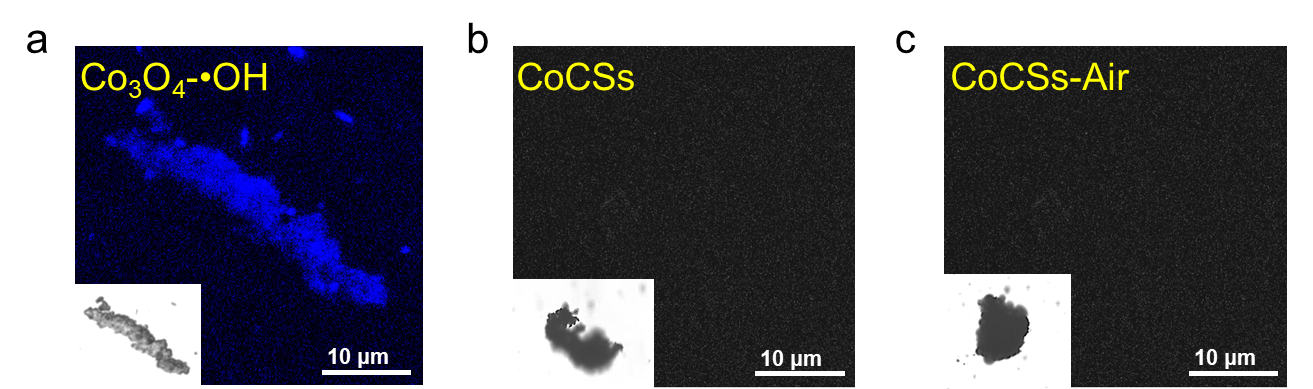
**

**Fig. S44.** FMI images of (a) Co_3_O_4_, (b) CoCSs and (c) CoCSs-Air.

**Note.** To observe the existence of surface-bound ^•^OH (^•^OH_ad_), we employed coumarin as a chemical probe, which could be oxidized by ^•^OH to generate fluorescent 7-hydroxycoumarin (7-HC). Using fluorescence microscopy image (FMI), the blue-colored region that ^•^OH produced on Co_3_O_4_ could be visualized. No fluorescence was detected around CoCSs and CoCSs-Air, excluding the presence of ^•^OH_ad_.

**
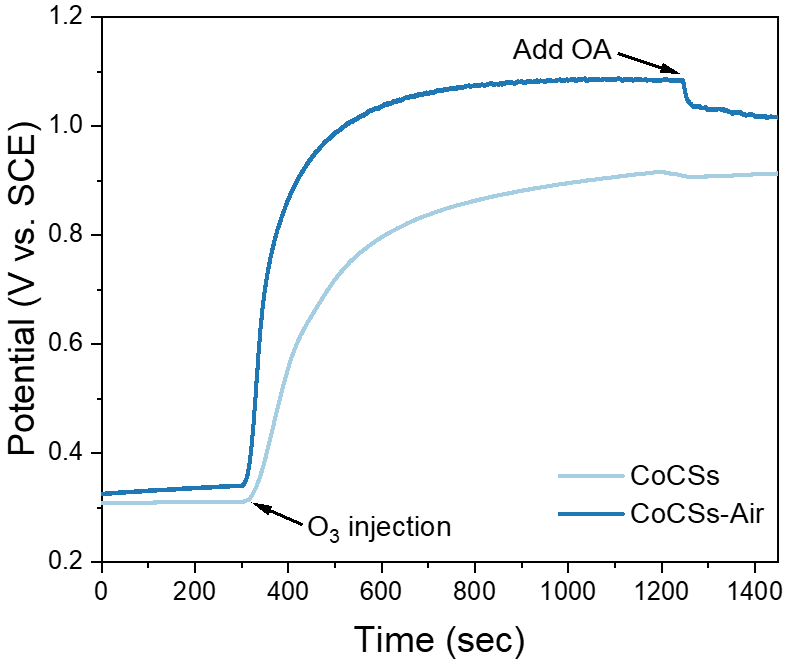
**

**Fig. S45.** Open-circuit potential curves of CoCSs and CoCSs-Air.


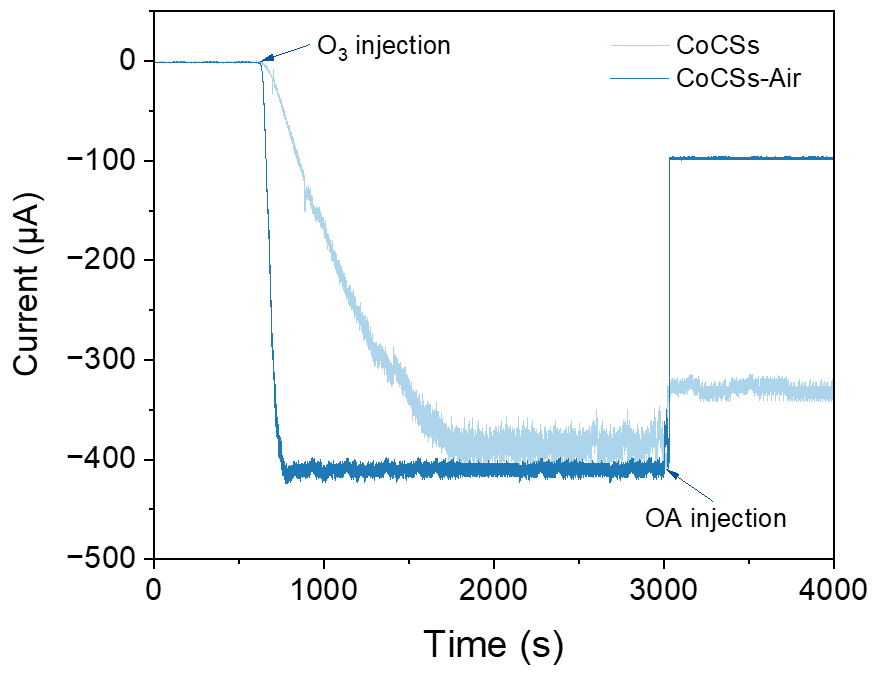


**Fig. S46.** *In situ* chronoamperometry response plots for CoCSs and CoCSs-Air upon injection of O_3_ and OA.

**
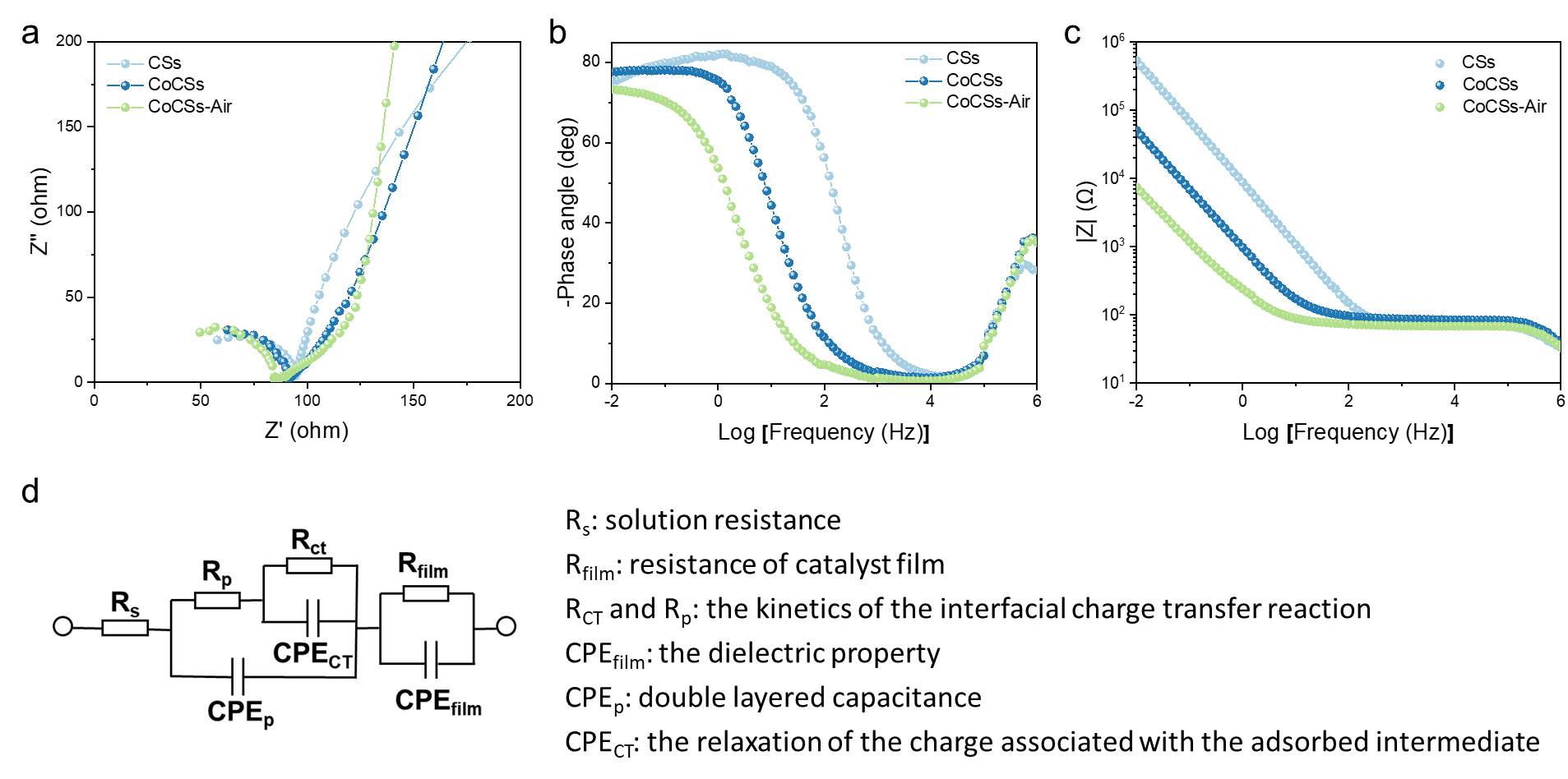
**

**Fig. S47.** (a) The Nyquist plots of CSs, CoCSs and CoCSs-Air and (b, c) the corresponding Bode plots. (d) The equivalent circuit diagram.


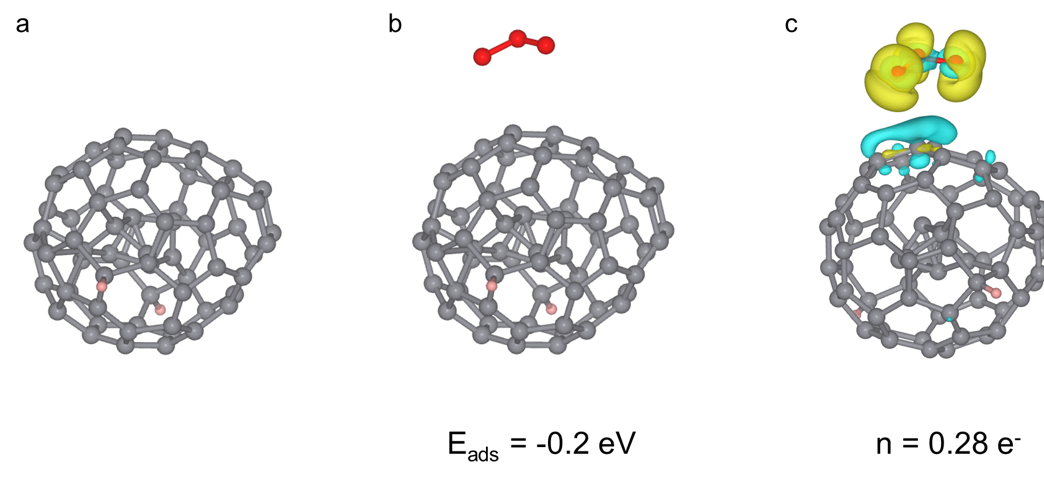


**Fig. S48.** (a) Amorphous carbon model. (b) Ozone adsorption on amorphous carbon model. (c) Differential charge density of amorphous carbon-O_3_. The red and grey spheres represent oxygen and carbon atoms, respectively.

**Note.** An ozone molecule remains intact when placed above the amorphous carbon, indicating the weak van der Waals forces between the amorphous carbon and O_3_. The adsorption energy of O_3_ on the amorphous carbon (-0.2 eV) and electron transfer number (0.28 e^-^) were significantly lower than those on Co-C_2_O_2_ model, suggesting its lower activation state.

**
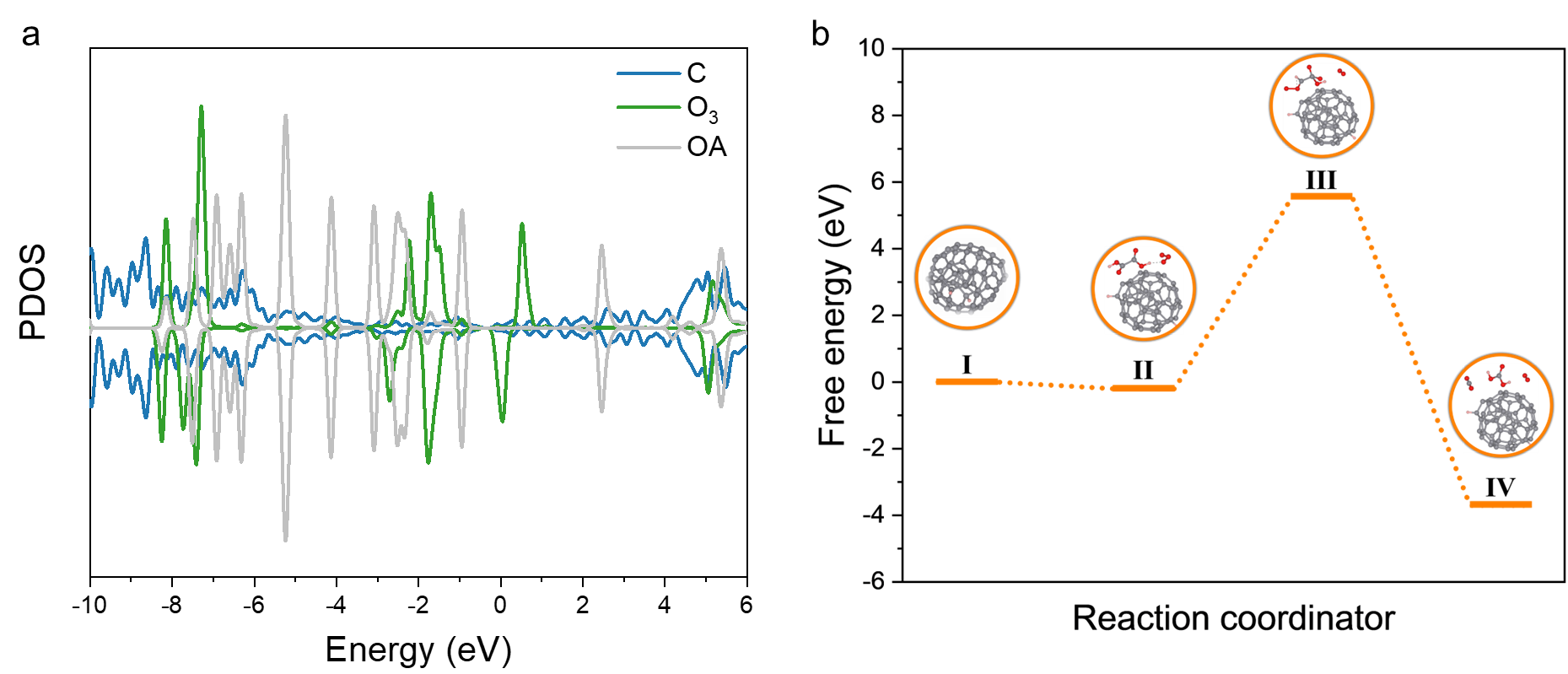
**

**Fig. S49.** (a) PDOS for C, O_3_ and OA molecules in amorphous carbon-O_3_. (b) Energy profile of O_3_ activation and subsequent OA oxidation on amorphous carbon model. The red and grey spheres represent oxygen and carbon atoms, respectively.

**Note.** The minor orbital overlaps between C, O_3_, and OA orbitals in amorphous carbon-O_3_ indicated a weak electronic interaction. Transition state analysis showed that OA oxidation on amorphous carbon requires a high reaction energy barrier (5.74 eV), suggesting its low oxidation ability, which is consistent with the experiment result (Fig. S27).


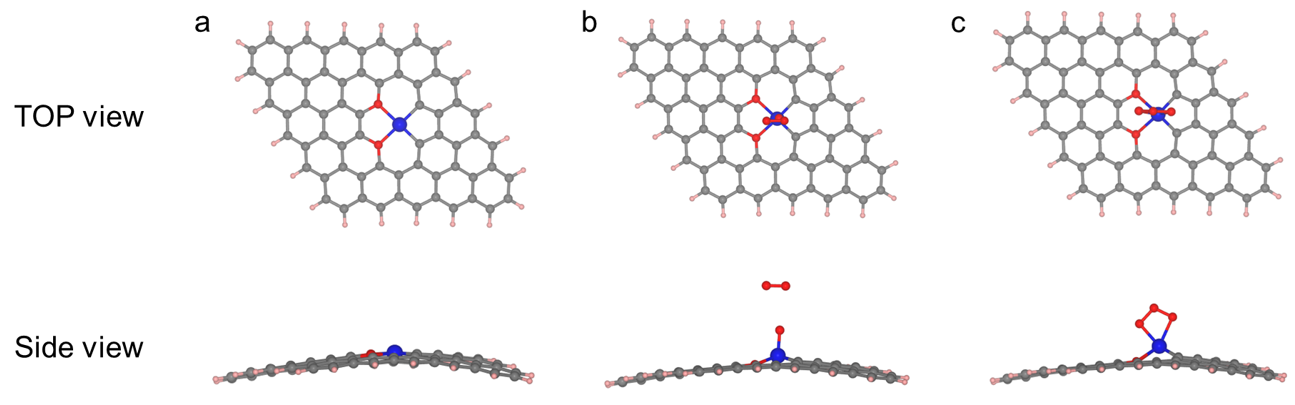


**Fig. S50.** (a) Co-C_2_O_2_ model. (b) “end-on” adsorption configuration of ozone. (c) “side-on” adsorption configuration of ozone. The blue, red and grey spheres represent cobalt, oxygen and carbon atoms, respectively.

**
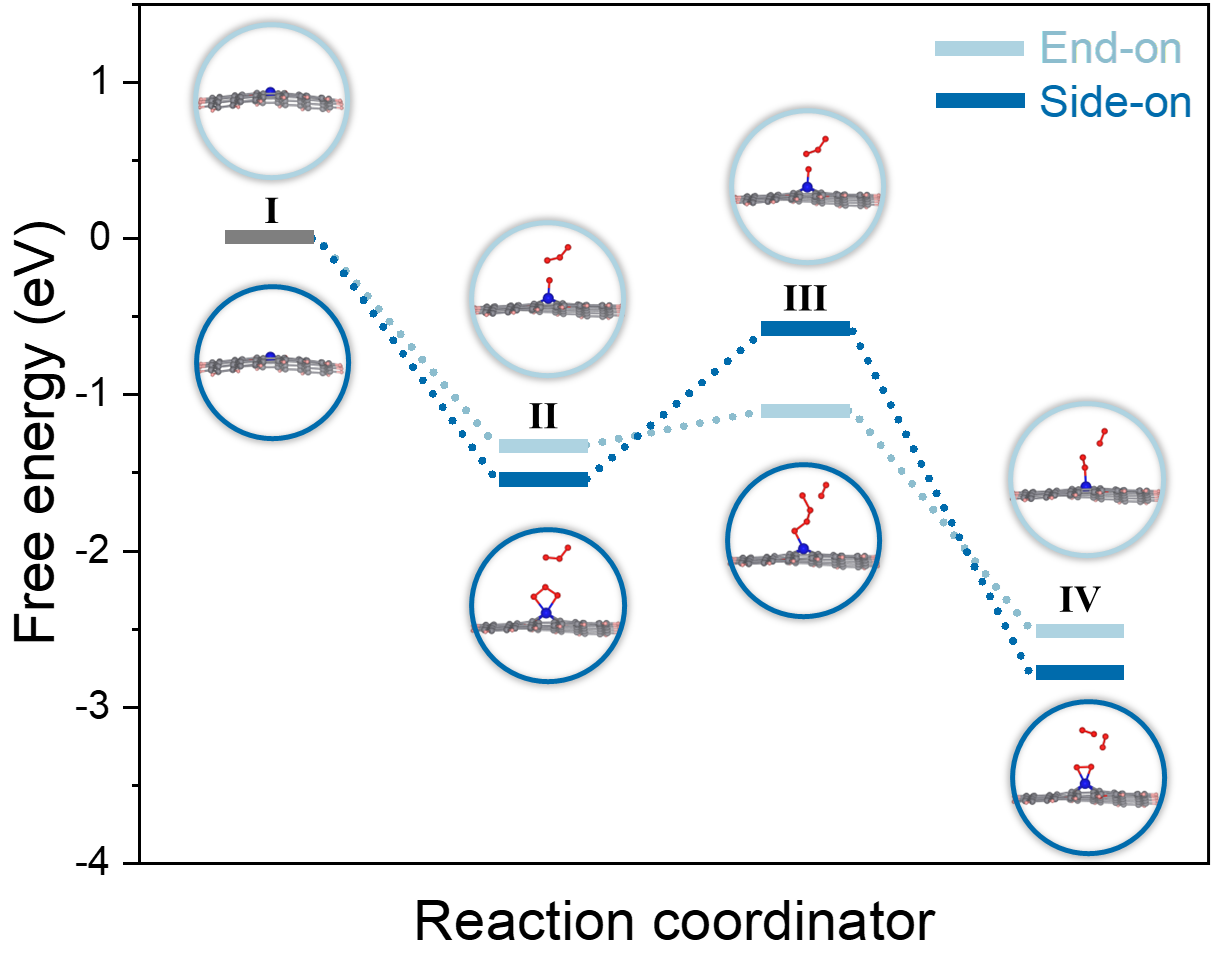
**

**Fig. S51.** Energy profiles of ozone activation and subsequent reactions with O_3_ molecule.

**Note.** In the “end-on” model, the Co-*O complex reacted with an O_3_ molecule, generating surface-adsorbed superoxide species. In contrast, the final products were bridging peroxide species and superoxide species in the “side-on” model. From *in situ* Raman spectra (Fig. 4a), we can clearly observe the peak at 1140 cm^-1^ in the CoCSs/O_3_ system, which can be assigned to the *η*^1^ superoxide species.^[12]^ The superoxide species can further transform into peroxide species, confirmed by the band of peroxide species at 812 cm^-1^. The strong peaks at 878 and 1045 cm^-1^ in the CoCSs-Air/O_3_ system correspond to the bridging peroxide species and superoxide species,^[13]^ respectively. Therefore, the theoretical results agree well with the experimental results.

**
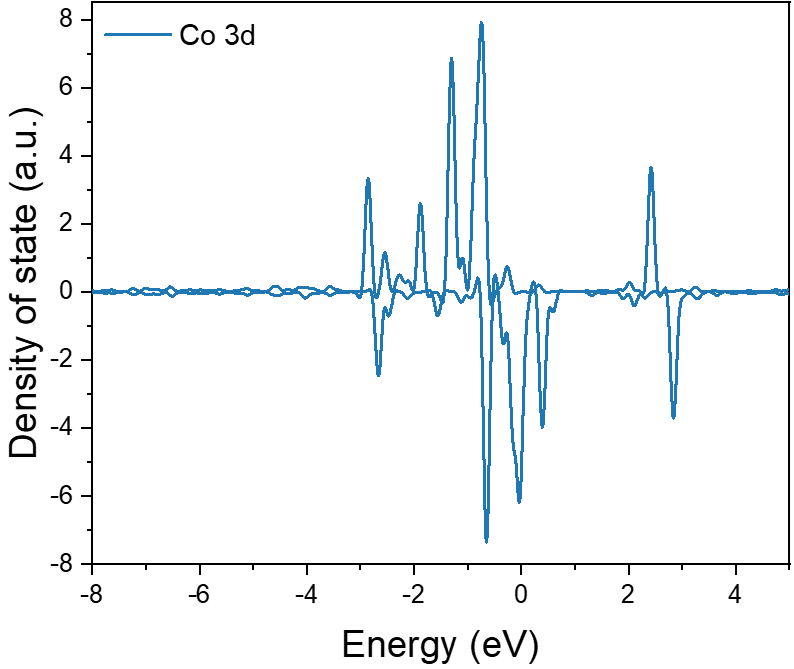
**

**Fig. S52.** PDOS for Co 3d orbital in Co-C_2_O_2_ model.

**Note.** Before the adsorption of O_3_, the pDOS of the Co sites showed an unsymmetrical arrangement of 3d orbital electrons in both spin channels. The effective magnetic moment (*μ*_eff_) of Co sites in CoCSs-Air obtained by the ZFC temperature-dependent magnetic susceptibility (χ) was 1.02 μB (Fig. S53), which was in line with the simulated magnetic moment of 0.95 μB. The charge density difference of Co-C_2_O_2_ model also revealed the asymmetric charge transfer between Co and O, resulting in the electron redistribution and charge polarization (Fig. S54). When O_3_ was adsorbed on the Co site, remarkably intensified spin polarization was observed in the scenarios of “side-on” mode (Fig. S55), which can also be confirmed by a larger magnetic moment of 1.73 μB of the Co atom in the Co-*O_3_ (Table S10). Nevertheless, the Co site in the Co-*O manifested weaker spin polarization compared with the initial state (0.81 vs. 0.95 μB). Moreover, the d-band center gap of Co spin-up and spin-down (Δd) of Co-*O and Co-*O_3_ were 0.80 and 1.67 eV (Table S11), respectively, signifying the distinct spin polarization.

**
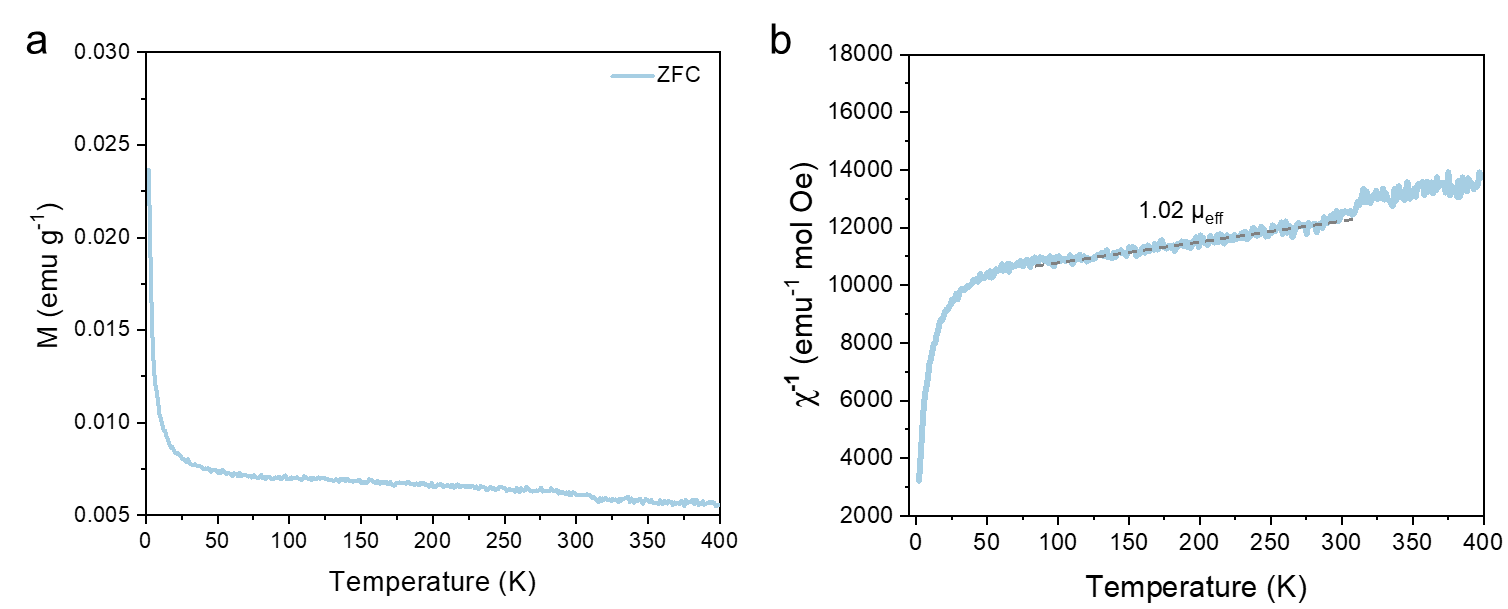
**

**Fig. S53.** (a) Zero field cooled (ZFC) curves of CoCSs-Air. (b) Temperature dependence inverse susceptibilities of CoCSs-Air.


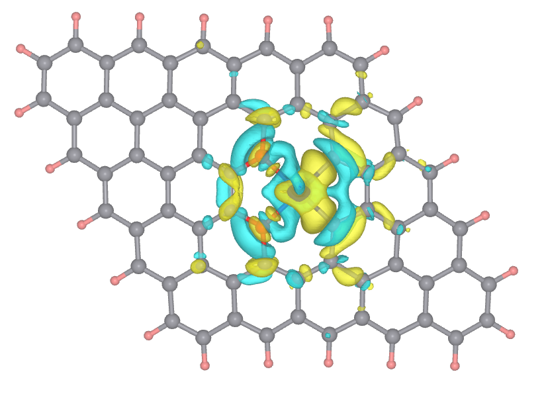


**Fig. S54.** Differential charge density of Co-C_2_O_2_ model. The blue, red and grey spheres represent cobalt, oxygen and carbon atoms, respectively.

**
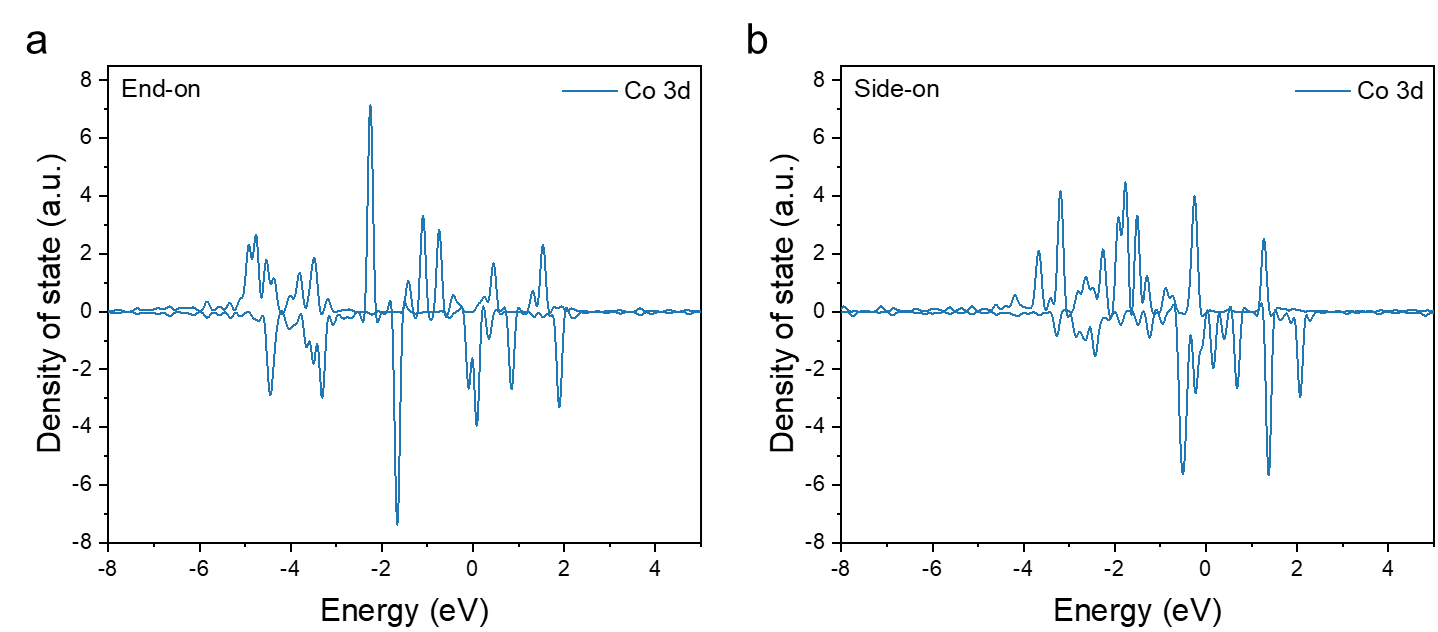
**

**Fig. S55.** PDOS for Co 3d orbital in (a) Co-*O and (b) Co-*O_3_.


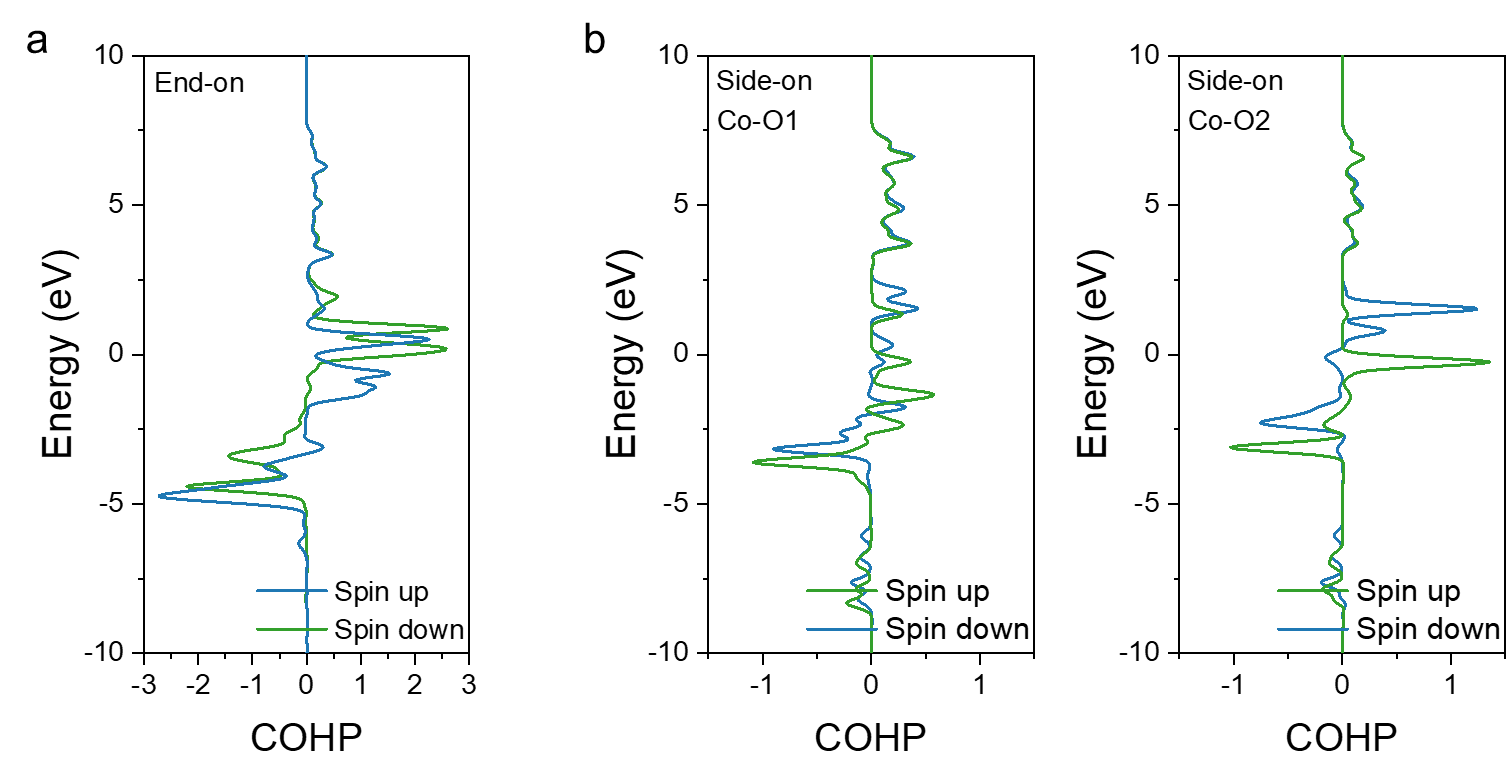


**Fig. S56.** COHP analysis of the Co-O bond in (a) Co-*O and (b) Co-*O_3_.

**Note.** Additionally, the bonding states of Co-O bond was disclosed by the crystal orbital Hamilton population (COHP) analysis, where the positive and negative parts of COHP below the Fermi level represent the antibonding and bonding states, respectively. For both Co-*O and Co-*O_3_ adsorption configurations, a more negative integrated COHP (ICOHP) value was obtained for the spin-down channel than that of the spin-up channel (Table S12), indicating that the Co-O bond strength mainly depended on the spin-down states. Moreover, as shown in Figs. 4d and 4e, the interaction of these Co 3d orbitals and O 2p orbitals were strong enough to split into bonding and antibonding orbitals. The antibonding orbitals of spin-up states were located below the *E*_f_, whereas those orbitals shifted positively crossing at the *E*_f_ in the spin-down states, further indicating the predominant contribution from the spin-down channel.

**
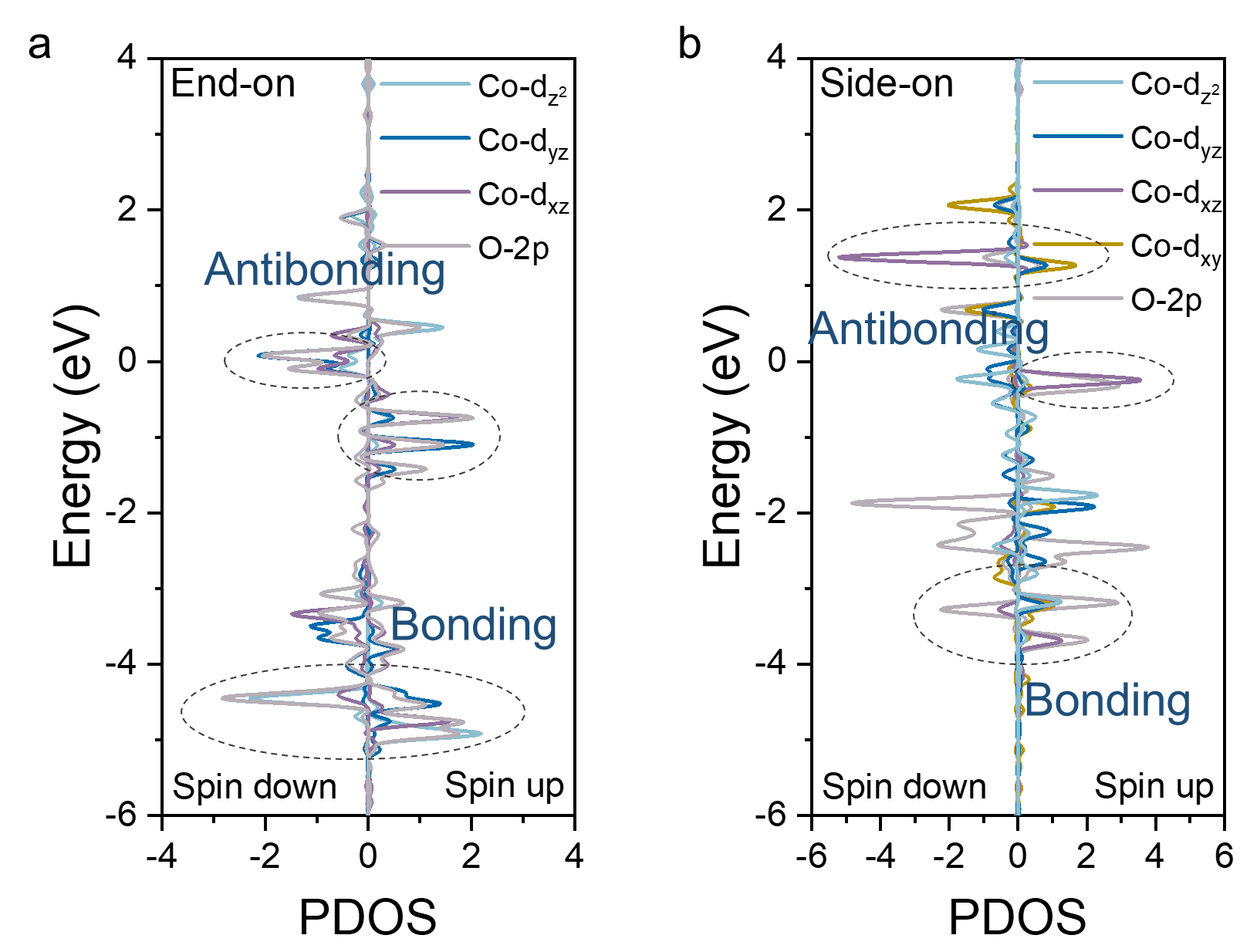
**

**Fig. S57.** PDOS of Co 3d sub-orbitals and O 2p orbital over (a) Co-*O and (b) Co-*O_3_.

**Note.** As illustrated in Fig. 4d, the smaller energy difference between Co 3d and O 2p centers and the larger orbital overlap in Co-*O indicated the enhanced Co-O covalency. Meanwhile, the downshift of Co d-band center (*E*_d_) demonstrated that the Co-O was more stable and less active in chemical bonding with other species (Table S11). In the scenarios for Co-*O_3_, the orbital overlap mainly occurred around the *E*_f_ especially in the spin-down channel, giving rise to the more pronounced energy level splitting and spin polarization of Co 3d orbital near the *E*_f_ (Fig. 4e). The stronger electronic coupling and interaction near the *E*_f_ evidenced that the Co-*O_3_ was in a higher activation state. Based on the d-orbital splitting manner analysis (Fig. S59 and Table S13), the highly localized distribution of the energy levels of five Co 3d orbitals in Co-*O_3_ near the *E*_f_ revealed a much higher electrons density, which was also confirmed by the charge density distribution maps (Fig. S60). It suggested that Co-*O_3_ was more favorable for the electron transfer and thus benefited the oxidation of organic pollutants.

**
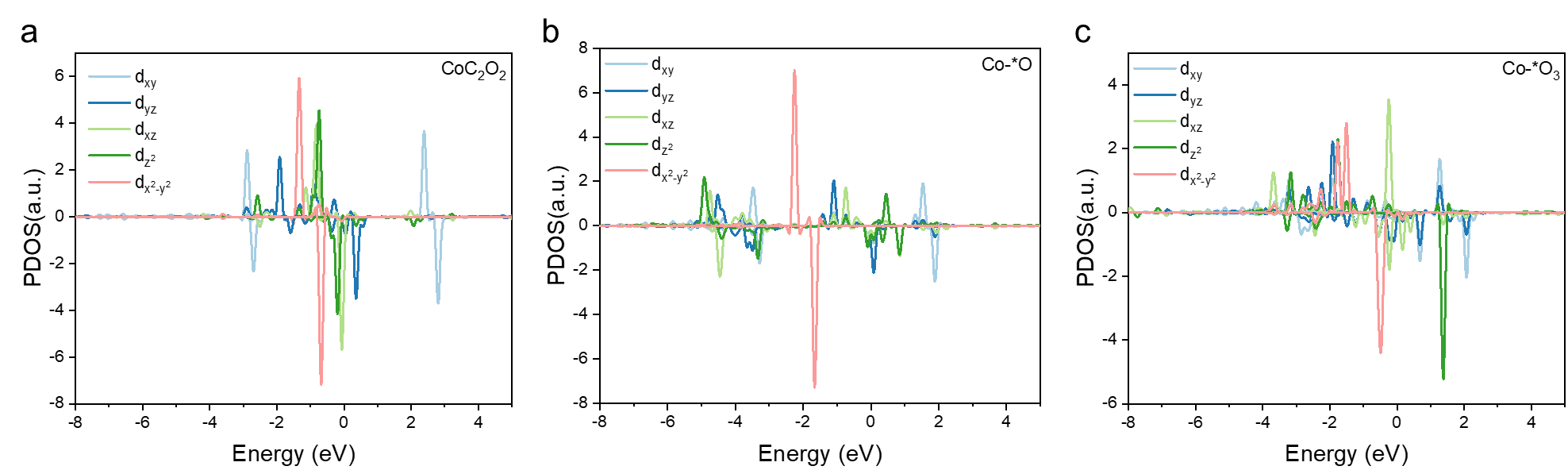
**

**Fig. S58.** PDOS of Co 3d sub-orbitals over (a) Co-C_2_O_2_ model, (b) Co-*O and (c) Co-*O_3_.

**
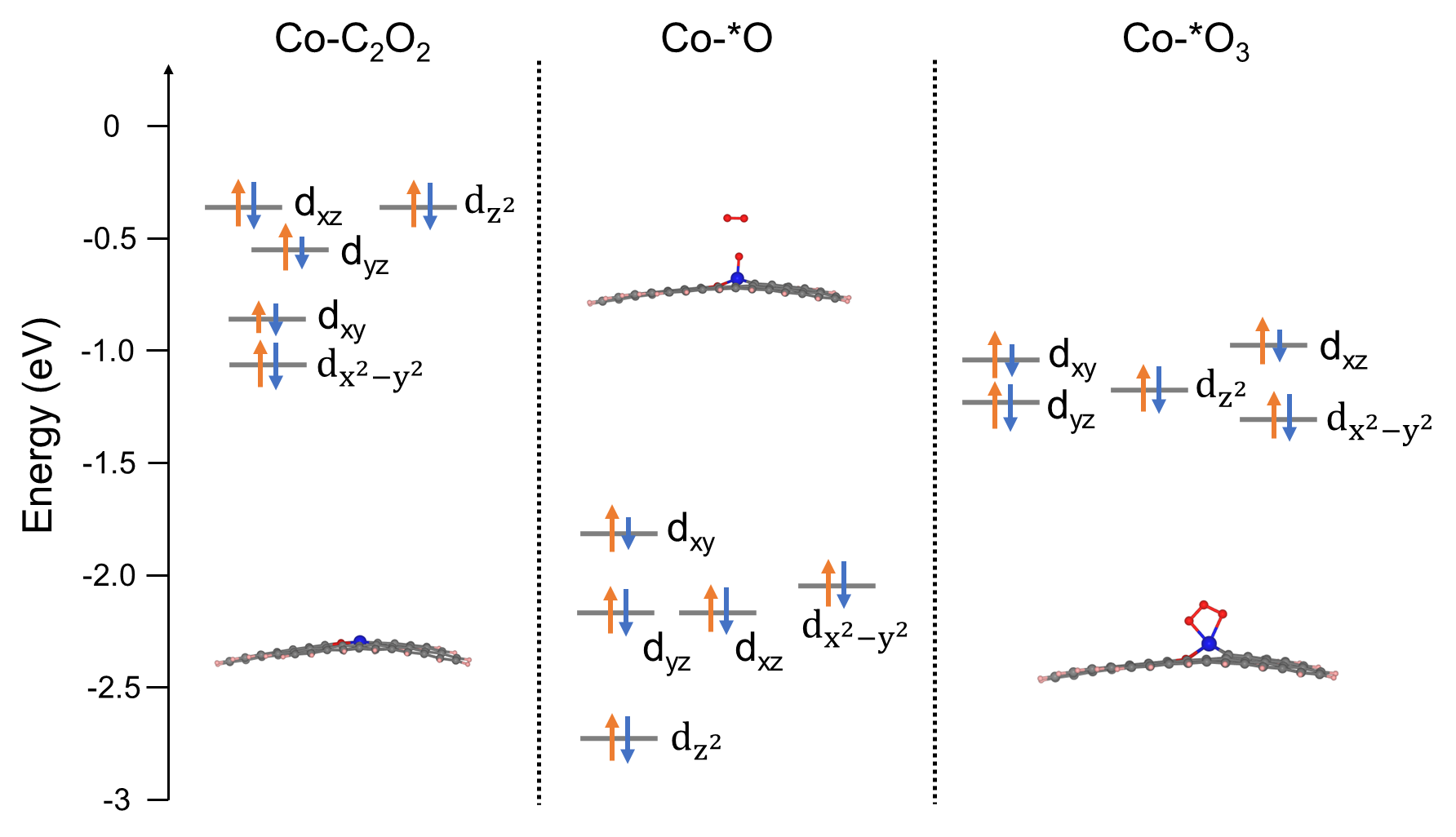
**

**Fig. S59.** The energy configuration of Co 3d orbitals over Co-C_2_O_2_, Co-*O and Co-*O_3_.


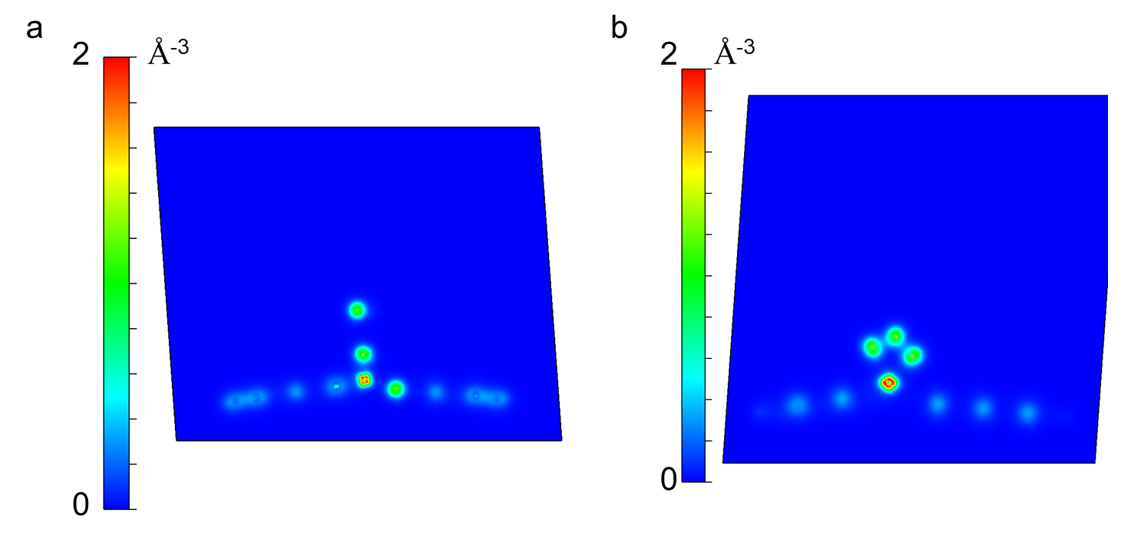


**Fig. S60.** The charge density distribution maps of (a) Co-*O and (b) Co-*O_3_.


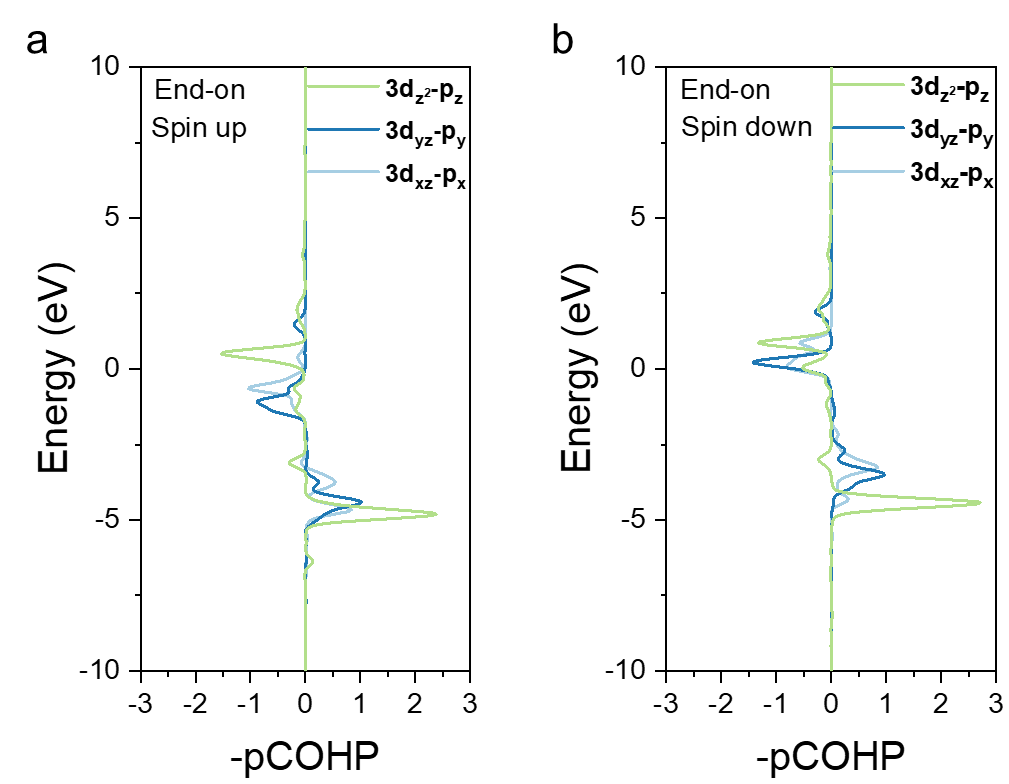


**Fig. S61.** (a, b) pCOHP analysis of the Co-O bond in Co-*O.

**
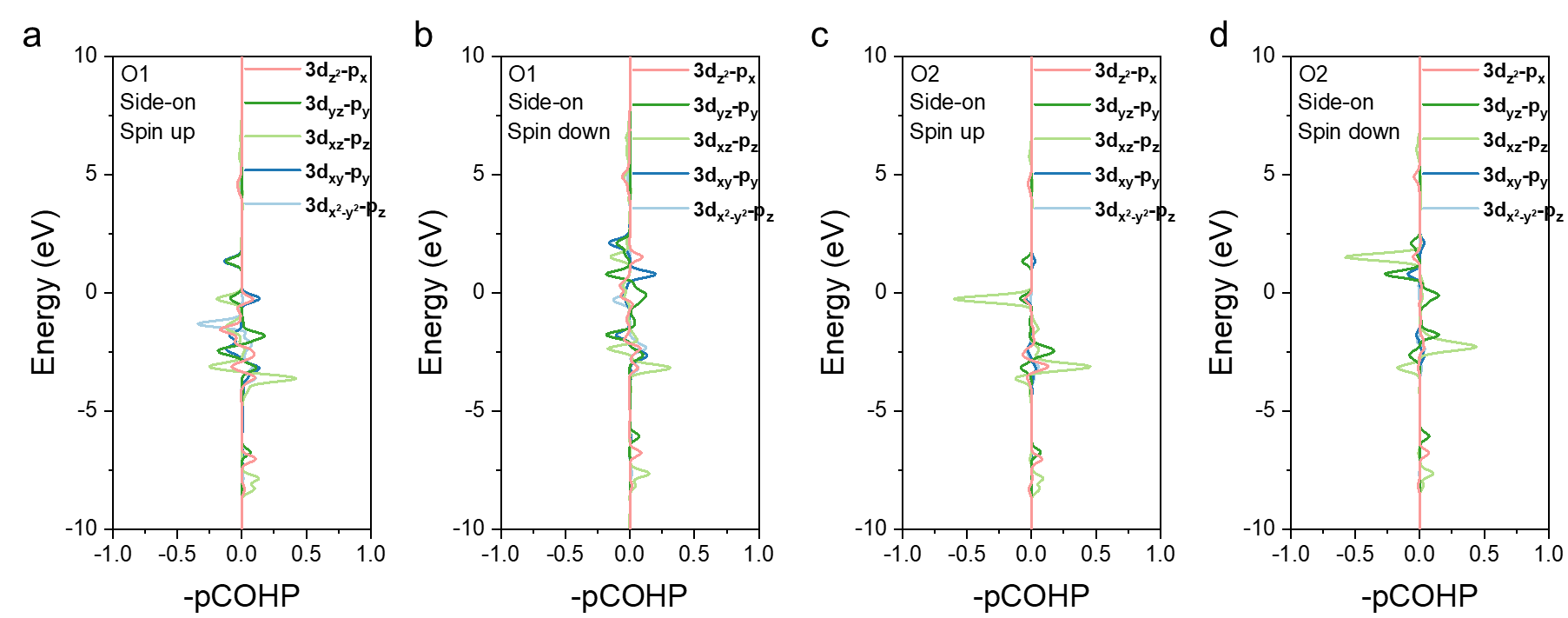
**

**Fig. S62.** (a-d) pCOHP analysis of the Co-O bond in Co-*O_3_.


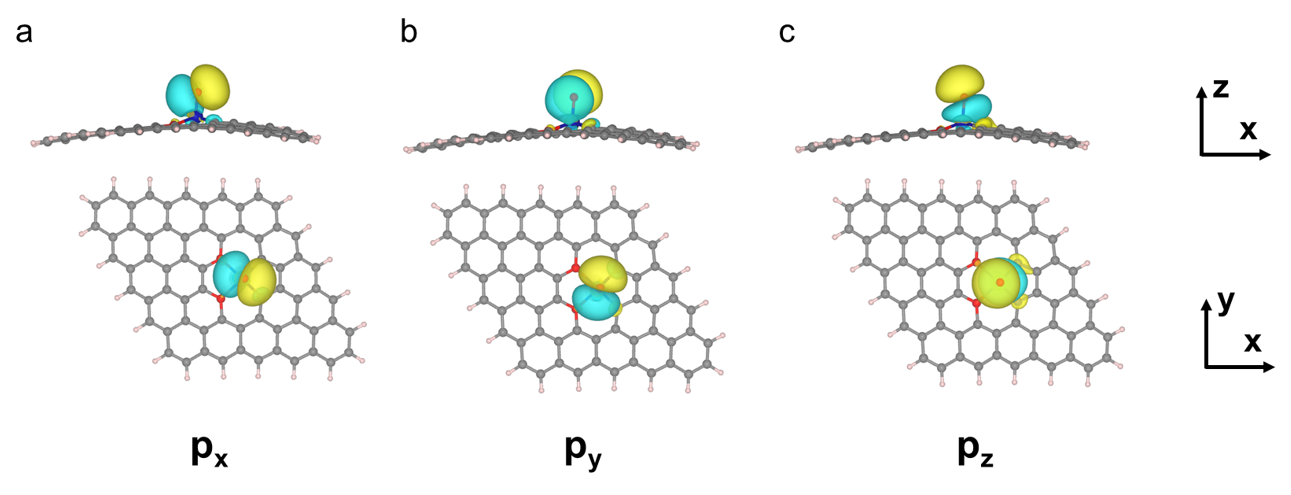


**Fig. S63.** Top and side view of maximally localized Wannier functions of O p orbitals in Co-*O model.


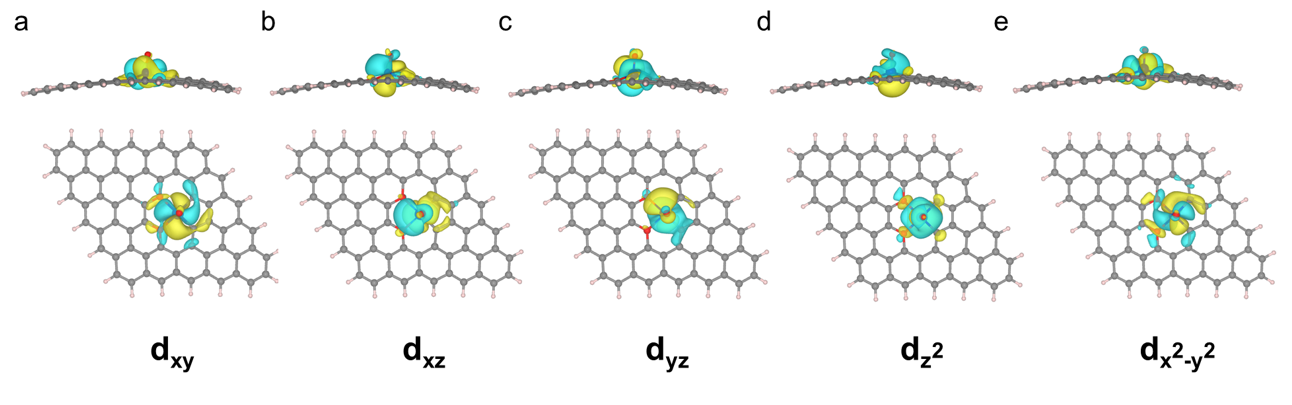


**Fig. S64.** Top and side view of maximally localized Wannier functions of Co d orbitals in Co-*O model.


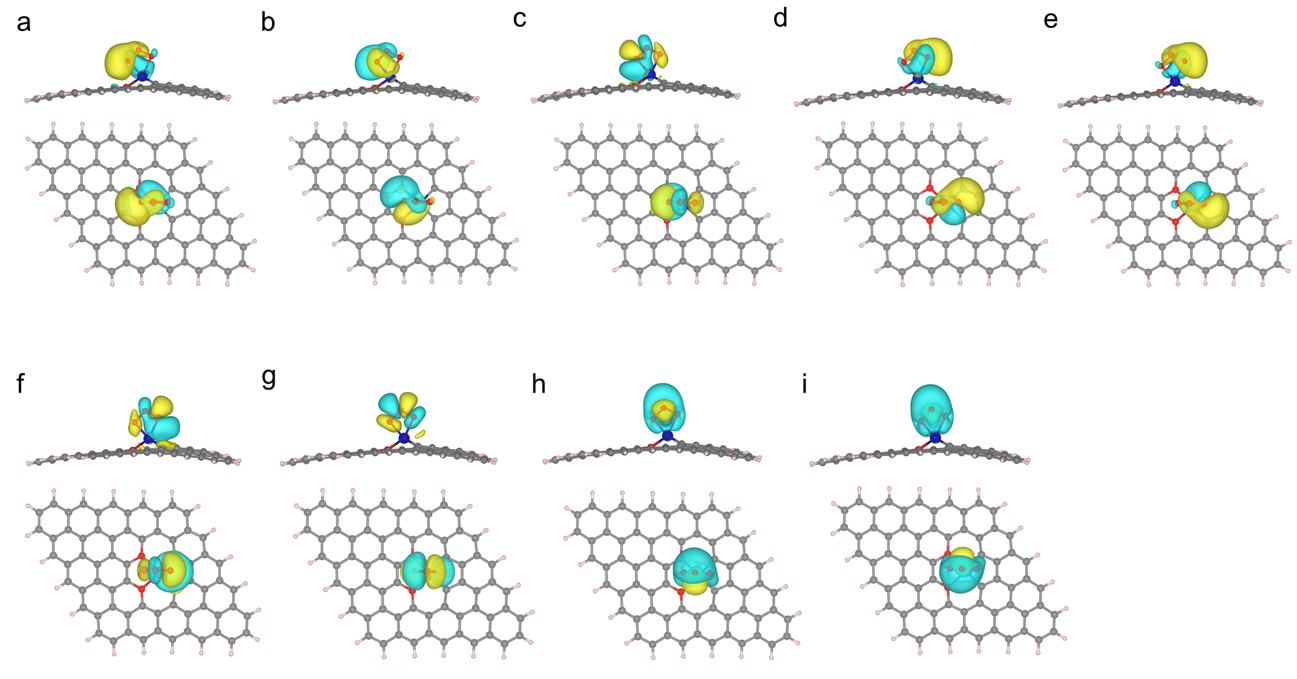


**Fig. S65.** Top and side view of maximally localized Wannier functions of O p orbitals in Co-*O_3_ model.


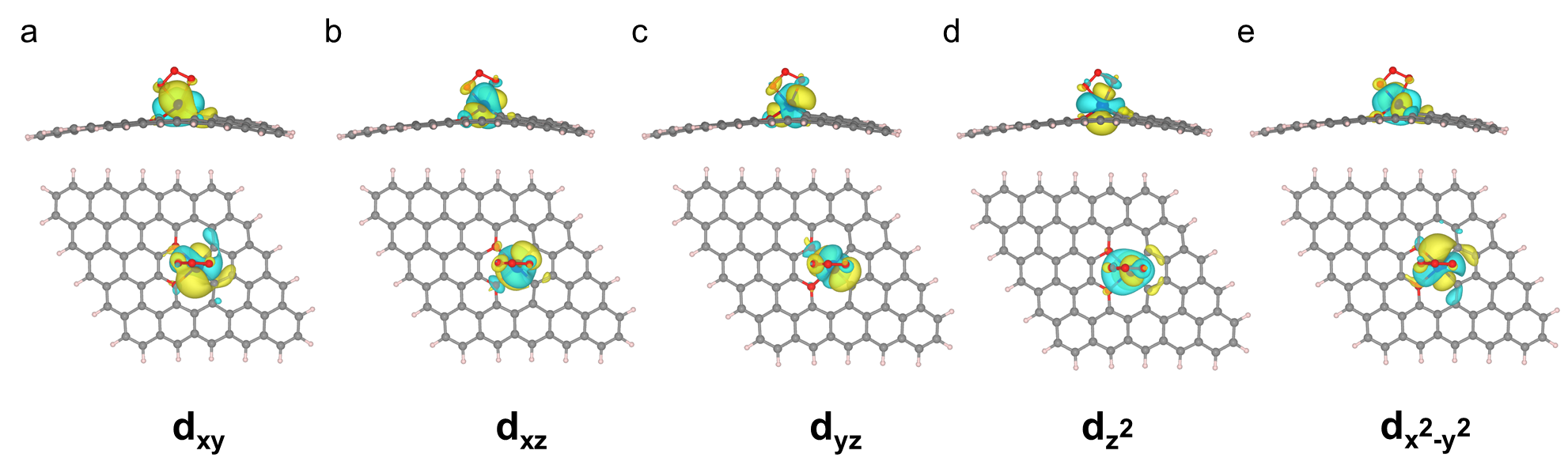


**Fig. S66.** Top and side view of maximally localized Wannier functions of Co d orbitals in Co-*O_3_ model.

**
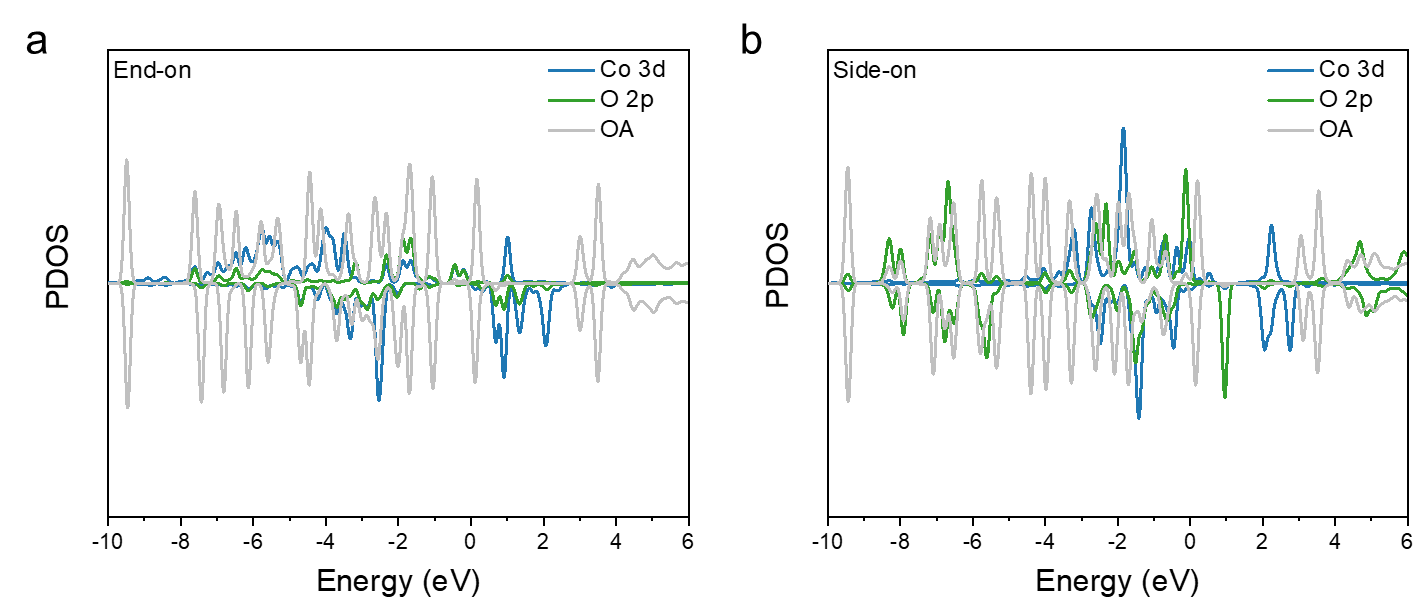
**

**Fig. S67.** PDOS for Co 3d orbital, O 2p orbital and OA molecule in (a) Co-*O and (b) Co-*O_3_.

**Note.** After OA adsorption, the Δd and magnetic moment of Co in Co-*O_3_ were significantly reduced (Tables S10 and S11), which could be ascribed to the strong hybridization between Co-*O_3_ and OA. Compared with Co-O, the higher overlap between the Co 3d orbital, O 2p orbital and OA in Co-*O_3_ demonstrated the stronger electronic interaction and enhanced electron transfer.


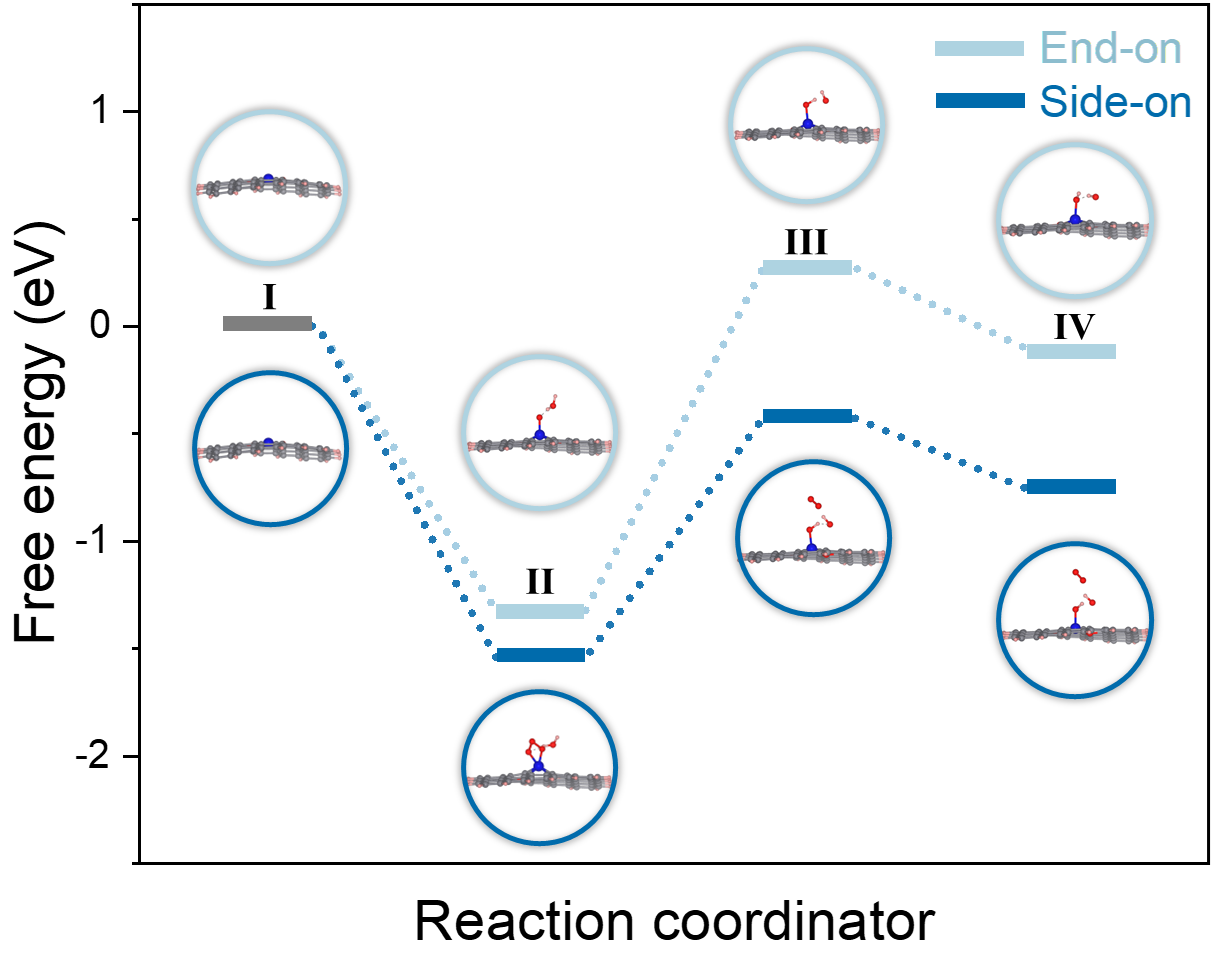


**Fig. S68.** Energy profiles of ozone activation and subsequent reactions with H_2_O.

**Note.** Transition state energy profiles for both “side-on” and “end-on” models were analyzed in their interactions with ambient O_3_ (Fig. S51) and H_2_O molecules to assess the impact of adsorption configurations on the generation of side products. The lower energy barrier for the reactions between Co-*O and O_3_ compared with OA oxidation reaction suggested that the former was more thermodynamically favorable for generating peroxide species, which would subsequently evolve into H_2_O_2_ as the side product (Eqs. S1-S3). However, benefiting from the greater thermodynamically feasibility of the reaction between Co-*O_3_ and OA, the OA oxidation reaction preferentially happened. Additionally, we considered the reaction with H_2_O molecules for H_2_O_2_ generation (Eqs. S4-S6). The reactions of both Co-*O_3_ and Co-*O complexes with OA molecules obtained lower energy barriers compared to the reactions with ambient H_2_O molecules. Therefore, the generation of H_2_O_2_ primarily resulted from the reaction with O_3_. This was further corroborated by n,n-diethyl-p-phenylenediamine/horseradish peroxidase (DPD/POD) measurements, in which a higher amount of H_2_O_2_ was generated in CoCSs/O_3_/OA than that in CoCSs-Air/O_3_/OA (Fig. S69). Both the CoCSs and CoCSs-Air were unable to catalyze the decomposition of H_2_O_2_. The lower molar ratio of consumed O_3_ to degraded OA (denoted as *R* value) in CoCSs-Air/O_3_/OA compared to CoCSs/O_3_/OA indicated the higher O_3_ conversion rate and utilization efficiency (1.0 versus 2.4).

**
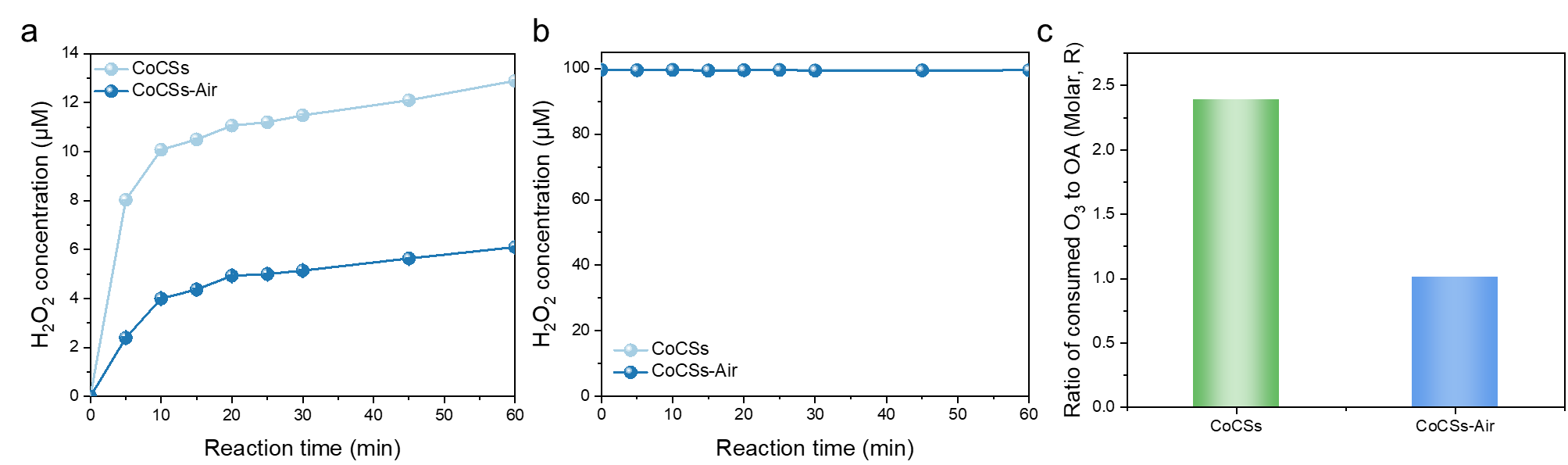
**

**Fig. S69.** (a) Time-dependent H_2_O_2_ concentration in CoCSs/O_3_/OA and CoCSs Air/O_3_/OA systems. Reaction conditions: catalyst loading: 0.01 g L^-1^; [OA]_0_: 150 mg L^-1^; ozone flow rate: 100 mL min^-1^; ozone concentration: 15 mg L^-1^; temperature: 25 °C; initial pH: 3.0. (b) Catalytic activity of H_2_O_2_ decomposition over CoCSs and CoCSs-Air. Reaction conditions: catalyst loading: 0.01 g L^-1^; [H_2_O_2_]_0_: 100 μM; temperature: 25 °C; initial pH: 3.0. (c) The molar ratio of consumed O_3_ to degraded OA in CoCSs/O_3_/OA and CoCSs-Air/O_3_/OA systems.

**
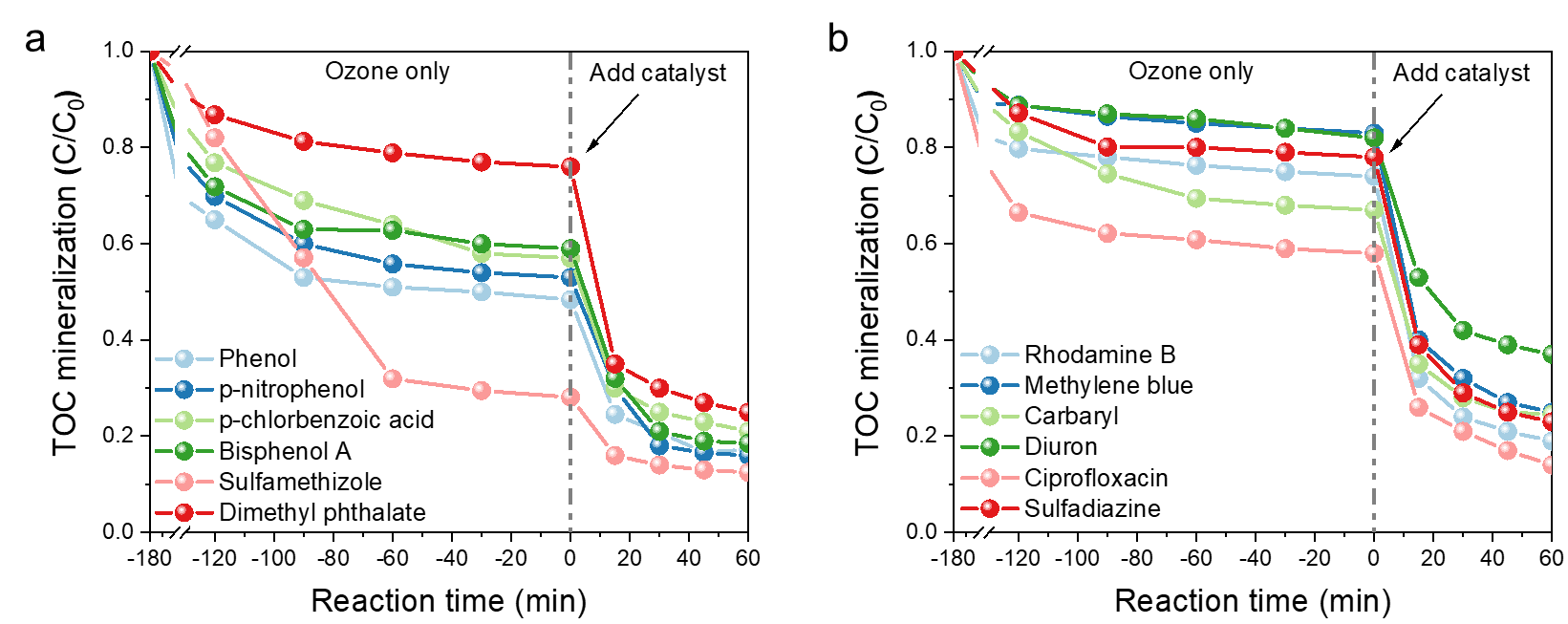
**

**Fig. S70.** (a, b) TOC mineralization rates of various pollutants using CoCSs-Air as the catalyst. Reaction conditions: catalyst loading: 0.01 g L^-1^; [pollutant]_0_: 50 mg L^-1^; ozone flow rate: 100 mL min^-1^; ozone concentration: 15 mg L^-1^; temperature: 25 °C; initial pH: 3.0.

**
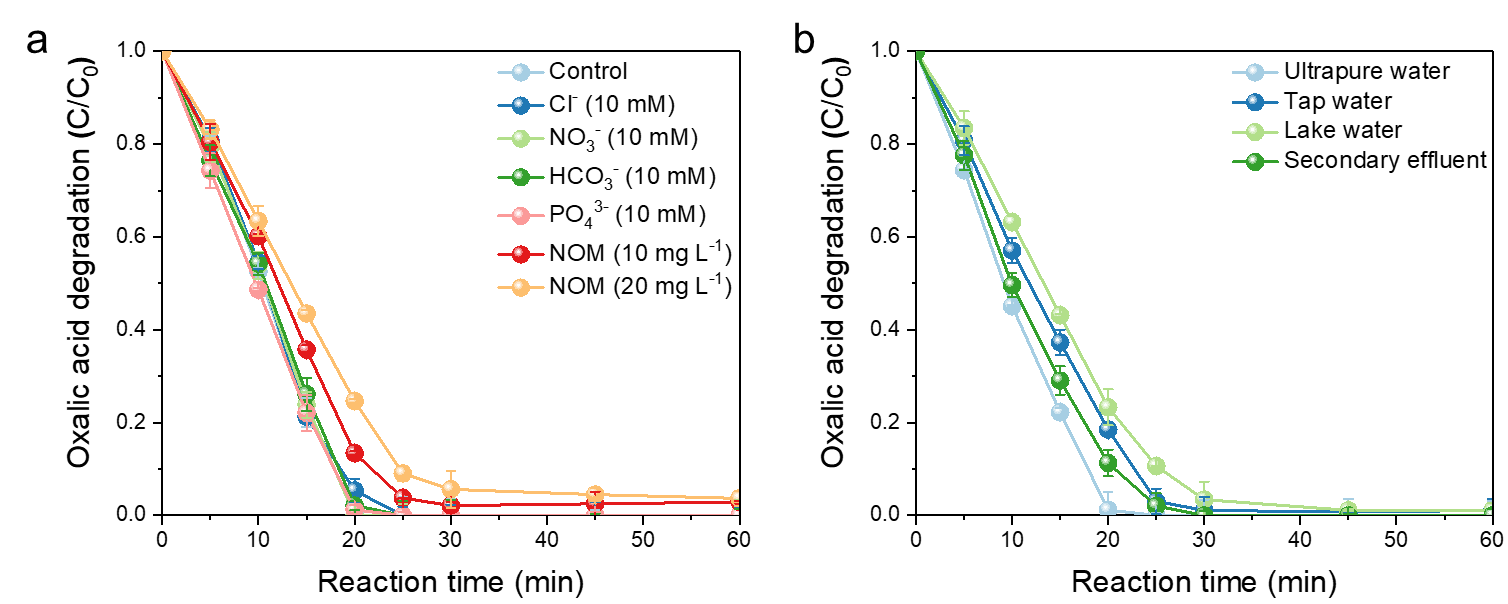
**

**Fig. S71.** (a) Effects of inorganic anions and HA on the catalytic ozonation efficiency over CoCSs-Air. (b) Effects of reaction media on the catalytic ozonation efficiency over CoCSs-Air. Reaction conditions: catalyst loading: 0.025 g L^-1^; [OA]_0_: 150 mg L^-1^; ozone flow rate: 100 mL min^-1^; ozone concentration: 15 mg L^-1^; temperature: 25 °C; initial pH: 3.0.


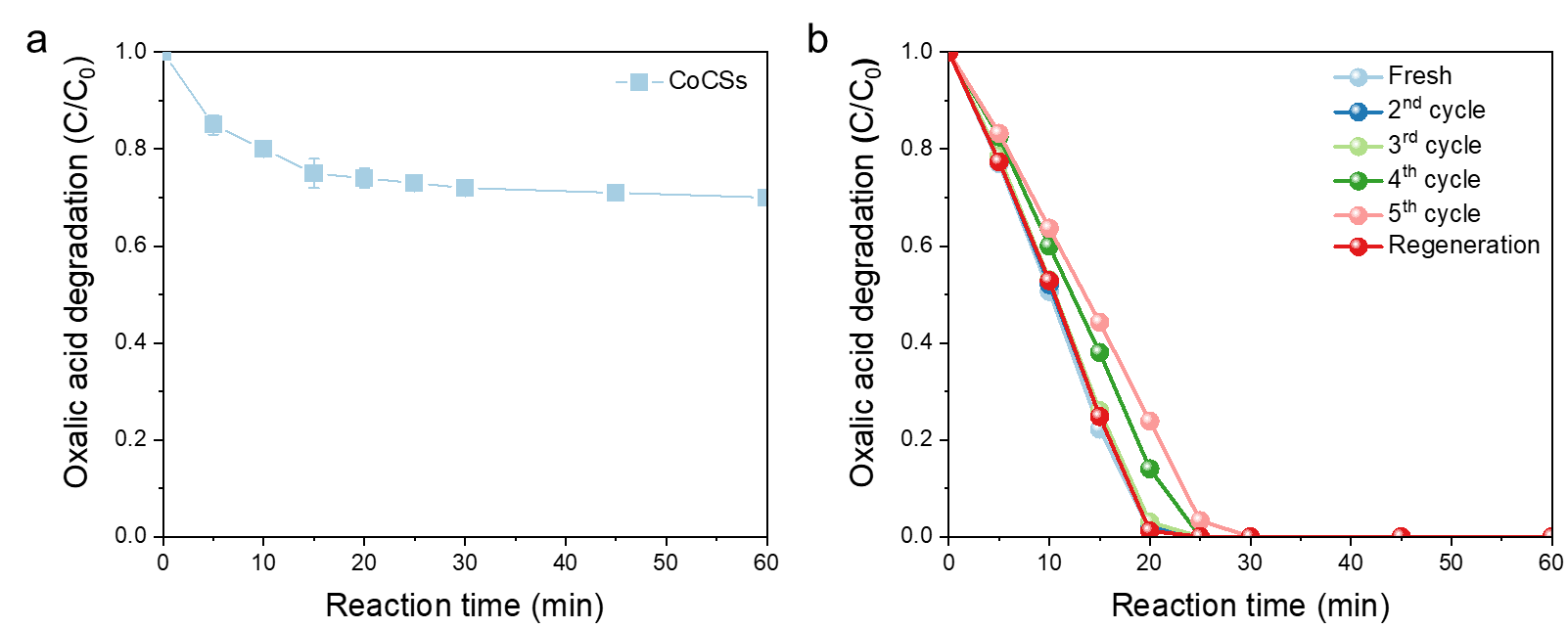


**Fig. S72.** (a) Degradation efficiency of CoCSs. (b) Reusability tests on CoCSs-Air. Reaction conditions: catalyst loading: 0.025 g L^-1^; [OA]_0_: 150 mg L^-1^; ozone flow rate: 100 mL min^-1^; ozone concentration: 15 mg L^-1^; temperature: 25 °C; initial pH: 3.0.

**Note.** Unlike CoCSs, which was fully passivated after 20 min, CoCSs-Air maintained a complete OA removal ability even after five usage cycles.


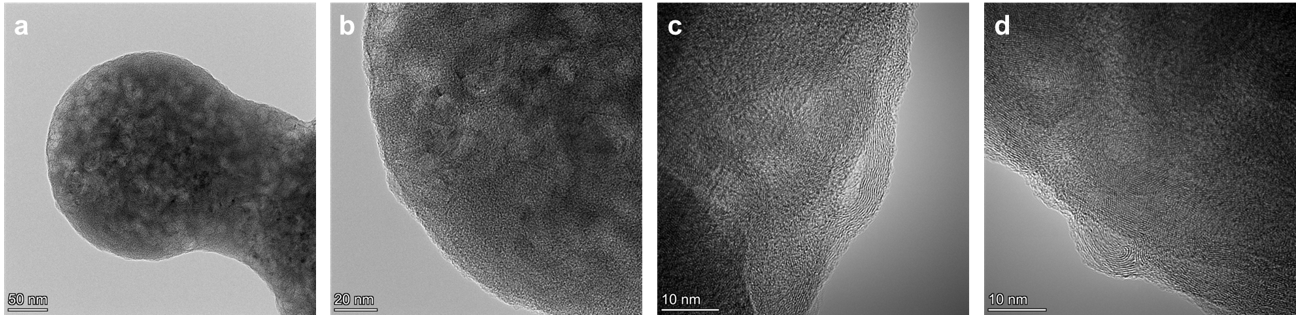


**Fig. S73.** (a-d) TEM images of CoCSs-Used.

**Note.** HRTEM images evidenced the collapse of graphitic channels inside the deactivated CoCSs. The amorphous carbon structure in CoCSs is vulnerable to be attacked by O_3_ molecules and dissociate into sticky carbonaceous debris blocking the active sites.


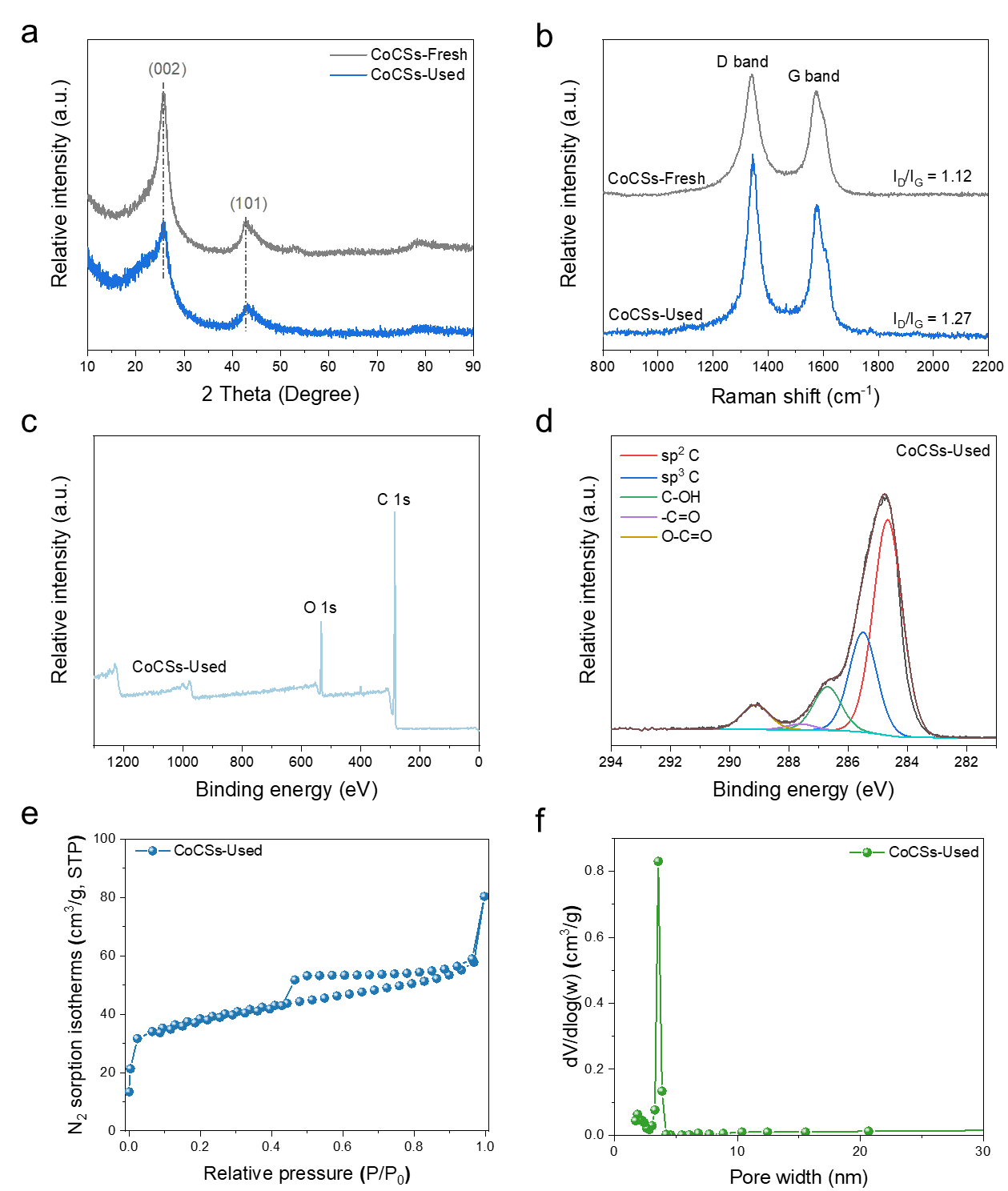


**Fig. S74.** (a) XRD patterns and (b) Raman spectra of CoCSs and CoCSs-Used. (c) XPS full survey, (d) High-resolution C1s spectra, (e) N_2_ sorption isotherms and (f) the corresponding Barrett-Joyner-Halenda (BJH) pore size distributions of the CoCSs-Used.

**Note.** As revealed by the XRD, Raman and XPS analyses, the deactivated CoCSs exhibited an increased defective level and higher oxidation degree. Moreover, the S_BET_ and total pore volume of the deactivated CoCSs shrunk dramatically to 140 m^2^ g^-1^ and 0.08 cm^3^ g^-1^, respectively, compared to the fresh one. The change in surface chemistry and the destruction of carbon skeleton might be ascribed to the oxidation by excessive O_3_ and the generated ROS.


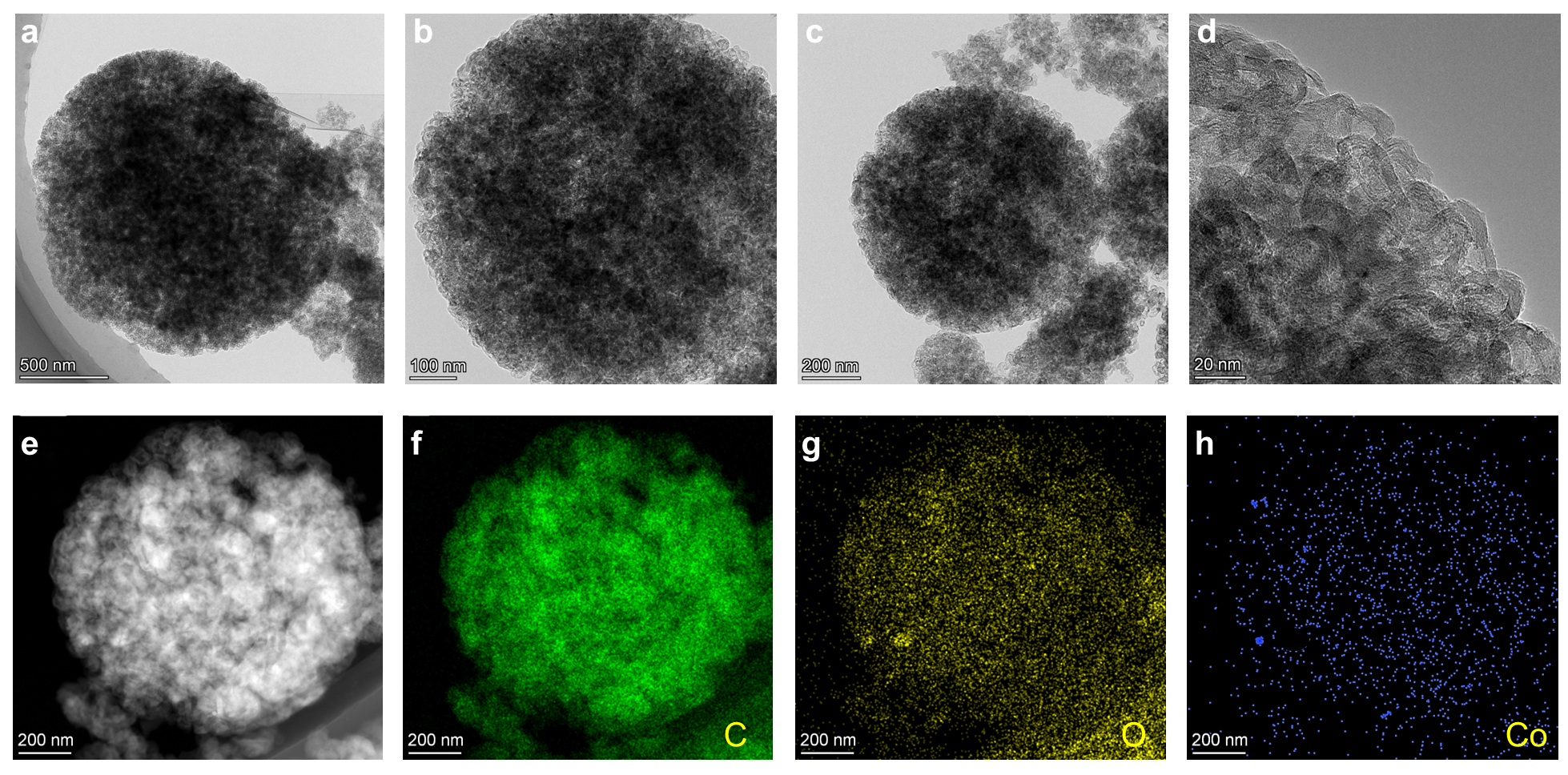


**Fig. S75.** (a-d) TEM images of CoCSs-Air-Used. (e-h) HAADF-STEM image and corresponding elemental mapping images of C, O and Co.

**
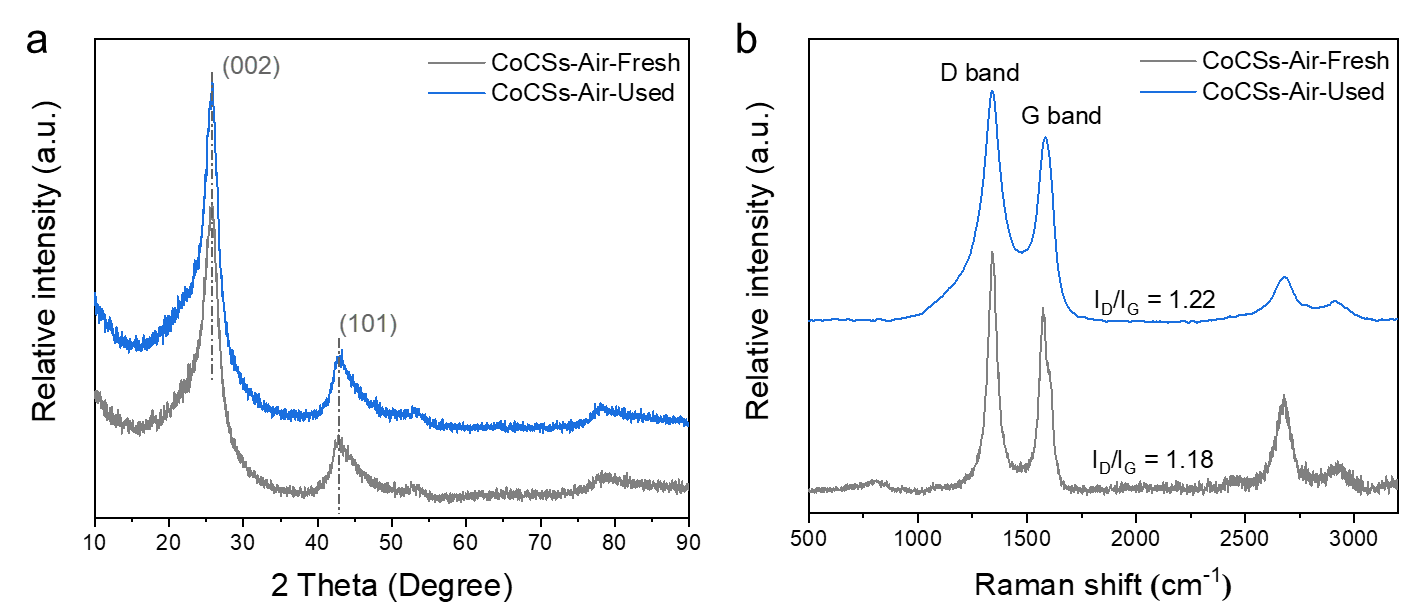
**

**Fig. S76.** (a) XRD patterns and (b) Raman spectra of CoCSs-Air-Fresh and CoCSs-Air-Used.


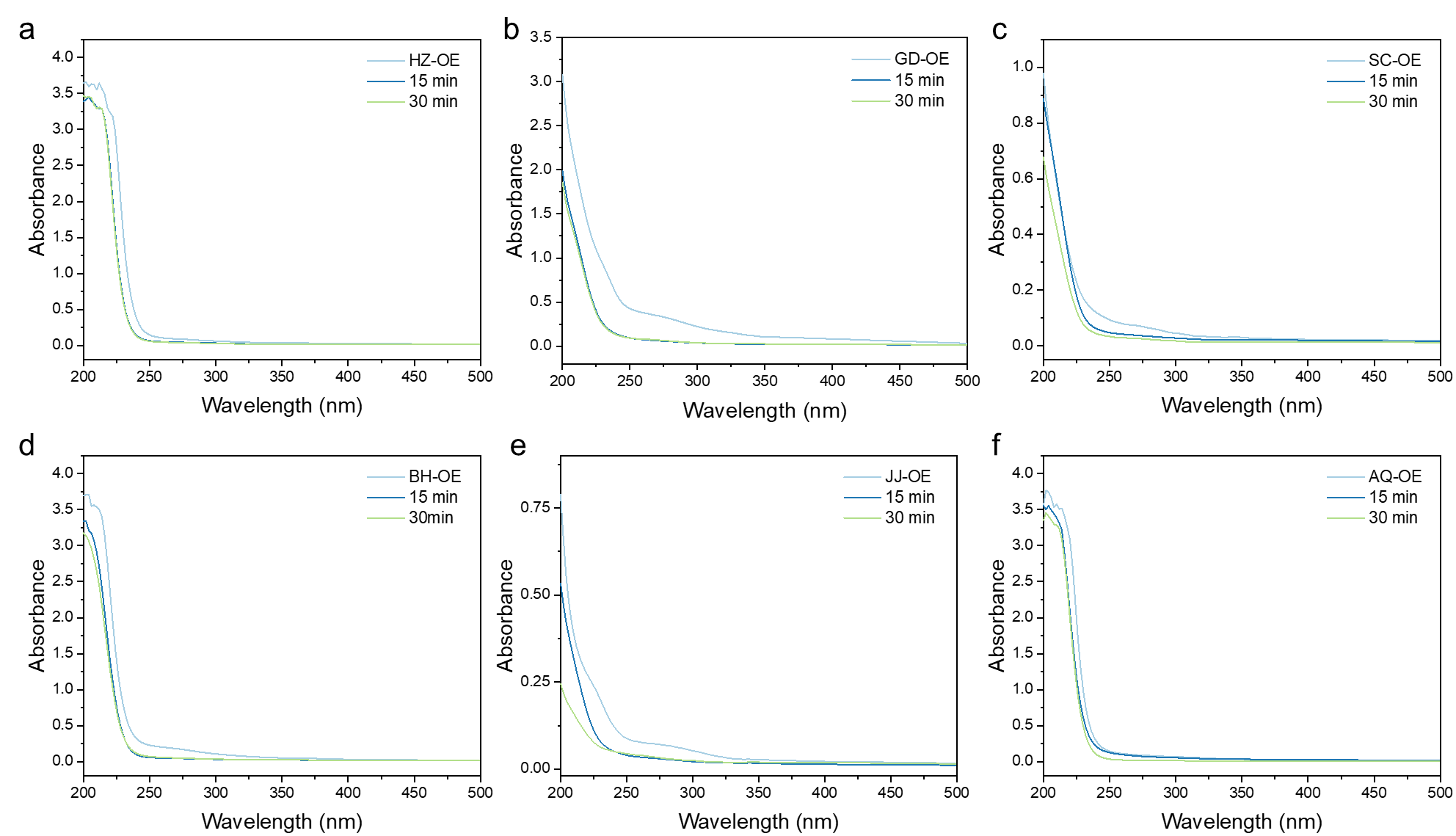


**Fig. S77.** (a-f) UV spectra of petrochemical wastewater samples before and after 15- and 30-min CoCSs-Air/O_3_ treatment.


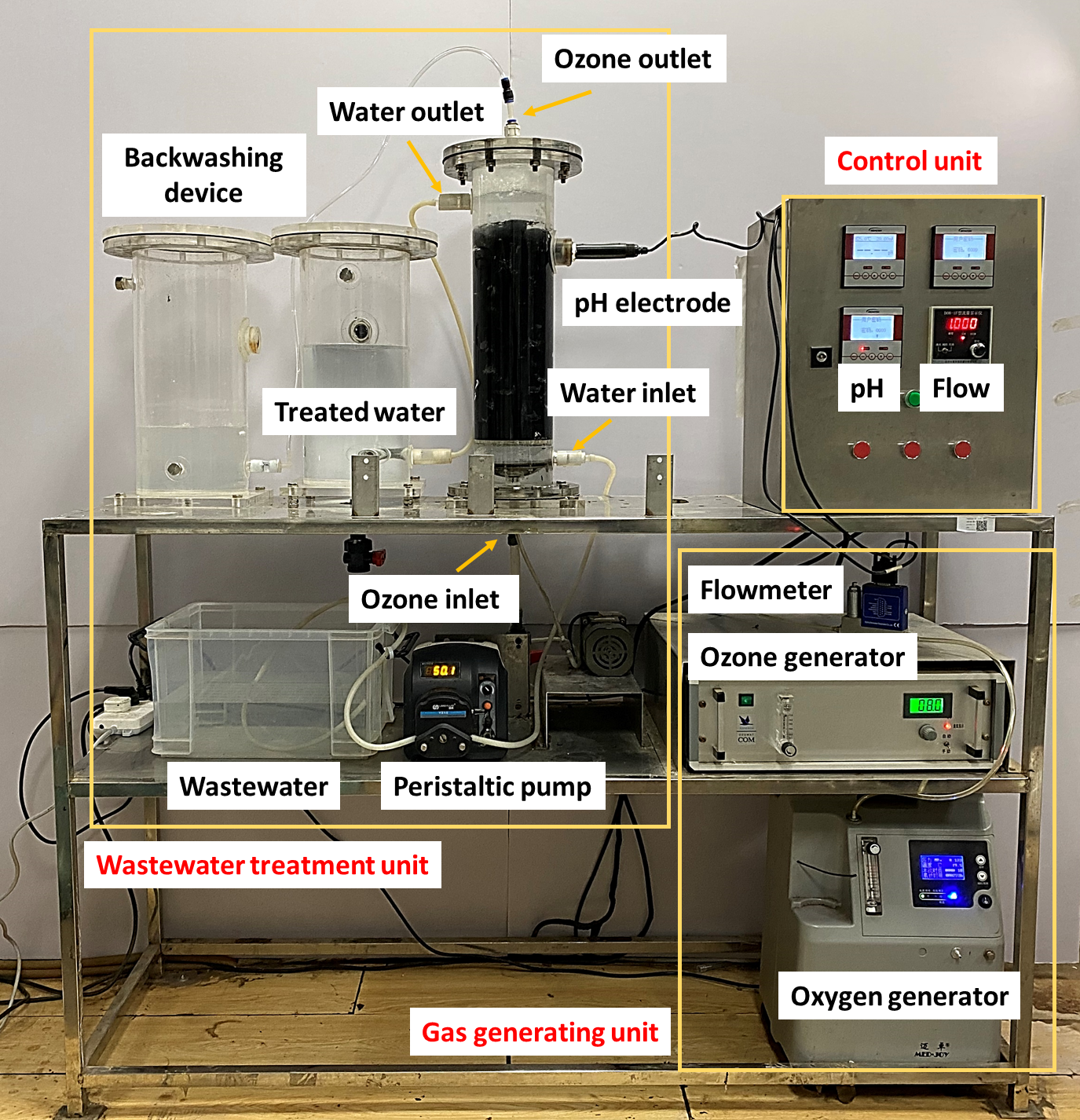


**Fig. S78.** A digital graph of the continuous flow device.

**Table S1.** **The Co contents in samples determined by ICP-MS analysis.**

| **Sample** | **Co**  **(wt.%)** | **Sample** | **Co**  **(wt.%)** |
| --- | --- | --- | --- |
| Co^2+^-CSs-NH | 0.023 | CoCSs-0.3 | 0.003 |
| Co^2+^-CSs | 0.117 | CoCSs-0.6 | 0.011 |
| CoCSs-NH | 0.002 | CoCSs-1 | 0.015 |
| CoCSs-800 | 0.002 | CoCSs-2 | 0.020 |
| CoCSs-900 | 0.004 | CoCSs-0.3-Air | 0.007 |
| CoCSs-1000 | 0.011 | CoCSs-0.6-Air | 0.022 |
| CoCSs-Air | 0.022 | CoCSs-1-Air | 0.027 |
| CoCSs-Used | 0.004 | CoCSs-2-Air | 0.037 |

**Table S2. BET specific surface areas and pore information of samples.**

| **Sample** | **S_BET_ (m^2^ g^-1^)** | **Total pore volume**  **(cm^3^ g^-1^)** | **Micropore volume**  **(cm^3^ g^-1^)** | **Mesopore volume**  **(cm^3^ g^-1^)** | **Average pore size**  **(nm)** |
| --- | --- | --- | --- | --- | --- |
| CSs | 256 | 0.33 | 0.03 | 0.30 | 6.22 |
| CoCSs-NH | 336 | 0.15 | 0.11 | 0.04 | 1.80 |
| CoCSs-800 | 518 | 0.26 | 0.17 | 0.09 | 2.01 |
| CoCSs-900 | 417 | 0.26 | 0.10 | 0.16 | 2.47 |
| CoCSs-1000 | 430 | 0.42 | 0.02 | 0.40 | 3.86 |
| CoCSs-Air | 762 | 0.56 | 0.01 | 0.55 | 2.95 |
| CoCSs-Used | 140 | 0.08 | 0.03 | 0.05 | 3.62 |
| CoCSs-Air-Used | 726 | 0.52 | 0.02 | 0.50 | 3.11 |
| CoCSs-0.3 | 396 | 0.29 | 0.08 | 0.21 | 3.31 |
| CoCSs-0.6 | 430 | 0.42 | 0.02 | 0.40 | 2.86 |
| CoCSs-1 | 413 | 0.29 | 0.08 | 0.21 | 2.04 |
| CoCSs-2 | 337 | 0.39 | 0.05 | 0.34 | 3.04 |
| CoCSs-0.3-Air | 754 | 0.58 | 0.02 | 0.38 | 3.05 |
| CoCSs-0.6-Air | 762 | 0.56 | 0.01 | 0.55 | 2.95 |
| CoCSs-1-Air | 803 | 0.57 | 0.02 | 0.55 | 2.63 |
| CoCSs-2-Air | 568 | 0.53 | 0.09 | 0.44 | 2.30 |

**Table S3. Deconvolution results for high resolution XPS surveys of C 1s for different samples.**

| **Sample** | **sp^2^ C (at%)** | **sp^3^ C (at%)** | **C-OH (at%)** | **-C=O (at%)** | **O-C=O (at%)** | **π-π shake up (at%)** |
| --- | --- | --- | --- | --- | --- | --- |
| CSs | 62.23 | 21.22 | 7.85 | 2.66 | 4.74 | 1.31 |
| CoCSs-800 | 62.08 | 20.17 | 7.45 | 3.29 | 4.63 | 2.38 |
| CoCSs-900 | 63.31 | 18.80 | 6.88 | 4.26 | 4.68 | 2.08 |
| CoCSs-1000 | 68.05 | 14.97 | 6.19 | 2.40 | 3.12 | 5.28 |
| CoCSs-Air | 70.71 | 8.63 | 6.12 | 3.34 | 1.33 | 9.87 |
| CoCSs-Used | 56.47 | 25.04 | 10.89 | 1.45 | 6.15 | - |

**Table S4. Deconvolution results for high resolution XPS surveys of O 1s for different samples.**

| **Sample** | **-OH**  **(at%)** | **-COOH**  **(at%)** | **-C=O**  **(at%)** |
| --- | --- | --- | --- |
| CSs | 47.46 | 2.88 | 46.22 |
| CoCSs | 22.58 | 9.42 | 68.00 |
| CoCSs-Air | 60.80 | 15.59 | 23.61 |
| CoCSs-0.3 | 45.11 | 5.32 | 49.57 |
| CoCSs-1 | 47.04 | 4.66 | 48.30 |
| CoCSs-2 | 41.77 | 7.39 | 50.84 |

**Table S5. Co K-edge EXAFS fitting parameters^a^ for CoCSs and CoCSs-Air.**

| **Sample** | **Path** | ***N* ^b^** | ***R* ^c^ (Å)** | ***σ*^2 d^ (Å^2^)** | **Δ*E*_0_ ^e^ (eV)** | **R factor ^f^** |
| --- | --- | --- | --- | --- | --- | --- |
| CoCSs | Co-O | 1.9(2) | 1.89(2) | 0.004(1) | 1.1±0.1 | 0.010 |
|  | Co-C | 2.1(3) | 2.08(2) | 0.004(1) |  |  |
| CoCSs-Air | Co-O | 2.0(4) | 1.89(3) | 0.004(2) | 2.3±0.2 | 0.005 |
|  | Co-C | 1.9(2) | 2.09(4) | 0.004(2) |  |  |

^a^ S_0_^2^ was fixed as 0.78, according to the experimental EXAFS fit of Co foil by fixing *N* as the known crystallographic value. Data ranges: 2 ≤ *k* ≤ 11 Å^-1^, 1 ≤ *R* ≤ 2 Å. ^b^ *N*: coordination number. ^c^ *R*, distance between absorber and backscatter atoms. ^d^ *σ*^2^: Debye-Waller factor to account for both thermal and structural disorders. The Debye-Waller factors were constrained as *σ*^2^(Co-O) = *σ*^2^(Co-C) for decreasing the correlation (or reducing the number of variables). ^e^ *ΔE*_0_: inner potential correction. *ΔE*_0_ was defined as a global fit parameter. ^f^ R factor indicates the goodness of the fit. A reasonable range of EXAFS fitting parameters: 0.7 < *Ѕ*_0_^2^ < 1.0; *N >* 0; *σ*^2^ > 0 Å^2^; |Δ*E*_0_| < 10 eV; *R* factor < 0.02.

**Table S6. Elemental contents of samples by EA analysis.**

| **Sample** | **C (wt.%)** | **O (wt.%)** |
| --- | --- | --- |
| CSs | 93.3 | 4.4 |
| CoCSs | 94.2 | 3.9 |
| CoCSs-Air | 87.3 | 11.3 |
| CoCSs-Air-480 | 93.7 | 5.4 |
| CoCSs-Air-490 | 91.6 | 6.9 |
| CoCSs-Air-500 | 88.5 | 10.4 |
| CoCSs-Used | 84.2 | 15.4 |

**Table S7. Comparison of catalytic activities of CoCSs-Air with previously reported catalysts.**

| **Catalyst** | **Catalyst dosage**  **(g L^-1^)** | **O_3_**  **concentration**  **(mg L^-1^)** | **Pollutant** | **Pollutant**  **concentration**  **(mg L^-1^)** | **Removal**  **efficiency**  **(%)** | **Time**  **(min)** | **S_BET_**  **(m^2^ g^-1^)** | **EF**  **(L g^-1^ min^-1^)** | **S_BET_ normalized EF**  **(L min^-1^ m^-2^)** | **Ref.** |
| --- | --- | --- | --- | --- | --- | --- | --- | --- | --- | --- |
| CoCSs-Air | 0.01 | 15 | Oxalic acid | 150 | 90 | 20 | 762 | 44 | 0.062 | **This work** |
| Co-N@CNTs | 0.1 | 25 | Oxalic acid | 50 | 100 | 20 | 126 | 1 | 0.008 | ^[14]^ |
| NSC | 0.1 | 25 | Benzimidazole | 50 | 100 | 45 | 708 | 0.44 | 0.001 | ^[15]^ |
| rGO | 0.1 | 20 | p-hydroxylbenzoic acid | 20 | 100 | 30 | 265 | 0.33 | 0.001 | ^[16]^ |
| LIB-rGO | 0.1 | 50 | Oxalic acid | 50 | 100 | 45 | 362 | 0.22 | 0.001 | ^[17]^ |
| MWI-rGO-N | 0.1 | 50 | Oxalic acid | 50 | 100 | 20 | 200 | 0.5 | 0.003 | ^[18]^ |
| MWCNT | 0.1 | 42 | Oxalic acid | 90 | 80 | 40 | 118 | 0.42 | 0.004 | ^[19]^ |
| F-CNTs | 0.05 | 20 | Oxalic acid | 180 | 80 | 90 | 198 | 1.6 | 0.008 | ^[20]^ |
| N3C | 0.1 | 25 | Oxalic acid | 50 | 100 | 45 | 79 | 0.44 | 0.006 | ^[21]^ |
| CPG-2 | 0.1 | 25 | Oxalic acid | 50 | 100 | 20 | 163 | 1 | 0.006 | ^[22]^ |
| NHC | 0.01 | 3.6 | Ketoprofen | 1 | 100 | 30 | 586 | 0.92 | 0.002 | ^[23]^ |
| OA-CN | 0.2 | 20 | Atrazine | 2 | 100 | 20 | 66 | 0.02 | 0.000 | ^[24]^ |
| ND | 0.1 | 4.1 | Oxalic acid | 50 | 100 | 180 | 311 | 0.67 | 0.002 | ^[25]^ |
| NBC | 0.04 | 9 | Atrazine | 1 | 70 | 15 | 544 | 0.12 | 0.000 | ^[26]^ |
| Mn-CSF | 0.2 | 10 | Oxalic acid | 100 | 90 | 60 | 149.1 | 0.75 | 0.005 | ^[27]^ |
| Fe_5_-NC | 0.2 | 10 | Oxalic acid | 100 | 90 | 60 | 849 | 0.75 | 0.001 | ^[28]^ |
| MnO_2_/rGO | 0.1 | 50 | 4-nitrophenol | 50 | 100 | 30 | 35 | 0.33 | 0.010 | ^[29]^ |
| ZVZ-g-C_3_N_4_ | 0.5 | 10 | Atrazine | 2 | 100 | 3 | 41 | 0.13 | 0.003 | ^[30]^ |
| CuO-CN | 0.5 | 10 | Oxalic acid | 50 | 100 | 30 | 21 | 0.33 | 0.016 | ^[31]^ |
| PdO/CeO_2_ | 0.15 | 21 | Oxalic acid | 18 | 35 | 12 | 94 | 0.16 | 0.002 | ^[32]^ |
| Iron silicate | 0.1 | 0.9 | 4-chloronitrobenzene | 0.1 | 90 | 30 | 203 | 0.03 | 0.000 | ^[33]^ |
| Bio-FeMnCoO_x_ | 0.5 | 11.3 | Benzotriazole | 100 | 100 | 20 | 98 | 0.88 | 0.009 | ^[34]^ |
| N-doped ceria | 0.6 | 1 | Sulfamethoxazole | 50 | 95 | 30 | 82 | 2.63 | 0.032 | ^[35]^ |
| Ov‑Bi_2_O_3_ | 0.5 | 36 | p-nitrophenol | 140 | 80 | 300 | 26 | 0.02 | 0.001 | ^[36]^ |
| Zinc ferrite spinel | 0.2 | 30 | Ibuprofen | 20 | 60 | 90 | 27 | 0.02 | 0.001 | ^[37]^ |
| CeO_2_ nanorods | 2.5 | 50 | Phenol | 100 | 100 | 60 | 82 | 0.01 | 0.000 | ^[38]^ |
| ZnO_x_ | 0.04 | 58 | Atrazine | 1 | 95 | 15 | 5 | 0.03 | 0.005 | ^[39]^ |
| ZNC400 | 0.02 | 10 | Atrazine | 1.08 | 100 | 15 | 26 | 0.360 | 0.014 | ^[40]^ |
| CoSAC-N-C | 0.05 | 20 | Oxalic acid | 200 | 100 | 60 | 511.4 | 3.333 | 0.007 | ^[41]^ |

**Table S8.** **Summary of the impedance fitting data.**

| **Sample** | **R_S_**  **(Ω)** | **R_p_**  **(Ω)** | **CPE_p_**  **(mF)** | **R_film_**  **(Ω)** | **CPE_film_**  **(mF)** | **R_CT_**  **(Ω)** | **CPE_CT_ (mF)** | **R_total_ (Ω)** |
| --- | --- | --- | --- | --- | --- | --- | --- | --- |
| CSs | 29.69 | 66.92 | 5.89×10^-9^ | 6.0×10^6^ | 1.89×10^-5^ | 1988 | 0.0192 | 50.99 |
| CoCSs | 23.85 | 63.42 | 6.96×10^-9^ | 4.8×10^6^ | 2.32×10^-4^ | 899.5 | 0.0085 | 41.98 |
| CoCSs-Air | 18.01 | 57.07 | 5.55×10^-9^ | 4.1×10^6^ | 1.27×10^-3^ | 469.3 | 0.0189 | 38.18 |

**Table S9. Computed bond length of different models.**

| **Model** | **In-plane** | | | | **Out-plane** | | | |
| --- | --- | --- | --- | --- | --- | --- | --- | --- |
|  | **Co-O1** | **Co-O2** | **Co-C1** | **Co-C2** | **Co-O1** | **Co-O2** | **O1-O3** | **O2-O3** |
| Co-C_2_O_2_ | 1.985 Å | 1.971 Å | 1.876 Å | 1.853 Å |  |  |  |  |
| Co-*O | 2.112 Å | 2.072 Å | 1.896 Å | 1.852 Å | 1.629 Å |  |  | 1.236 Å |
| Co-*O_3_ | 2.304 Å | 2.260 Å | 1.957 Å | 1.933 Å | 1.976 Å | 2.115 Å | 1.404 Å | 1.356 Å |
| free O_3_ molecule |  |  |  |  |  |  | 1.278 Å | 1.278 Å |

**Table S10. The calculated magnetic moments of Co sites.**

| **Model** | **Magnetic moment (u_eff_)** |
| --- | --- |
| Co-C_2_O_2_ | 0.95 |
| Co-*O | 0.81 |
| Co-*O_3_ | 1.73 |
| Co-*O + OA | 1.10 |
| Co-*O_3_ + OA | 0.31 |

**Table S11. The calculated Co 3d-band center.**

| **Model** | **Spin up** | **Spin down** | **Δd** | **Average** |
| --- | --- | --- | --- | --- |
| Co-C_2_O_2_ | -1.03 | -0.14 | 0.89 | -0.58 |
| Co-*O | -2.60 | -1.80 | 0.80 | -2.20 |
| Co-*O_3_ | -1.93 | -0.26 | 1.67 | -1.10 |
| Co-*O + OA | -2.05 | -1.42 | 0.63 | -1.74 |
| Co-*O_3_ + OA | -1.63 | -0.57 | 1.06 | -1.10 |

**Table S12. The integrated crystal orbital Hamilton populations (ICOHP) of the Co-O bond.**

| **Model** | **Spin state** | **ICOHP** |
| --- | --- | --- |
| Co-*O | Spin up | -2.06 |
|  | Spin down | -3.21 |
| Co-*O_3_ | Spin up | -1.29 |
|  | Spin down | -2.20 |

**Table S13. The integral quantity of pDOS.**

| **Model** | **Orbital** | **Spin up** | **Spin down** | **Total** |
| --- | --- | --- | --- | --- |
| Co-C_2_O_2_ | 3d_xy_ | 0.491 | 0.432 | 0.923 |
|  | 3d_xz_ | 0.850 | 0.741 | 1.591 |
|  | 3d_yz_ | 0.917 | 0.285 | 1.202 |
|  | 3$\text{d}_{\text{z}^{\text{2}}}$ | 0.874 | 0.754 | 1.628 |
|  | 3$\text{d}_{\text{x}^{\text{2}}\text{-}\text{y}^{\text{2}}}$ | 0.920 | 0.911 | 1.831 |
|  | Total | 4.052 | 3.123 | 7.175 |
| Co-*O | 3d_xy_ | 0.630 | 0.495 | 1.125 |
|  | 3d_xz_ | 0.880 | 0.625 | 1.505 |
|  | 3d_yz_ | 0.903 | 0.611 | 1.514 |
|  | 3$\text{d}_{\text{z}^{\text{2}}}$ | 0.607 | 0.558 | 1.165 |
|  | 3$\text{d}_{\text{x}^{\text{2}}\text{-}\text{y}^{\text{2}}}$ | 0.927 | 0.919 | 1.846 |
|  | Total | 3.947 | 3.208 | 7.155 |
| Co-*O_3_ | 3d_xy_ | 0.727 | 0.405 | 1.132 |
|  | 3d_xz_ | 0.941 | 0.281 | 1.222 |
|  | 3d_yz_ | 0.850 | 0.622 | 1.472 |
|  | 3$\text{d}_{\text{z}^{\text{2}}}$ | 0.910 | 0.583 | 1.493 |
|  | 3$\text{d}_{\text{x}^{\text{2}}\text{-}\text{y}^{\text{2}}}$ | 0.931 | 0.871 | 1.802 |
|  | Total | 4.359 | 2.762 | 7.121 |

**Table S14. The component of atomic orbital of Co-O bond.**

| **Orbital** | **Co-*O** | | **Co-*O_3_** | |
| --- | --- | --- | --- | --- |
|  | **Spin up** | **Spin down** | **Spin up** | **Spin down** |
| 3d_xy_-2p | 0.37 % | 0.11 % | Co-O1: 28.27 %  Co-O2: 13.38 % | Co-O1: 3.10 %  Co-O2: 3.24 % |
| 3d_xz_-2p | 10.71 % | 29.38 % | Co-O1: 22.37 %  Co-O2: 9.78 % | Co-O1: 56.07 %  Co-O2: 41.21 % |
| 3d_yz_-2p | 9.14 % | 29.73 % | Co-O1: 24.72 %  Co-O2: 34.84 % | Co-O1: 19.87 %  Co-O2: 23.36 % |
| 3$\text{d}_{\text{z}^{\text{2}}}$-2p | 79.69 % | 40.63 % | Co-O1: 14.67 %  Co-O2: 37.55 % | Co-O1: 10.16 %  Co-O2: 28.90 % |
| 3$\text{d}_{\text{x}^{\text{2}}\text{-}\text{y}^{\text{2}}}$-2p | 0.09 % | 0.15 % | Co-O1: 9.97 %  Co-O2: 4.45 % | Co-O1: 10.80 %  Co-O2: 3.29 % |

**Table S15. Water matrix characteristics of the PCW samples tested in this work.**

| **Sample** | **Source** | **Characteristics (Unit: mg L^-1^)** | | | | | | | | | |
| --- | --- | --- | --- | --- | --- | --- | --- | --- | --- | --- | --- |
|  |  | **pH** | **COD** | **Cl^-^** | **SO_4_^2-^** | **NO_3_^-^** | **Na^+^** | **NH_4_^+^** | **K^+^** | **Mg^2+^** | **Ca^2+^** |
| HZ-AI | Huizhou Refinery | 7.6 | 285.5 | 286.2 | 82.7 | - | 280.1 | - | - | - | 43.1 |
| HZ-AE |  | 7.5 | 46.4 | 286.7 | 105.5 | - | 281.9 | - | - | - | 40.8 |
| HZ-OE |  | 8.4 | 56.5 | - | 585.2 | 229.0 | 277.5 | - | - | - | 41.3 |
| GD-AI | Guangdong Petrochemical | 8.8 | 137.9 | 1298.2 | 3737.4 | - | 3914.3 | - | 185.3 | 36.4 | 48.3 |
| GD-AE |  | 8.6 | 108.9 | 1608.7 | 4538.5 | - | 4007.8 | - | 188.4 | 33.3 | 56.0 |
| GD-OE |  | 8.5 | 115.7 | 1459.1 | 4167.0 | - | 4053.8 | - | 182.9 | 36.0 | 44.0 |
| SC-AI | Sichuan Petrochemical | 8.4 | 198.5 | 119.7 | 1151.7 | - | 787.5 | - | - | 13.7 | 107.0 |
| SC-AE |  | 8.4 | 76.8 | 107.0 | 1060.9 | 49.1 | 836.5 | - | - | 12.2 | 97.2 |
| SC-OE |  | 8.2 | 91.7 | 96.5 | 1059.5 | 54.8 | 850.2 | - | - | 8.5 | 73.0 |
| BH-AI | Beihai Refinery | 7.7 | 334.8 | 408.6 | 971.1 | - | 1034.6 | 4.0 | 3.1 | - | 37.0 |
| BH-AE |  | 8.6 | 96.6 | 489.7 | 616.4 | 57.1 | 869.4 | - | 8.8 | 1.2 | 58.8 |
| BH-OE |  | 7.8 | 82.3 | 555.6 | 645.1 | 81.1 | 832.9 | - | 12.7 | 0.2 | 53.7 |
| JJ-AI | Jiujiang Petrochemical | 8.1 | 404.6 | 9228.5 | 7929.6 | 1323.3 | 471.2 | 33.9 | 10.4 | 18.6 | 468.3 |
| JJ-AE |  | 8.0 | 63.4 | 611.0 | 430.8 | 161.5 | 546.7 | - | 14.6 | 15.3 | 113.2 |
| JJ-OE |  | 8.2 | 37.9 | 624.4 | 412.3 | 50.9 | 245.9 | - | 5.2 | 7.6 | 55.1 |
| AQ-AI | Anqing Petrochemical | 8.4 | 272.1 | 75.6 | 383.8 | 10.2 | 1283.4 | 57.3 | 18.7 | 13.9 | 65.2 |
| AQ-AE |  | 8.6 | 96.3 | 394.0 | 1811.5 | 117.6 | 1133.5 | 9.9 | 15.5 | 14.9 | 65.8 |
| AQ-OE |  | 8.1 | 85.6 | 101.5 | 117.5 | 102.7 | 3684.7 | - | 13.7 | 13.4 | 58.5 |

**Table S16. Performance of CoCSs-Air/O_3_ system for treating PCW samples.**

| **Sample** | **COD before treatment**  **(mg L^-1^)** | **COD after treatment**  **(mg L^-1^)** | **Removal efficiency** |
| --- | --- | --- | --- |
| HZ-AI | 285.5 | 90.3 | 68.4% |
| HZ-AE | 46.4 | 18.8 | 59.5% |
| HZ-OE | 56.5 | 21.6 | 61.8% |
| GD-AI | 137.9 | 63.2 | 54.2% |
| GD-AE | 108.9 | 45.1 | 58.6% |
| GD-OE | 115.7 | 45.8 | 60.4% |
| SC-AI | 198.5 | 63.2 | 68.2% |
| SC-AE | 76.8 | 24.2 | 68.5% |
| SC-OE | 91.7 | 27.1 | 70.4% |
| BH-AI | 334.8 | 119.7 | 64.2% |
| BH-AE | 96.6 | 32.4 | 66.5% |
| BH-OE | 82.3 | 30.5 | 62.9% |
| JJ-AI | 404.6 | 216.7 | 46.4% |
| JJ-AE | 63.4 | 35.6 | 43.8% |
| JJ-OE | 37.9 | 15.8 | 58.3% |
| AQ-AI | 272.1 | 112.9 | 58.5% |
| AQ-AE | 96.3 | 22.6 | 76.5% |
| AQ-OE | 85.6 | 21.1 | 75.4% |

**Table S17. The cost of energy consumption for COD elimination of PCW using different reaction systems based on the EE/O concept.**

| **Systems** | **Initial COD concentration (mg L^-1^)** | **COD removal** | **EE/O**  **(kW·h m^-3^)** | **Cost**  **(USD m^-3^)** |
| --- | --- | --- | --- | --- |
| Single ozonation | 404.6 | 12.7% | 6.61 | 0.463 |
| CSs/O_3_ | 404.6 | 13.8% | 6.11 | 0.428 |
| CoCSs/O_3_ | 404.6 | 31.4% | 2.56 | 0.179 |
| CoCSs-Air/O_3_ | 404.6 | 69.6% | 0.76 | 0.053 |

**Table S18. Cost assessment of catalyst fabrication.**

| **Item** | **Unit-price** | **Dosage** | **Cost**  **(USD)** | **Total**  **(USD m^-3^)** |
| --- | --- | --- | --- | --- |
| Glucose | 0.001 USD g^-1^ | 7.24 g | 0.00724 | 0.02 |
| CoCl_2_**^.^**6H_2_O | 0.126 USD g^-1^ | 0.009 g | 0.00113 |  |
| Hydrochloric acid | 0.006 USD mL^-1^ | 10 mL | 0.06 |  |
| Magnetic stirrers | 0.07 USD kW·h^-1^ | 1 kW·h | 0.07 |  |
| Oven | 0.07 USD kW·h^-1^ | 25 kW·h | 1.75 |  |
| Muffle furnace | 0.07 USD kW·h^-1^ | 14 kW·h | 0.98 |  |
| Tube furnace | 0.07 USD kW·h^-1^ | 22 kW·h | 1.54 |  |

**Equations**

*O_3_ + O_3_ → *O_2_ + 2O_2_ **Eq. S1**

*O + O_3_ → *O_2_ + O_2_ **Eq. S2**

*O_2_ + 2H^+^ + 2e^–^ → H_2_O_2_ **Eq. S3**

*O_3_ + H_2_O → 2*OH + O_2_ **Eq. S4**

*O + H_2_O → 2*OH **Eq. S5**

*OH + *OH → H_2_O_2_ **Eq. S6**

**References**

[1] Y. Wang, Z. Ao, H. Sun, X. Duan, S. Wang, *Appl. Catal. B-Environ.* **2016**, *198*, 295-302.

[2] H. Bader, J. Hoigné, *Water Res.* **1981**, *15*, 449-456.

[3] H. Bader, V. Sturzenegger, J. Hoigné, *Water Res.* **1988**, *22*, 1109-1115.

[4] G. Kresse, D. Joubert, *Phys. Rev. B* **1999**, *59*, 1758-1775.

[5] J. P. Perdew, K. Burke, M. Ernzerhof, *Phys. Rev. Lett.* **1996**, *77*, 3865-3868.

[6] S. Grimme, J. Antony, S. Ehrlich, H. Krieg, *J. Chem. Phys.* **2010**, *132*, 154104.

[7] W. Tang, E. Sanville, G. Henkelman, *J. Phys.: Condens. Matter* **2009**, *21*, 084204.

[8] a) V. L. Deringer, A. L. Tchougréeff, R. Dronskowski, *J. Phys. Chem. A* **2011**, *115*, 5461-5466; b) R. Dronskowski, P. E. Blöchl, *J. Phys. Chem.* **1993**, *97*, 8617-8624.

[9] R. Nelson, C. Ertural, J. George, V. L. Deringer, G. Hautier, R. Dronskowski, *J. Comput. Chem.* **2020**, *41*, 1931-1940.

[10] a) T. Ozaki, *Phys. Rev. B* **2003**, *67*, 155108; b) T. Ozaki, H. Kino, *Phys. Rev. B* **2004**, *69*, 195113.

[11] J. R. Bolton, K. G. Bircher, W. Tumas, C. A. Tolman, *J. Adv. Oxid. Technol.* **1996**, *1*, 13-17.

[12] V. V. Pushkarev, V. I. Kovalchuk, J. L. d'Itri, *J. Phys. Chem. B* **2004**, *108*, 5341-5348.

[13] R. Radhakrishnan, S. T. Oyama, *J. Catal.* **2001**, *199*, 282-290.

[14] Y. Wang, N. Ren, J. Xi, Y. Liu, T. Kong, C. Chen, Y. Xie, X. Duan, S. Wang, *ACS ES&T Eng.* **2021**, *1*, 32-45.

[15] Y. Xie, Y. Liu, Y. Yao, Y. Shi, B. Zhao, Y. Wang, *Chin. Chem. Lett.* **2022**, *33*, 1298-1302.

[16] Y. Wang, Y. Xie, H. Sun, J. Xiao, H. Cao, S. Wang, *ACS Appl. Mater. Interfaces* **2016**, *8*, 9710-9720.

[17] Y. Wang, H. Cao, L. Chen, C. Chen, X. Duan, Y. Xie, W. Song, H. Sun, S. Wang, *Appl. Catal. B-Environ.* **2018**, *229*, 71-80.

[18] Y. Wang, H. Cao, C. Chen, Y. Xie, H. Sun, X. Duan, S. Wang, *Chem. Eng. J.* **2019**, *355*, 118-129.

[19] Z.-Q. Liu, J. Ma, Y.-H. Cui, B.-P. Zhang, *Appl. Catal. B-Environ.* **2009**, *92*, 301-306.

[20] J. Wang, S. Chen, X. Quan, H. Yu, *Chemosphere* **2018**, *190*, 135-143.

[21] Y. Wang, L. Chen, C. Chen, J. Xi, H. Cao, X. Duan, Y. Xie, W. Song, S. Wang, *Appl. Catal. B-Environ.* **2019**, *254*, 283-291.

[22] Y. Wang, J. Xi, X. Duan, W. Lv, H. Cao, C. Chen, Z. Guo, Y. Xie, S. Wang, *J. Hazard. Mater.* **2020**, *384*, 121486.

[23] Z. Sun, L. Zhao, C. Liu, Y. Zhen, J. Ma, *Environ. Sci. Technol.* **2019**, *53*, 10342-10351.

[24] J. Zhang, B. Xin, C. Shan, W. Zhang, D. D. Dionysiou, B. Pan, *Appl. Catal. B-Environ.* **2021**, *292*, 120155.

[25] F. Bernat-Quesada, C. Vallés-García, E. Montero-Lanzuela, A. López-Francés, B. Ferrer, H. G. Baldoví, S. Navalón, *Appl. Catal. B-Environ.* **2021**, *299*, 120673.

[26] Y. Cheng, B. Wang, P. Yan, J. Shen, J. Kang, S. Zhao, X. Zhu, L. Shen, S. Wang, Y. Shen, Z. Chen, *Chem. Eng. J.* **2023**, *454*, 140232.

[27] S. Chen, T. Ren, X. Zhang, Z. Zhou, X. Huang, X. Zhang, *Sci. Total Environ.* **2023**, *858*, 159447.

[28] T. Ren, M. Yin, S. Chen, C. Ouyang, X. Huang, X. Zhang, *Environ. Sci. Technol.* **2023**, *57*, 3623-3633.

[29] Y. Wang, Y. Xie, H. Sun, J. Xiao, H. Cao, S. Wang, *J. Hazard. Mater.* **2016**, *301*, 56-64.

[30] X. Yuan, W. Qin, X. Lei, L. Sun, Q. Li, D. Li, H. Xu, D. Xia, *Chemosphere* **2018**, *205*, 369-379.

[31] J. Liu, J. Li, S. He, L. Sun, X. Yuan, D. Xia, *Sep. Purif. Technol.* **2020**, *234*, 116120.

[32] T. Zhang, W. Li, J.-P. Croué, *Environ. Sci. Technol.* **2011**, *45*, 9339-9346.

[33] L. Yuan, J. Shen, P. Yan, Z. Chen, *Environ. Sci. Technol.* **2018**, *52*, 1429-1434.

[34] A. Xu, S. Fan, T. Meng, R. Zhang, Y. Zhang, S. Pan, Y. Zhang, *Appl. Catal. B-Environ.* **2022**, *318*, 121833.

[35] S. Zhan, H. Huang, C. He, Y. Xiong, P. Li, S. Tian, *Appl. Catal. B-Environ.* **2023**, *321*, 122040.

[36] G. Zhai, S. Liu, S. Si, Y. Liu, H. Zhang, Y. Mao, M. Zhang, Z. Wang, H. Cheng, P. Wang, Z. Zheng, Y. Dai, B. Huang, *ACS ES&T Water* **2022**, *2*, 1725-1733.

[37] L. Liang, P. Cao, X. Qin, S. Wu, H. Bai, S. Chen, H. Yu, Y. Su, X. Quan, *Appl. Catal. B-Environ.* **2023**, *325*, 122321.

[38] L. Wu, J. Wang, C. Yang, X. Gao, Y. Fang, X. Wang, W. D. Wu, Z. Wu, *Appl. Catal. B-Environ.* **2023**, *323*, 122152.

[39] Y. Cheng, J. Kang, P. Yan, J. Shen, Z. Chen, X. Zhu, Q. Tan, L. Shen, S. Wang, S. Wang, *Appl. Catal. B-Environ.* **2024**, *341*, 123325.

[40] Y. Cheng, Z. Chen, P. Yan, J. Shen, J. Kang, S. Wang, X. Duan, *ACS Catal.* **2024**, *14*, 4040-4052.

[41] L. Liang, P. Cao, H. Bai, X. Qin, Z. Lu, S. Chen, Y. Liu, H. Yu, X. Quan, *Appl. Catal. B-Environ.* **2024**, *354*, 124149.
